# The facile construction of the phthalazin-1(2*H*)-one scaffold via copper-mediated C–H(sp^2^)/C–H(sp) coupling under mild conditions

**DOI:** 10.3762/bjoc.11.177

**Published:** 2015-09-14

**Authors:** Wei Zhu, Bao Wang, Shengbin Zhou, Hong Liu

**Affiliations:** 1 CAS Key Laboratory of Receptor Research, Shanghai Institute of Materia Medica, Chinese Academy of Sciences, 555 Zuchongzhi Road, Shanghai 201203, P. R. China

**Keywords:** C–H activation, copper, phthalazin-1(2*H*)-one

## Abstract

A novel strategy for the construction of the phthalazin-1(2*H*)-one scaffold has been developed by means of a copper-mediated cascade C–H/C–H coupling and intramolecular annulations and a subsequent facile hydrazinolysis. This C–H activation transformation proceeds smoothly with wide generality, good functional tolerance and high stereo- and regioselectivity under mild conditions. Through the removal of the directing group, the resulting moiety could easily be transformed into the phthalazin-1(2*H*)-one scaffold, which is known to be a privileged moiety and a bioactive nucleus in pharmaceuticals.

## Introduction

**Figure 1:**
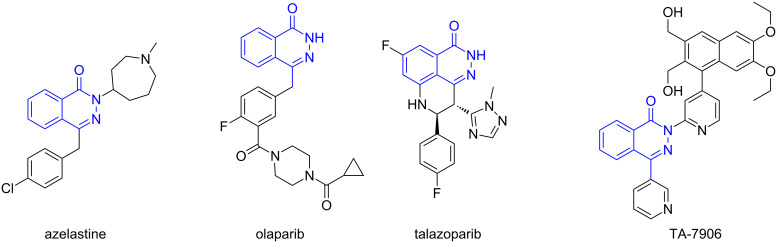
Representative structures of biologically important phthalazin-1(2*H*)-ones.

The phthalazin-1(2*H*)-one scaffold represents an important class of privileged structures and has been widely found in numerous biologically active compounds and drug molecules [1–6]. For example (Figure 1), azelastine is a selective histamine antagonist, which is recommended for the treatment of both seasonal allergic rhinitis (SAR) and nonallergic vasomotor rhinitis (VMR) [7]. As the lead of a series of poly ADP ribose polymerase (PARP) inhibitors, olaparib has been approved by the FDA as a potential treatment for germline BRCA mutated (gBRCAm) advanced ovarian cancer [8]; another PARP inhibitor, talazoparib, is undergoing phase III trials [9]. Meanwhile, TA-7906 has been developed as a phosphodiesterase-4 (PDE4) inhibitor and is undergoing phase II trials [10]. Although several methods have been developed for the synthesis of phthalazin-1(2*H*)-one derivatives, these approaches mainly rely on the use of prefunctionalized benzoic acid, such as phthalic anhydride or 2-formylbenzoic acid, as starting materials and suffer from low yields, poor regioselectivity and scope limitations [11–12]. Therefore, there is still a need for synthetic chemists to develop efficient and expedient routes for the construction of the phthalazin-1(2*H*)-one scaffold.

During the last decade, the transition metal-mediated C–H activation reaction has emerged as one of the most important and powerful methodologies for the formation of carbon–carbon bonds in a single synthetic operation [13–16]. From the viewpoint of practicality and feasibility, increasing attention was paid to develop cheap and easily available transition metals, such as copper, to realize direct C–H functionalization [17]. Miura and Yu successfully demonstrated a novel protocol for the direct C–H/C–H coupling with combinations of copper salts and *N*,*N*’-dual coordinated directing groups [18–22]. By the employment of a similar approach, our group reported a straightforward route to the isoquinolinone scaffold via copper-mediated C–H(sp^2^)/C–H(sp^3^) coupling [23].

**Scheme 1:**
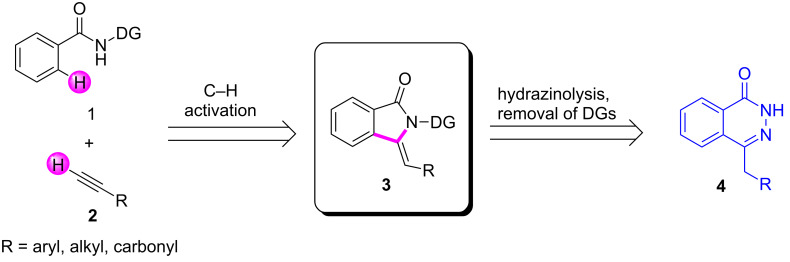
The construction of phthalazin-1(2*H*)-one scaffold via C–H activation.

In light of previous works, we envisioned the construction of the phthalazin-1(2*H*)-one scaffold by the hydrazinolysis of precursor **3**, which could be afforded by copper-mediated C–H(sp^2^)/C–H(sp) direct coupling and sequential annulations (Scheme 1). During our exploration, however, You reported a similar C–H activation transformation system at high temperature (120 °C) with excessive copper salts (3 equivalents). In addition, failure to remove the directing group partly prevented the practicality and application of the transformation [22]. Herein, we reveal a copper-mediated direct C–H(sp^2^)-C–H(sp) bond construction and simultaneous annulations under milder conditions with less equivalents of copper salts and oxygen atmosphere at a lower temperature (80 °C). Even more important, the resulting products could be smoothly transformed into preconceived 4-benzylphthalazin-1(2*H*)-one derivatives with the removal of the directing group by treatment with hydrazine hydrate and sodium hydroxide.

## Results and Discussion

We initiated our investigation of the direct carbon–carbon coupling of *N*-(quinolin-8-yl)benzamide (**1a**) and phenylacetylene (**2a**). After extensive attempts, 3-benzylidene-2-(quinolin-8-yl)isoindolin-1-one (**3a**) was formed in 18% yield via the combination of Cu(OAc)_2_ and Li_2_CO_3_ in DMF under an oxygen atmosphere (Table 1, entry 1). As shown in Table 1, various bases, copper(II) salts and solvents were screened for the best reaction conditions. With Cu(OAc)_2_ as the transition metal and DMF as the solvent at 90 °C under oxygen atmosphere, we tested the impact of various inorganic bases and K_2_CO_3_ proved to be the most efficient promoter (Table 1, entries 1–7). The subsequent evaluation of copper salts revealed that Cu(OAc)_2_ gave the best yield (Table 1, entries 8–11). The effect of different solvents on the transformation was also investigated. DMSO was slightly inferior to DMF, while the use of the aprotic solvent iPrOH and the nonpolar solvent DCE was ineffective (Table 1, entries 12–14). The extension of the reaction time could not provide higher yield (Table 1, entry 15). The absence of oxygen or a decrease in the amount of Cu(OAc)_2_ led to dramatic reductions in yields (Table 1, entries 16 and 17). Gratifyingly, the temperature screening showed that the transformation yield was further enhanced by reducing the reaction temperature to 80 °C (Table 1, entry 18). The reaction could also be conducted under the atmosphere of air (Table 1, entry 19). In addition, single-crystal X-ray diffraction of **3a** showed that the *Z*-isomer of the alkene is preferentially formed [24].

**Table 1:** Optimization of reaction conditions.^a^

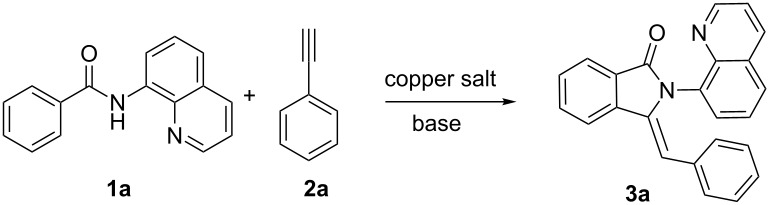				

Entry	Copper salt	Base	Solvent	Yield^b^

1	Cu(OAc)_2_	Li_2_CO_3_	DMF	18%
2	Cu(OAc)_2_	Na_2_CO_3_	DMF	43%
3	Cu(OAc)_2_	K_2_CO_3_	DMF	78%
4	Cu(OAc)_2_	Cs_2_CO_3_	DMF	74%
5	Cu(OAc)_2_	KOAc	DMF	31%
6	Cu(OAc)_2_	*t*-BuONa	DMF	16%
7	Cu(OAc)_2_	K_3_PO_4_	DMF	27%
8	Cu(OAc)_2’_H_2_O	K_2_CO_3_	DMF	70%
9	Cu(CF_3_COO)_2_	K_2_CO_3_	DMF	Trace
10	CuF_2_	K_2_CO_3_	DMF	17%
11	CuI	K_2_CO_3_	DMF	6%
12	Cu(OAc)_2_	K_2_CO_3_	DMSO	68%
13	Cu(OAc)_2_	K_2_CO_3_	iPrOH	n.r.
14	Cu(OAc)_2_	K_2_CO_3_	DCE	n.r.
15^c^	Cu(OAc)_2_	K_2_CO_3_	DMF	76%
16^d^	Cu(OAc)_2_	K_2_CO_3_	DMF	24%
17^e^	Cu(OAc)_2_	K_2_CO_3_	DMF	38%
**18^f^**	**Cu(OAc)_2_**	**K_2_CO_3_**	**DMF**	**81%**
19^f,g^	Cu(OAc)_2_	K_2_CO_3_	DMF	75%

^a^Reaction conditions: **1a** (0.4 mmol), **2a** (0.8 mmol), copper salts (0.4 mmol), bases (0.8 mmol), DMF (2 mL), 90 °C ,12 h, O_2_; ^b^yield of isolated product. ^c^24 h; ^d^sealed tube without oxygen; ^e^copper salts (0.08 mmol); ^f^80 °C; ^g^air, 18 h.

**Scheme 2:**
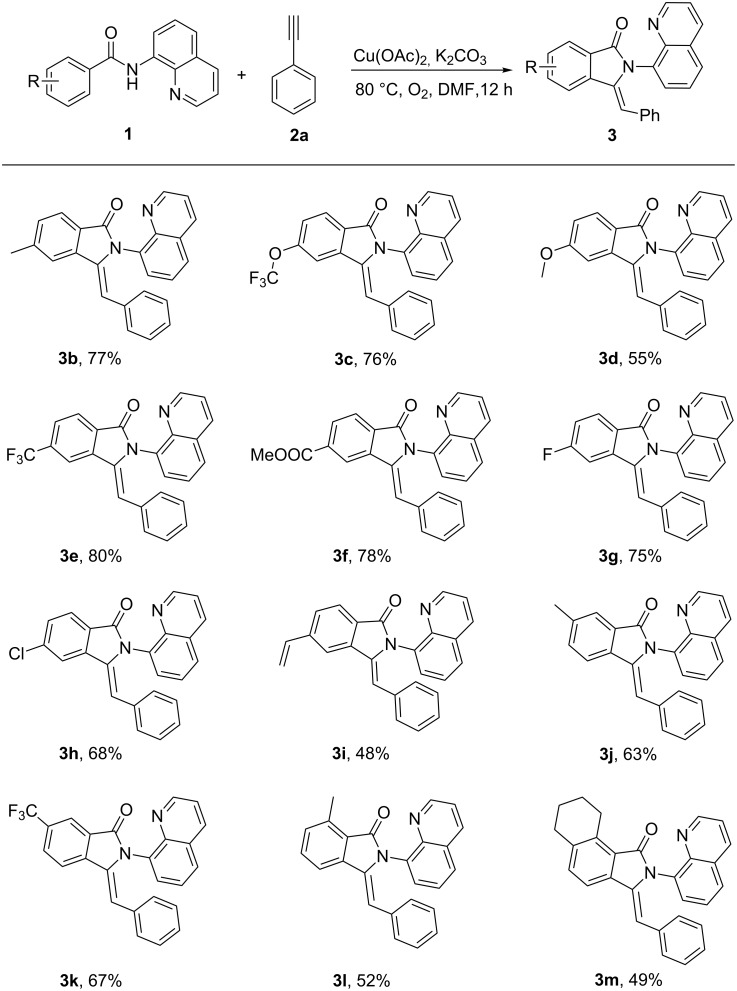
Copper-mediated reaction of ethynylbenzene with carboxylic acid derivatives. Reaction conditions: **1** (0.4 mmol), **2a** (0.8 mmol), Cu(OAc)_2_ (0.4 mmol), K_2_CO_3_ (0.8 mmol), DMF (2 mL), 80 °C , 12 h, O_2_, isolated yield.

Next, the scope of 8-aminoquinoline benzamides and the generality of this process were investigated under the optimized conditions. As shown in Scheme 2, most of the examined substrates (**1b**–**1m**) provided the corresponding products (**3b**–**3m**) in moderate to good yields. Benzamides with weak electron-donating groups (–Me, –OCF_3,_) at the para-position were well compatible with good yields (**3b**, **3c**), while the introduction of a methoxy group led to a slight decrease in the yield (**3d**). Electron-poor benzamides (–CF_3_, –COOMe) worked well under the transformation system and gave good yields (**3e**, **3f**). Notably, halides and an ethenyl group were tolerated under the standard reaction conditions (**3g**–**3i**), which could undergo further elaboration. The C–H activation of meta-substituted benzamides occurred predominantly at less sterically congested sites (**3j**, **3k**). A ortho-substituted and a tetrahydronaphthalene derivative worked well, respectively, and provided moderate yields (**3l**, **3m**).

**Scheme 3:**
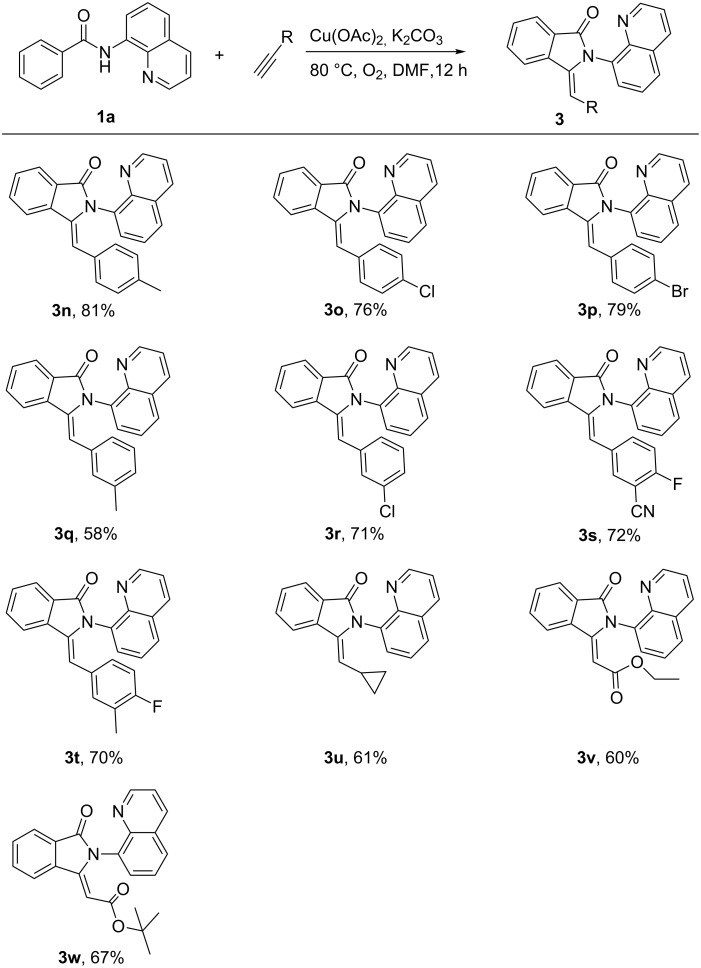
Copper-mediated reaction of *N*-(quinolin-8-yl)benzamide with terminal alkynes. Reaction conditions: **1a** (0.4 mmol), **2** (0.8 mmol), Cu(OAc)_2_ (0.4 mmol), K_2_CO_3_ (0.8 mmol), DMF (2 mL), 80 °C ,12 h,O_2_; isolated yield.

We also tested a variety of terminal alkynes as coupling partners with *N*-(quinolin-8-yl)benzamides (Scheme 3). Both electron-rich (–Me) and electron-poor (–CN, –F) ethynylbenzene proceeded smoothly under this transformation system with good yields (**3n–3t**). The halogeno- and cyano-substituted ethynylbenzenes exhibited good tolerance under the reaction conditions (**3o**, **3p**, **3r–3t**). Apart from arylalkynes, alkylacetylene and propiolate derivatives were also compatible in the transformation and gave moderate yields (**3u–3w**).

**Scheme 4:**
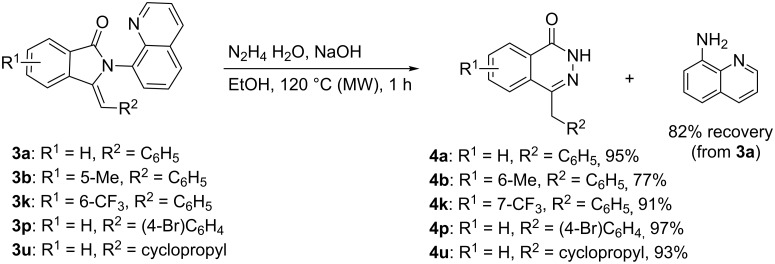
Hydrazinolysis and removal of the directing group.

Finally, the obtained products **3** could be smoothly transformed to 4-benzylphthalazin-1(2*H*)-ones **4** in excellent yields under hydrazinolysis conditions. The directing group quinolin-8-amine was easily removed and recycled by treatment of **3a** with hydrazine hydrate and NaOH in EtOH at 120 °C under microwave irradiation (Scheme 4). Both electron-rich and electron-deficient 4-benzylphthalazin-1(2*H*)-one derivatives were obtained in good yields (**4b**, **4k**). The halo-substituted phenyl moiety was well tolerated under the basic conditions (**4p**). Alkyl derivative **3u** was compatible in the transformation and gave an excellent yield (**4u**). It was worth mentioning that the simplified process was achieved without silica gel column purification and the directing moiety quinolin-8-amine could be recovered in good yield.

**Scheme 5:**
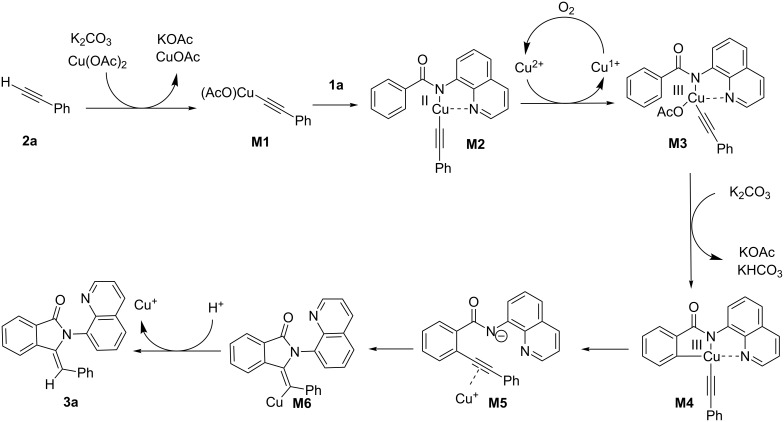
Plausible reaction mechanism.

Based on previous works [22–23,25], a copper(II)-mediated C–H functionalization pathway is proposed in Scheme 5. A base-promoted cupration of the relatively acidic C–H of ethynylbenzene provides ethynylcopper intermediate **M1.** The following bidentate chelation with **1a** yields organocopper(II) complex **M2**, which undergoes Cu(OAc)_2_-promoted oxidation and intramolecular C–H cupration to deliver chelated organocopper(III) intermediate **M4**. The corresponding product **3a** is formed by the subsequent reductive elimination and intramolecular annulation.

## Conclusion

In conclusion, we have developed a novel strategy for the construction of the phthalazin-1(2H)-one scaffold by means of the copper-mediated cascade C–H/C–H coupling and intramolecular annulations and a subsequent facile hydrazinolysis. This C–H activation transformation proceeds smoothly for the construction of the isoindolin-1-one scaffold under mild conditions with less equivalents of copper salts and a lower temperature (80 °C), and exhibits wide generality, good functional tolerance and high stereo- and regioselectivity. With the removal of the directing group, the resulting moiety could be easily transformed into the phthalazin-1(2*H*)-one scaffold by treatment with hydrazine hydrate and sodium hydroxide. As the phthalazin-1(2*H*)-one scaffold is identified as a privilege moiety and bioactive nucleus in pharmaceuticals, this modified procedure will be of importance to medicinal chemists.

## Experimental

**General procedure for the synthesis of amides 1** [20,22–23,26]: Synthesis of **1a** is representative. 8-Aminoquinoline (1.0 g, 6.94 mmol) and Et_3_N (1.2 mL, 8.32 mmol) was added into dichloromethane (25 mL). The resulting solution was cooled in an ice bath and then benzoyl chloride (0.95 mL, 8.32 mmol) was added drop-wise. The resulting mixture was stirred at room temperature for 12 h. The mixture was quenched with water and extracted with dichloromethane (3 times). The combined organic layer was dried with Na_2_SO_4_, concentrated, and purified by column chromatography on silica gel (PE/DCM 4:1–1:1) to give *N*-(quinolin-8-yl)benzamide (**1a**) as a white solid.

**Copper-mediated coupling of benzamide 1 and alkynyl substrate 2:** The reaction of benzamide **1a** with ethynylbenzene (**2a**) is representative. The dry sealed tube was charged with *N*-(quinolin-8-yl)benzamide (**1a**, 99 mg, 0.4 mmol), ethynylbenzene (**2a**, 82 mg, 0.8 mmol), Cu(OAc)_2_ (73 mg, 0.4 mmol), K_2_CO_3_ (111 mg, 0.8 mmol) and 2 mL DMF. The resulting mixture was stirred at 80 °C for 12 hours under oxygen atmosphere (O_2_ balloon). The mixture was diluted with dichloromethane (10 mL), filtered through a celite pad, and washed with dichloromethane (20 mL). The resulting mixture was washed with water (3 times) and brine. The combined organic layer was dried with Na_2_SO_4_, concentrated, and purified by column chromatography on silica gel (DCM/MeOH 200:1–50:1) to give (*Z*)-3-benzylidene-2-(quinolin-8-yl)isoindolin-1-one (**3a**) as a pale yellow solid (81%).

**General procedure for the synthesis of phthalazin-1(2*H*)-one 4 and recovery of quinolin-8-amine (DG):** The synthesis of **4a** is representative. The microwave tube was charged with (*Z*)-3-benzylidene-2-(quinolin-8-yl)isoindolin-1-one (**3a**, 50 mg, 0.144 mmol), hydrazine hydrate (75 µL, 1.44 mmol), sodium hydroxide (57 mg, 1.44 mmol) and 2 mL EtOH. The resulting mixture was stirred at 120 °C for 1 h under microwave irradiation. The solution was diluted with dichloromethane (30 mL) and washed with 1 M HCl (20 mL, three times). The combined organic layer was washed with NaHCO_3_ aqueous solution (15 mL) and brine (15 mL). The resulting solution was dried with Na_2_SO_4_ and concentrated to give 4-benzylphthalazin-1(2*H*)-one (**4a**) in 95% yield. The former acidic aqueous layer was neutralized with NaHCO_3_ aqueous solution and extracted with dichloromethane (15 mL, three times). The combined organic layer was washed with brine (15 mL), dried with Na_2_SO_4_ and concentrated to give quinolin-8-amine (DG, 82%).

## Supporting Information

File 1 General information, experimental details, characterization data and copies of ^1^H and ^13^C NMR spectra.
