# Supplementary material for: The facile construction of the phthalazin-1(2H)-one scaffold via copper-mediated C–H(sp2)/C–H(sp) coupling under mild conditions
Source: Beilstein J Org Chem. 2015 Sep 14;11:1624–31. doi: 10.3762/bjoc.11.177 (PMC4660935; doi:10.3762/bjoc.11.177)
Supplement: File 1 — General information, experimental details, characterization data and copies of 1H and 13C NMR spectra. [file Beilstein_J_Org_Chem-11-1624-s001.pdf]

Supporting Information

for

The facile construction of the phthalazin-1(2H)-one scaffold via copper-mediated C–H(sp^2)/C–H(sp) coupling under mild conditions

Wei Zhu, Bao Wang, Shengbin Zhou, Hong Liu*

CAS Key Laboratory of Receptor Research, Shanghai Institute of Materia Medica, Chinese Academy of Sciences, 555 Zuchongzhi Road, Shanghai 201203, P. R. China.

Email: Hong Liu* - hliu@mail.shcnc.ac.cn
*Corresponding author

General information, experimental details, characterization data and copies of ^{1}H and ^{13}C NMR spectra

Table of contents

(A) General methods..S2

(B) X-ray diffraction of 3a...S3

(C) Analytical characterization data of products..S4

(D) Copies of ^{1}H NMR and ^{13}C NMR spectra for the products..................... S23

(A) General methods

The reagents (chemicals) were purchased from commercial sources, and used without further purification. Analytical thin layer chromatography (TLC) was HSGF 254 (0.15–0.2 mm thickness). All products were characterized by their NMR and MS spectra. ^{1}H and ^{13}C NMR spectra were recorded in dimethyl sulfoxide-d_6 (DMSO-d_6) on a 400 MHz or 500 MHz instrument. Chemical shifts were reported in parts per million (ppm, δ) downfield from tetramethylsilane. Proton coupling patterns are described as singlet (s), doublet (d), triplet (t), quartet (q), multiplet (m), and broad (br). High-resolution mass spectra (HRMS) were measured on a Micromass Ultra Q-TOF spectrometer.

(B) The absolute configuration of of 3a

X-ray single crystal structure analysis of (*Z*)**-3a**

X-ray crystallographic data of (*Z*)**-3a** were solutions at *T* = 296(2) K: $C_{24}H_{16}N_2O$, Mr = 348.39, orthorhombic. Space group *Pbca*, *a* = 14.690(4) Å, *b* = 8.800(2) Å, *c* = 27.615(6) Å, α = 90°, β = 90°, γ = 90°, *V* =3569.8(14) Å^3, *Z* = 8.

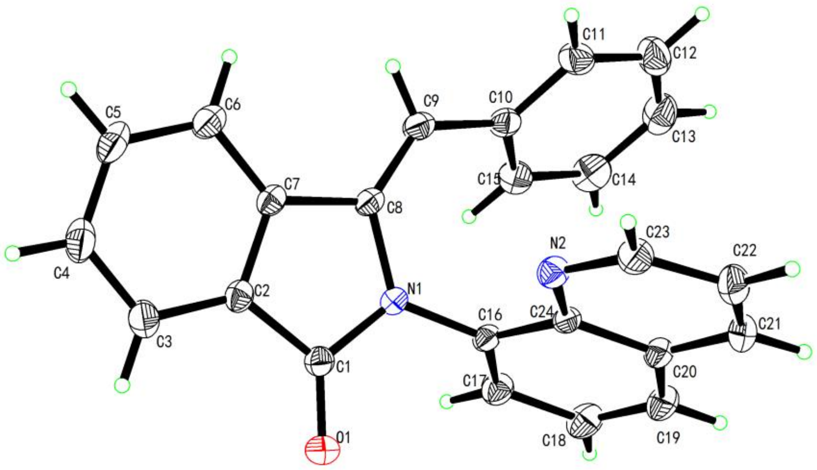

Figure S1. (*Z*)-3-benzylidene-2-(quinolin-8-yl)isoindolin-1-one (**3a**) by X-ray analysis.

These data can be obtained free of charge from the Cambridge Crystallographic Data Centre *via* www.ccdc.cam.ac.uk/data_request/cif, the CCDC number is 1062214.

(C) Analytical characterization data of products

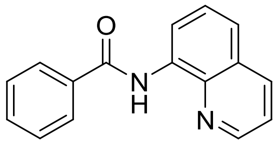

***N*-(Quinolin-8-yl)benzamide (1a)**

^{1}H NMR (400 MHz, DMSO) δ 10.72 (s, 1H), 9.03 (dd, *J* = 4.2, 1.6 Hz, 1H), 8.80 (dd, *J* = 7.6, 1.2 Hz, 1H), 8.51 (dd, *J* = 8.3, 1.6 Hz, 1H), 8.15 – 8.06 (m, 2H), 7.80 (dd, *J* = 8.3, 1.2 Hz, 1H), 7.70 (m, 5H). LRMS (ESI) $[M+H]^+$ found m/z 249.1.

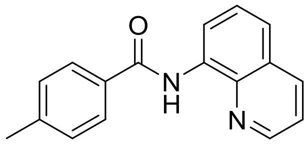

4-Methyl-*N*-(quinolin-8-yl)benzamide (1b)

^{1}H NMR (400 MHz, DMSO) δ 10.68 (s, 1H), 9.03 (dd, *J* = 4.2, 1.6 Hz, 1H), 8.79 (dd, *J* = 7.6, 1.1 Hz, 1H), 8.51 (dd, *J* = 8.3, 1.5 Hz, 1H), 7.99 (d, *J* = 8.1 Hz, 2H), 7.78 (dd, *J* = 8.2, 1.1 Hz, 1H), 7.75 – 7.67 (m, 2H), 7.47 (d, *J* = 8.0 Hz, 2H), 2.46 (s, 3H). LRMS (ESI) $[M+H]^+$ found m/z 263.1.

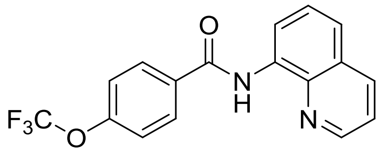

***N*-(Quinolin-8-yl)-4-(trifluoromethoxy)benzamide (1c)**

^{1}H NMR (400 MHz, DMSO) δ 10.67 (s, 1H), 8.97 (dd, *J* = 4.2, 1.7 Hz, 1H), 8.69 (dd, *J* = 7.6, 1.3 Hz, 1H), 8.46 (dd, *J* = 8.3, 1.6 Hz, 1H), 8.19 – 8.14 (m, 2H), 7.76 (dd, *J* = 8.3, 1.3 Hz, 1H), 7.69 – 7.63 (m, 2H), 7.60 (dd, *J* = 8.8, 0.9 Hz, 2H). LRMS (ESI) $[M+H]^+$ found m/z 333.1.

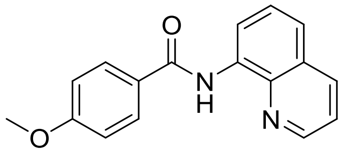

4-Methoxy-*N*-(quinolin-8-yl)benzamide (1d)

^{1}H NMR (400 MHz, DMSO) δ 10.65 (s, 1H), 9.04 (dd, *J* = 4.2, 1.6 Hz, 1H), 8.78 (dd, *J* = 7.6, 1.1 Hz, 1H), 8.52 (dd, *J* = 8.3, 1.5 Hz, 1H), 8.07 (d, *J* = 8.8 Hz, 2H), 7.78 (dd, *J* = 8.3, 1.1 Hz, 1H), 7.76 – 7.68 (m, 2H), 7.22 (d, *J* = 8.8 Hz, 2H), 3.93 (s, 3H). LRMS (ESI) $[M+H]^+$ found m/z 279.1.

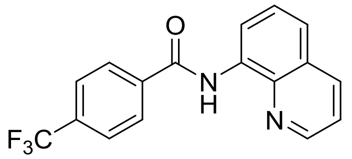

***N*-(Quinolin-8-yl)-4-(trifluoromethyl)benzamide (1e)**

^{1}H NMR (400 MHz, DMSO) δ 10.78 (s, 1H), 9.02 (dd, *J* = 4.2, 1.6 Hz, 1H), 8.75 (d, *J* = 7.6 Hz, 1H), 8.50 (dd, *J* = 8.3, 1.5 Hz, 1H), 8.26 (d, *J* = 8.2 Hz, 2H), 8.01 (d, *J* = 8.3 Hz, 2H), 7.82 – 7.76 (m, 1H), 7.71 (m, 2H). LRMS (ESI) $[M+H]^+$ found m/z 317.1.

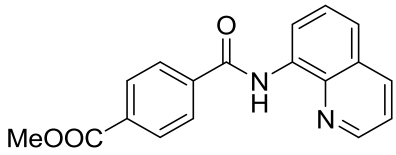

Methyl 4-(quinolin-8-ylcarbamoyl)benzoate (1f)

^{1}H NMR (400 MHz, DMSO) δ 10.70 (s, 1H), 8.97 (dd, *J* = 4.2, 1.5 Hz, 1H), 8.70 (d, *J* = 7.6 Hz, 1H), 8.45 (d, *J* = 8.3 Hz, 1H), 8.14 (s, 4H), 7.76 (d, *J* = 8.3 Hz, 1H), 7.69 – 7.59 (m, 2H), 3.90 (s, 3H). LRMS (ESI) $[M+H]^+$ found m/z 307.1.

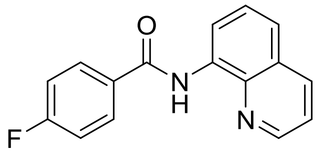

4-Fluoro-*N*-(quinolin-8-yl)benzamide (1g)

^{1}H NMR (400 MHz, DMSO) δ 10.60 (s, 1H), 8.96 (dd, *J* = 4.1, 1.3 Hz, 1H), 8.69 (d, *J* = 7.6 Hz, 1H), 8.43 (d, *J* = 8.3 Hz, 1H), 8.09 (dd, *J* = 8.6, 5.4 Hz, 2H), 7.73 (d, *J* = 8.2 Hz, 1H), 7.65 (m, 2H), 7.43 (t, *J* = 8.8 Hz, 2H). LRMS (ESI) $[M+H]^+$ found m/z 267.1.

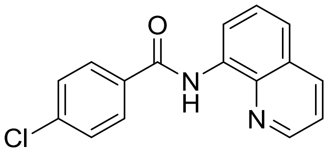

4-Chloro-*N*-(quinolin-8-yl)benzamide (1h)

^{1}H NMR (400 MHz, DMSO) δ 10.71 (s, 1H), 9.03 (d, *J* = 4.2 Hz, 1H), 8.75 (d, *J* = 7.6 Hz, 1H), 8.51 (d, *J* = 8.1 Hz, 1H), 8.10 (d, *J* = 8.5 Hz, 2H), 7.81 (d, *J* = 8.1 Hz, 1H), 7.72 (m, 4H). LRMS (ESI) $[M+H]^+$ found m/z 283.1.

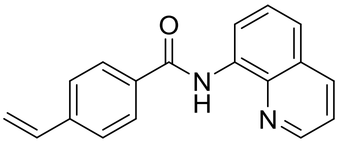

***N*-(Quinolin-8-yl)-4-vinylbenzamide (1i)**

^{1}H NMR (400 MHz, DMSO) δ 10.67 (s, 1H), 8.99 (dd, *J* = 4.2, 1.7 Hz, 1H), 8.74 (dd, *J* = 7.6, 1.3 Hz, 1H), 8.47 (dd, *J* = 8.3, 1.6 Hz, 1H), 8.03 (d, *J* = 8.4 Hz, 2H), 7.77 – 7.64 (m, 5H), 6.87 (dd, *J* = 17.7, 11.0 Hz, 1H), 6.03 (dd, *J* = 17.7, 0.6 Hz, 1H), 5.45 (dd, *J* = 11.0, 0.6 Hz, 1H). LRMS (ESI) $[M+H]^+$ found m/z 275.1.

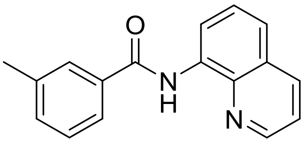

3-Methyl-*N*-(quinolin-8-yl)benzamide (1j)

^{1}H NMR (400 MHz, DMSO) δ 10.65 (s, 1H), 9.02 (dd, *J* = 4.2, 1.5 Hz, 1H), 8.79 (dd, *J* = 7.5, 0.9 Hz, 1H), 8.49 (dd, *J* = 8.3, 1.5 Hz, 1H), 7.87 (d, *J* = 9.0 Hz, 2H), 7.77 (dd, *J* = 8.2, 0.9 Hz, 1H), 7.70 (dt, *J* = 13.6, 6.3 Hz, 2H), 7.58 – 7.49 (m, 2H), 2.48 (s, 3H). LRMS (ESI) $[M+H]^+$ found m/z 263.1.

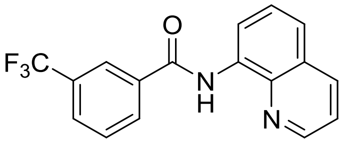

***N*-(Quinolin-8-yl)-3-(trifluoromethyl)benzamide (1k)**

^{1}H NMR (400 MHz, DMSO) δ 10.81 (s, 1H), 9.09 – 8.95 (m, 1H), 8.71 (d, *J* = 7.6 Hz,

1H), 8.52 (d, J = 8.3 Hz, 1H), 8.39 (d, J = 7.4 Hz, 2H), 8.09 (d, J = 8.1 Hz, 1H), 7.92 (t, J = 7.9 Hz, 1H), 7.84 (d, J = 8.3 Hz, 1H), 7.77 – 7.69 (m, 2H). LRMS (ESI) $[M+H]^+$ found m/z 317.1.

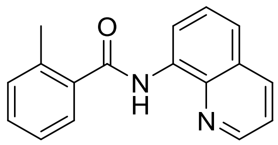

2-Methyl-*N*-(quinolin-8-yl)benzamide (1l)

^{1}H NMR (400 MHz, DMSO) δ 10.21 (s, 1H), 8.92 (dd, J = 4.2, 1.5 Hz, 1H), 8.77 (d, J = 7.5 Hz, 1H), 8.46 (dd, J = 8.3, 1.5 Hz, 1H), 7.76 (d, J = 7.3 Hz, 1H), 7.72 – 7.64 (m, 3H), 7.48 (t, J = 6.9 Hz, 1H), 7.39 (t, J = 6.8 Hz, 2H), 2.52 (s, 3H). LRMS (ESI) $[M+H]^+$ found m/z 263.1.

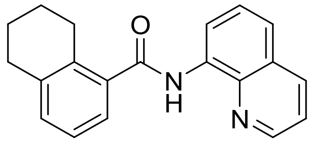

***N*-(Quinolin-8-yl)-5,6,7,8-tetrahydronaphthalene-1-carboxamide (1m)**

^{1}H NMR (400 MHz, DMSO) δ 10.08 (s, 1H), 8.87 (dd, J = 4.2, 1.6 Hz, 1H), 8.73 (d, J = 7.5 Hz, 1H), 8.43 (dd, J = 8.3, 1.6 Hz, 1H), 7.72 (dd, J = 8.3, 1.3 Hz, 1H), 7.66 – 7.60 (m, 2H), 7.44 – 7.40 (m, 1H), 7.27 – 7.21 (m, 2H), 2.89 (t, J = 5.4 Hz, 2H), 2.79 (t, J = 5.3 Hz, 2H), 1.75 – 1.70 (m, 4H). LRMS (ESI) $[M+H]^+$ found m/z 303.1.

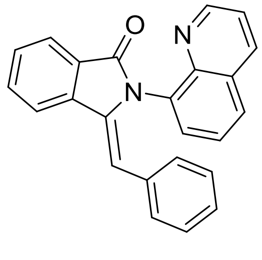

(*Z*)-3-Benzylidene-2-(quinolin-8-yl)isoindolin-1-one (3a)

Obtained as a pale yellow solid by column chromatography (DCM/MeOH = 200/1 to 50/1), yield 81%. ^{1}H NMR (400 MHz, DMSO)

δ 8.76 (dd, J = 4.1, 1.6 Hz, 1H), 8.24 – 8.18 (m, 2H), 7.88 (d, J = 7.5 Hz, 1H), 7.85 –

7.76 (m, 2H), 7.67 (t, *J* = 7.5 Hz, 1H), 7.62 (dd, *J* = 7.3, 1.1 Hz, 1H), 7.47 – 7.38 (m, 2H), 7.03 (s, 1H), 6.70 (t, *J* = 6.5 Hz, 1H), 6.58 – 6.50 (m, 4H). ^{13}C NMR (126 MHz, DMSO) δ 167.20, 150.22, 143.71, 138.46, 135.94, 135.47, 133.78, 133.19, 132.69, 130.42, 129.39, 128.50, 128.30, 127.92, 127.45, 126.15, 125.92, 125.61, 123.01, 121.52, 120.42, 107.80. LRMS (ESI) $[M+H]^+$ found m/z 349.1. HRMS (ESI) $[M+H]^+$ found m/z 349.1330, calcd for $C_{24}H_{17}N_2O$ 349.1335.

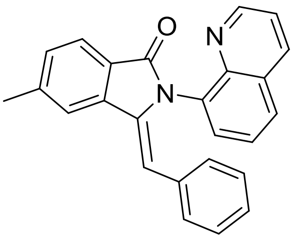

(*Z*)-3-Benzylidene-5-methyl-2-(quinolin-8-yl)isoindolin-1-one (3b)

Obtained as a white solid by column chromatography (DCM/MeOH = 200/1 to 50/1), yield 77%. ^{1}H NMR (400 MHz, DMSO) δ 8.76 (d, *J* = 2.6 Hz, 1H), 8.20 (d, *J* = 8.3 Hz, 1H), 8.00 (s, 1H), 7.76 (d, *J* = 7.8 Hz, 2H), 7.59 (d, *J* = 7.1 Hz, 1H), 7.43 (m, 3H), 6.96 (s, 1H), 6.70 (t, *J* = 6.5 Hz, 1H), 6.57 – 6.49 (m, 4H), 2.54 (s, 3H). ^{13}C NMR (126 MHz, DMSO) δ 167.26, 150.17, 143.76, 143.06, 138.85, 135.91, 135.55, 133.92, 133.28, 130.37, 130.32, 128.41, 128.30, 127.92, 126.15, 125.86, 125.59, 125.12, 122.86, 121.48, 120.55, 107.29, 21.67. LRMS (ESI) $[M+H]^+$ found m/z 363.1. HRMS (ESI) $[M+H]^+$ found m/z 363.1487, calcd for $C_{25}H_{19}N_2O$ 363.1492.

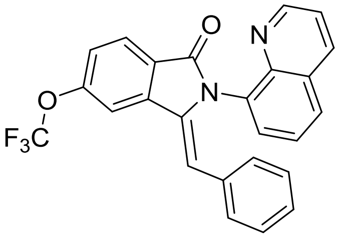

(*Z*)-3-Benzylidene-2-(quinolin-8-yl)-5-(trifluoromethoxy)isoindolin-1-one (3c)

Obtained as a white solid by column chromatography (DCM/MeOH = 200/1 to 50/1), yield 76%.^{1}H NMR (400 MHz, DMSO) δ 8.76 (dd, *J* = 4.1, 1.7 Hz, 1H), 8.32 (s, 1H),

8.22 (dd, *J* = 8.3, 1.6 Hz, 1H), 8.02 (d, *J* = 8.3 Hz, 1H), 7.79 (dd, *J* = 8.3, 1.2 Hz, 1H), 7.65 (m, 2H), 7.44 (m, 2H), 7.21 (s, 1H), 6.71 (m, 1H), 6.55 (m, 4H). ^{13}C NMR (126 MHz, DMSO) δ 165.94, 151.67, 150.32, 143.55, 140.52, 135.99, 134.40, 133.43, 132.82, 130.44, 128.71, 128.31, 127.86, 126.30, 126.21, 125.65, 125.42, 122.40, 121.60, 121.11, 119.06, 113.61, 109.94. LRMS (ESI) $[M+H]^+$ found m/z 433.1. HRMS (ESI) $[M+H]^+$ found m/z 433.1167, calcd for $C_{25}H_{16}F_3N_2O_2$ 433.1158.

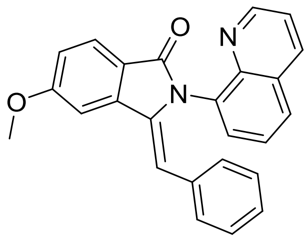

(*Z*)-3-Benzylidene-5-methoxy-2-(quinolin-8-yl)isoindolin-1-one (3d)

Obtained as a white solid by column chromatography (DCM/MeOH = 200/1 to 50/1), yield 55%. ^{1}H NMR (500 MHz, DMSO) δ 8.76 (dd, *J* = 4.1, 1.5 Hz, 1H), 8.20 (d, *J* = 8.1 Hz, 1H), 7.78-7,75 (3H), 7.62 – 7.56 (m, 1H), 7.45 – 7.38 (m, 2H), 7.18 (dd, *J* = 8.4, 2.0 Hz, 1H), 7.05 (s, 1H), 6.69 (t, *J* = 6.8 Hz, 1H), 6.58 – 6.50 (m, 4H), 3.97 (s, 3H). ^{13}C NMR (126 MHz, DMSO) δ 167.02, 163.41, 150.14, 143.77, 140.98, 135.93, 135.56, 133.99, 133.30, 130.41, 128.35, 128.31, 127.89, 126.16, 125.88, 125.61, 124.51, 121.47, 120.27, 117.08, 107.66, 104.32, 56.03.LRMS (ESI) $[M+H]^+$ found m/z 379.1. HRMS (ESI) $[M+H]^+$ found m/z 379.1432, calcd for $C_{25}H_{19}N_2O_2$ 379.1441.

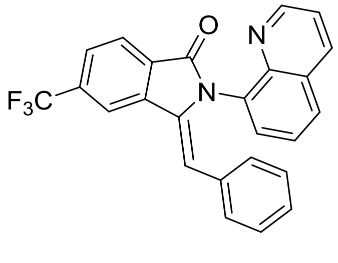

(*Z*)-3-Benzylidene-2-(quinolin-8-yl)-5-(trifluoromethyl)isoindolin-1-one (3e)

Obtained as a white solid by column chromatography (DCM/MeOH = 200/1 to 50/1), yield 80%. ^{1}H NMR (400 MHz, DMSO) δ 8.76 (dd, *J* = 4.2, 1.7 Hz, 1H), 8.72 (s, 1H), 8.23 (dd, *J* = 8.3, 1.7 Hz, 1H), 8.10 (d, *J* = 8.0 Hz, 1H), 8.00 (d, *J* = 8.0 Hz, 1H), 7.81

(dd, *J* = 8.3, 1.3 Hz, 1H), 7.67 (dd, *J* = 7.3, 1.3 Hz, 1H), 7.48 – 7.39 (m, 2H), 7.34 (s, 1H), 6.72 (t, *J* = 6.9 Hz, 1H), 6.59 – 6.50 (m, 4H). ^{13}C NMR (151 MHz, DMSO) δ 165.97, 150.39, 143.52, 138.95, 136.02, 134.41, 133.36, 132.84, 132.80 (q, 31.7 Hz), 130.49, 130.41, 128.81, 128.33, 127.88, 126.25, 126.02(q, 3.6 Hz), 125.67, 124.95, 124.25, 123.14, 121.64, 118.22 (q, 3.6 Hz), 110.37.

LRMS (ESI) $[M+H]^+$ found m/z 417.1. HRMS (ESI) $[M+H]^+$ found m/z 417.1209, calcd for $C_{25}H_{16}F_3N_2O$ 417.1209.

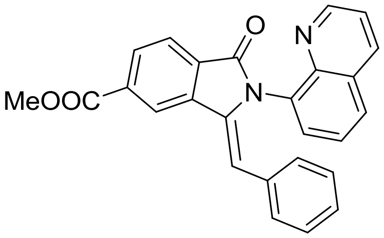

(*Z*)-Methyl 3-benzylidene-1-oxo-2-(quinolin-8-yl)isoindoline-5-carboxylate (3f)

Obtained as a yellow solid by column chromatography (DCM/MeOH = 200/1 to 50/1), yield 78%. ^{1}H NMR (400 MHz, DMSO) δ 8.77 (dd, *J* = 3.5, 2.1 Hz, 2H), 8.26 – 8.20 (m, 2H), 8.02 (d, *J* = 7.9 Hz, 1H), 7.80 (dd, *J* = 8.2, 1.1 Hz, 1H), 7.65 (dd, *J* = 7.3, 1.2 Hz, 1H), 7.49 – 7.39 (m, 2H), 7.27 (s, 1H), 6.72 (m, 1H), 6.59 – 6.52 (m, 4H), 3.97 (s, 3H). ^{13}C NMR (126 MHz, DMSO) δ 166.21, 165.79, 150.35, 143.56, 138.57, 135.99, 134.62, 133.51, 133.49, 132.98, 130.84, 130.34, 129.92, 128.72, 128.31, 127.95, 126.18, 126.14, 125.64, 123.52, 121.60, 121.47, 109.58, 52.68. LRMS (ESI) $[M+H]^+$ found m/z 407.1. HRMS (ESI) $[M+H]^+$ found m/z 407.1399, calcd for $C_{26}H_{19}N_2O_3$ 407.1390.

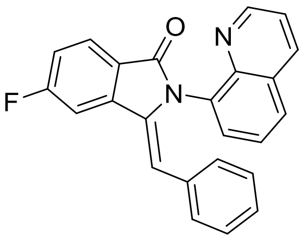

(*Z*)-3-Benzylidene-5-fluoro-2-(quinolin-8-yl)isoindolin-1-one (3g)

Obtained as a pale yellow solid by column chromatography (DCM/MeOH = 200/1 to

50/1), yield 75%. ^{1}H NMR (400 MHz, DMSO) δ 8.77 (d, *J* = 2.7 Hz, 1H), 8.21 (d, *J* = 8.2 Hz, 1H), 8.13 (d, *J* = 9.1 Hz, 1H), 7.94 (dd, *J* = 8.3, 5.1 Hz, 1H), 7.78 (d, *J* = 8.1 Hz, 1H), 7.63 (d, *J* = 7.2 Hz, 1H), 7.53 – 7.38 (m, 3H), 7.10 (s, 1H), 6.71 (t, *J* = 6.8 Hz, 1H), 6.60 – 6.48 (m, 4H). ^{13}C NMR (126 MHz, DMSO) δ 166.29, 166.23, 164.32, 162.31, 150.28, 143.63, δ 141.10 (d, *J* = 11.0 Hz), 135.97, 134.69 (d, *J* = 3.3 Hz), 133.58, 132.89, 131.63, 130.42, 128.82, 128.61, 128.31, 127.86, 126.21, 126.12, 125.70, 125.63, 123.89, 121.56, 117.13 (d, *J* = 24.2 Hz), 109.18, 107.63 (d, *J* = 25.3 Hz). LRMS (ESI) $[M+H]^+$ found m/z 367.0. HRMS (ESI) $[M+H]^+$ found m/z 367.1237, calcd for $C_{24}H_{16}FN_2O$ 367.1241.

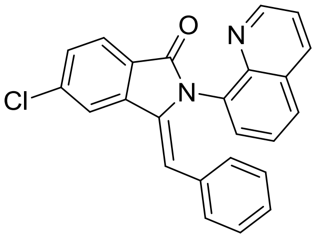

(*Z*)-3-Benzylidene-5-chloro-2-(quinolin-8-yl)isoindolin-1-one (3h)

Obtained as a white solid by column chromatography (DCM/MeOH = 200/1 to 50/1), yield 68%. ^{1}H NMR (400 MHz, DMSO) δ 8.76 (dd, *J* = 4.1, 1.6 Hz, 1H), 8.39 (d, *J* = 1.5 Hz, 1H), 8.21 (dd, *J* = 8.3, 1.6 Hz, 1H), 7.89 (d, *J* = 8.1 Hz, 1H), 7.79 (dd, *J* = 8.2, 1.1 Hz, 1H), 7.70 (dd, *J* = 8.1, 1.7 Hz, 1H), 7.63 (dd, *J* = 7.3, 1.2 Hz, 1H), 7.48 – 7.39 (m, 2H), 7.15 (s, 1H), 6.71 (t, *J* = 7.1 Hz, 1H), 6.59 – 6.50 (m, 4H).^{13}C NMR (126 MHz, DMSO) δ 166.24, 150.32, 143.59, 140.25, 137.80, 135.99, 134.37, 133.49, 132.91, 130.41, 129.53, 128.67, 128.31, 127.88, 126.23, 126.16, 126.11, 125.65, 124.86, 121.59, 120.73, 109.52. LRMS (ESI) $[M+H]^+$ found m/z 383.0. HRMS (ESI) $[M+H]^+$ found m/z 383.0949, calcd for $C_{24}H_{16}ClN_2O$ 383.0946.

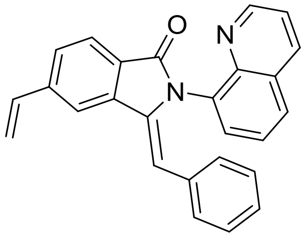

(*Z*)-3-Benzylidene-2-(quinolin-8-yl)-5-vinylisoindolin-1-one (3i)

Obtained as a pale yellow solid by column chromatography (DCM/MeOH = 200/1 to 50/1), yield 48%. ^{1}H NMR (400 MHz, DMSO) δ 8.76 (dd, *J* = 4.1, 1.6 Hz, 1H), 8.36 (s, 1H), 8.21 (dd, *J* = 8.3, 1.5 Hz, 1H), 7.84 (d, *J* = 7.9 Hz, 1H), 7.77 (dd, *J* = 13.8, 5.0 Hz, 2H), 7.62 (dd, *J* = 7.3, 1.1 Hz, 1H), 7.48 – 7.33 (m, 2H), 7.09 (s, 1H), 6.97 (dd, *J* = 17.7, 11.0 Hz, 1H), 6.70 (m, 1H), 6.60 – 6.48 (m, 4H), 6.19 (d, *J* = 17.6 Hz, 1H), 5.51 (d, *J* = 11.1 Hz, 1H). ^{13}C NMR (126 MHz, DMSO) δ 166.95, 150.21, 143.69, 141.60, 139.06, 136.19, 135.93, 135.43, 133.82, 133.20, 130.36, 128.48, 128.31, 127.89, 127.55, 126.74, 126.18, 125.93, 125.61, 123.31, 121.51, 117.80, 117.32, 107.85. LRMS (ESI) $[M+H]^+$ found m/z 375.1. HRMS (ESI) $[M+H]^+$ found m/z 375.1492, calcd for $C_{26}H_{19}N_2O$ 375.1492.

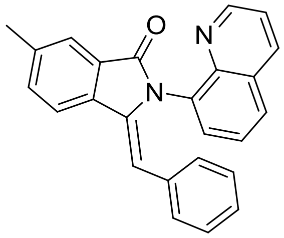

(*Z*)-3-Benzylidene-6-methyl-2-(quinolin-8-yl)isoindolin-1-one (3j)

Obtained as a pale yellow solid by column chromatography (DCM/MeOH = 200/1 to 50/1), yield 63%. ^{1}H NMR (400 MHz, DMSO) δ 8.76 (dd, *J* = 4.1, 1.6 Hz, 1H), 8.21 (dd, *J* = 8.3, 1.5 Hz, 1H), 8.07 (d, *J* = 7.9 Hz, 1H), 7.77 (dd, *J* = 8.2, 1.2 Hz, 1H), 7.69 (s, 1H), 7.65 – 7.58 (m, 2H), 7.46 – 7.37 (m, 2H), 6.95 (s, 1H), 6.71 – 6.65 (m, 1H), 6.57 – 6.49 (m, 4H), 2.51 (s, 4H). ^{13}C NMR (126 MHz, DMSO) δ 167.32, 150.20, 143.76, 139.34, 136.08, 135.93, 135.55, 133.90, 133.61, 133.32, 130.40, 128.45, 128.30, 127.95, 127.70, 126.14, 125.82, 125.60, 122.98, 121.50, 120.24, 107.05, 21.09. LRMS (ESI) $[M+H]^+$ found m/z 363.1. HRMS (ESI) $[M+H]^+$ found m/z 363.1502, calcd for $C_{25}H_{19}N_2O$ 363.1492.

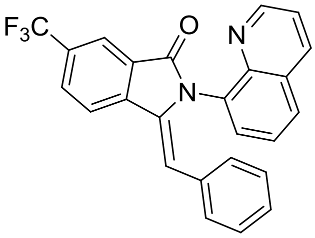

(*Z*)-3-Benzylidene-2-(quinolin-8-yl)-6-(trifluoromethyl)isoindolin-1-one (3k)

Obtained as a pale yellow solid by column chromatography (DCM/MeOH = 200/1 to 50/1), yield 67%. ^{1}H NMR (400 MHz, DMSO) δ 8.76 (dd, *J* = 4.1, 1.6 Hz, 1H), 8.47 (d, *J* = 8.6 Hz, 1H), 8.25 – 8.17 (m, 3H), 7.81 (dd, *J* = 8.3, 1.1 Hz, 1H), 7.68 (dd, *J* = 7.3, 1.2 Hz, 1H), 7.48 – 7.42 (m, 2H), 7.25 (s, 1H), 6.75 – 6.69 (m, 1H), 6.61 – 6.53 (m, 4H). ^{13}C NMR (151 MHz, DMSO) δ 165.89, 150.38, 143.50, 141.70, 136.01, 134.49, 133.31, 132.70, 130.42, 129.73 (q, 31.7 Hz), 129.41, 128.80, 128.33, 127.91, 126.35, 126.24, 125.67, 124.89, 123.08, 121.84, 121.64, 120.14 (q, 3.6 Hz), 110.87. LRMS (ESI) $[M+H]^+$ found m/z 417.0. HRMS (ESI) $[M+H]^+$ found m/z 417.1212 calcd for $C_{25}H_{16}F_3N_2O$ 417.1209.

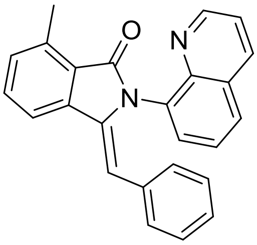

(*Z*)-3-Benzylidene-7-methyl-2-(quinolin-8-yl)isoindolin-1-one (3l).

Obtained as a white solid by column chromatography (DCM/MeOH = 200/1 to 50/1), yield 52%. ^{1}H NMR (400 MHz, DMSO) δ 8.77 (dd, *J* = 4.1, 1.6 Hz, 1H), 8.21 (dd, *J* = 8.3, 1.6 Hz, 1H), 7.99 (d, *J* = 7.7 Hz, 1H), 7.77 (dd, *J* = 8.2, 1.0 Hz, 1H), 7.66 (t, *J* = 7.6 Hz, 1H), 7.60 (dd, *J* = 7.3, 1.1 Hz, 1H), 7.47 – 7.38 (m, 3H), 6.96 (s, 1H), 6.72 – 6.64 (m, 1H), 6.58 – 6.49 (m, 4H), 2.67 (s, 3H). ^{13}C NMR (126 MHz, DMSO) δ 167.90, 150.19, 143.80, 138.99, 136.59, 135.93, 135.35, 133.90, 133.39, 132.15, 131.03, 130.48, 128.41, 128.30, 127.94, 126.12, 125.81, 125.59, 124.62, 121.47, 117.85, 106.86, 17.01. LRMS (ESI) $[M+H]^+$ found m/z 363.1. HRMS (ESI) $[M+H]^+$ found m/z 363.1494, calcd for $C_{25}H_{19}N_2O$ 363.1492.

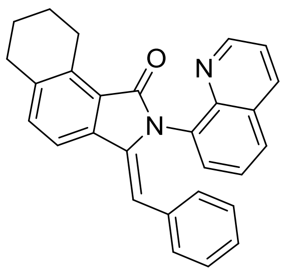

(*Z*)-3-Benzylidene-2-(quinolin-8-yl)-2,3,6,7,8,9-hexahydro-1*H*-benzo[*e*]isoindol-1-one (3m)

Obtained as a pale yellow solid by column chromatography (DCM/MeOH = 200/1 to 50/1), yield 49%. ^{1}H NMR (400 MHz, DMSO) δ 8.76 (dd, *J* = 4.0, 1.6 Hz, 1H), 8.20 (dd, *J* = 8.3, 1.3 Hz, 1H), 7.87 (d, *J* = 7.9 Hz, 1H), 7.76 (d, *J* = 7.4 Hz, 1H), 7.57 (d, *J* = 6.4 Hz, 1H), 7.42 (m, 3H), 6.87 (s, 1H), 6.68 (t, *J* = 6.6 Hz, 1H), 6.59 – 6.48 (m, 4H), 3.18 (s, 2H), 2.88 (s, 2H), 1.80 (s, 4H). ^{13}C NMR (126 MHz, DMSO) δ 168.09, 150.18, 143.87, 138.45, 136.92, 135.93, 135.62, 135.50, 134.07, 133.53, 133.33, 130.50, 128.35, 128.32, 127.99, 126.13, 125.74, 125.60, 124.18, 121.46, 117.31, 106.15, 29.11, 24.82, 22.27, 21.99. LRMS (ESI) $[M+H]^+$ found m/z 403.1. HRMS (ESI) $[M+H]^+$ found m/z 403.1801, calcd for $C_{28}H_{23}N_2O$ 403.1805.

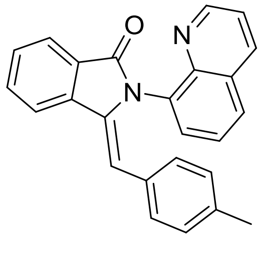

(*Z*)-3-(4-Methylbenzylidene)-2-(quinolin-8-yl)isoindolin-1-one (3n)

Obtained as a white solid by column chromatography (DCM/MeOH = 200/1 to 50/1), yield 81%. ^{1}H NMR (400 MHz, DMSO) δ 8.75 (dd, *J* = 4.1, 1.6 Hz, 1H), 8.24 (dd, *J* = 8.3, 1.6 Hz, 1H), 8.18 (d, *J* = 7.8 Hz, 1H), 7.87 (d, *J* = 7.5 Hz, 1H), 7.81 (t, *J* = 7.0 Hz, 2H), 7.65 (t, *J* = 7.4 Hz, 1H), 7.60 (dd, *J* = 7.3, 1.2 Hz, 1H), 7.47 – 7.39 (m, 2H), 6.99 (s, 1H), 6.37 (dd, *J* = 23.9, 8.0 Hz, 4H), 1.96 (s, 3H). ^{13}C NMR (126 MHz, DMSO) δ 167.18, 150.20, 143.80, 138.55, 135.89, 135.18, 135.09, 133.93, 132.64, 130.37, 130.19, 129.26, 128.37, 128.33, 127.83, 127.39, 126.73, 125.65, 122.99,

121.56, 120.35, 107.95, 20.52. LRMS (ESI) $[M+H]^+$ found m/z 363.1. HRMS (ESI) $[M+H]^+$ found m/z 363.1494, calcd for $C_{25}H_{19}N_2O$ 363.1492.

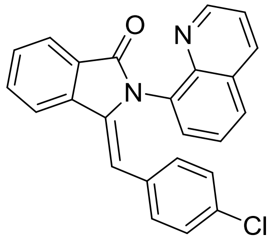

(*Z*)-3-(4-Chlorobenzylidene)-2-(quinolin-8-yl)isoindolin-1-one (3o)

Obtained as a pale yellow solid by column chromatography (DCM/MeOH = 200/1 to 50/1), yield 76%. ^{1}H NMR (400 MHz, DMSO) δ 8.75 (dd, *J* = 4.1, 1.6 Hz, 1H), 8.24 (dd, *J* = 8.3, 1.5 Hz, 1H), 8.18 (d, *J* = 7.8 Hz, 1H), 7.89 (d, *J* = 7.5 Hz, 1H), 7.83 (dd, *J* = 14.1, 7.3 Hz, 2H), 7.67 (m, 2H), 7.51 – 7.42 (m, 2H), 6.99 (s, 1H), 6.53 (dd, *J* = 19.5, 8.5 Hz, 4H).^{13}C NMR (126 MHz, DMSO) δ 167.15, 150.32, 143.54, 138.25, 136.15, 135.95, 133.63, 132.80, 132.10, 130.64, 130.55, 129.58, 129.47, 128.55, 128.32, 127.48, 125.94, 125.76, 123.09, 121.69, 120.51, 106.39. LRMS (ESI) $[M+H]^+$ found m/z 383.0. HRMS (ESI) $[M+H]^+$ found m/z 383.0952, calcd for $C_{24}H_{16}ClN_2O$ 383.0946.

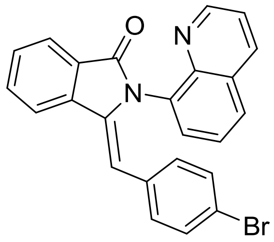

(*Z*)-3-(4-Bromobenzylidene)-2-(quinolin-8-yl)isoindolin-1-one (3p)

Obtained as a yellow solid by column chromatography (DCM/MeOH = 200/1 to 50/1), yield 79%.δ 8.74 (d, *J* = 2.7 Hz, 1H), 8.24 (d, *J* = 7.7 Hz, 1H), 8.19 (d, *J* = 7.6 Hz, 1H), 7.92 – 7.79 (m, 3H), 7.71 – 7.64 (m, 2H), 7.52 – 7.42 (m, 2H), 6.96 (s, 1H), 6.68 (d, *J* = 8.2 Hz, 2H), 6.44 (d, *J* = 8.0 Hz, 2H)^{13}C NMR (126 MHz, DMSO)δ 167.11, 150.31, 143.51, 138.24, 136.12, 135.93, 133.62, 132.80, 132.44, 130.64, 129.73, 129.58, 128.82, 128.52, 128.32, 127.47, 125.76, 123.08, 121.68, 120.51, 119.16, 106.41. LRMS (ESI) $[M+H]^+$ found m/z 427.0. HRMS (ESI) $[M+H]^+$ found m/z

427.0444, calcd for $C_{24}H_{16}BrN_2O$ 427.0441.

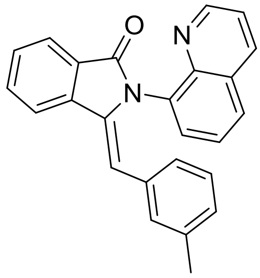

(*Z*)-3-(3-Methylbenzylidene)-2-(quinolin-8-yl)isoindolin-1-one (3q)

Obtained as a pale yellow solid by column chromatography (DCM/MeOH = 200/1 to 50/1), yield 58%. ^{1}H NMR (400 MHz, DMSO) δ 8.82 (dd, *J* = 4.1, 1.6 Hz, 1H), 8.28 (dd, *J* = 8.3, 1.5 Hz, 1H), 8.19 (d, *J* = 7.8 Hz, 1H), 7.88 (d, *J* = 7.5 Hz, 1H), 7.80 (dd, *J* = 13.2, 7.4 Hz, 2H), 7.66 (t, *J* = 7.4 Hz, 1H), 7.54 (dd, *J* = 7.3, 1.1 Hz, 1H), 7.49 (dd, *J* = 8.3, 4.1 Hz, 1H), 7.38 (t, *J* = 7.8 Hz, 1H), 7.01 (s, 1H), 6.60 (t, *J* = 7.5 Hz, 1H), 6.51 (t, *J* = 8.5 Hz, 2H), 6.18 (s, 1H), 1.59 (s, 3H). ^{13}C NMR (126 MHz, DMSO) δ 167.21, 150.36, 143.84, 138.53, 136.03, 135.36, 135.31, 134.00, 133.11, 132.68, 130.04, 129.38, 129.03, 128.45, 128.44, 127.43, 126.68, 126.38, 125.62, 125.18, 123.00, 121.57, 120.39, 107.87, 20.26. LRMS (ESI) $[M+H]^+$ found m/z 363.1. HRMS (ESI) $[M+H]^+$ found m/z 363.1498, calcd for $C_{25}H_{19}N_2O$ 363.1492.

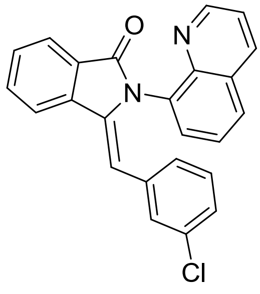

(*Z*)-3-(3-Chlorobenzylidene)-2-(quinolin-8-yl)isoindolin-1-one (3r)

Obtained as a white solid by column chromatography (DCM/MeOH = 200/1 to 50/1), yield 71%. ^{1}H NMR (400 MHz, DMSO) δ 8.81 (dd, *J* = 4.2, 1.7 Hz, 1H), 8.27 (dd, *J* = 8.3, 1.7 Hz, 1H), 8.18 (d, *J* = 7.8 Hz, 1H), 7.89 (d, *J* = 7.5 Hz, 1H), 7.82 (m, 2H), 7.68 (t, *J* = 7.5 Hz, 1H), 7.63 (dd, *J* = 7.3, 1.3 Hz, 1H), 7.50 – 7.42 (m, 2H), 6.99 (s, 1H), 6.76 (d, *J* = 8.0 Hz, 1H), 6.66 (t, *J* = 7.8 Hz, 1H), 6.59 (d, *J* = 7.6 Hz, 1H), 6.44

(s, 1H). ^{13}C NMR (126 MHz, DMSO) δ 167.17, 150.47, 143.56, 138.22, 136.36, 136.14, 135.41, 133.60, 132.82, 131.26, 130.26, 129.70, 128.75, 128.49, 127.98, 127.87, 127.50, 126.62, 125.80, 125.70, 123.10, 121.69, 120.55, 105.94. LRMS (ESI) $[M+H]^+$ found m/z 383.0. HRMS (ESI) $[M+H]^+$ found m/z 383.0956, calcd for $C_{24}H_{16}ClN_2O$ 383.0946.

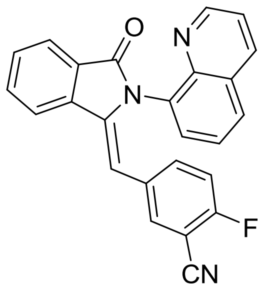

(*Z*)-2-Fluoro-5-((3-oxo-2-(quinolin-8-yl)isoindolin-1-ylidene)methyl)benzonitrile (3s)

Obtained as a pale yellow solid by column chromatography (DCM/MeOH = 200/1 to 50/1), yield 72%. ^{1}H NMR (400 MHz, DMSO) δ 8.78 (dd, *J* = 4.2, 1.6 Hz, 1H), 8.28 (dd, *J* = 8.3, 1.6 Hz, 1H), 8.18 (d, *J* = 7.7 Hz, 1H), 7.93 – 7.87 (m, 2H), 7.85 (t, *J* = 7.6 Hz, 1H), 7.77 (dd, *J* = 7.3, 1.1 Hz, 1H), 7.70 (t, *J* = 7.5 Hz, 1H), 7.55 (t, *J* = 7.8 Hz, 1H), 7.48 (dd, *J* = 8.3, 4.2 Hz, 1H), 7.01 – 6.94 (m, 2H), 6.87 (dd, *J* = 6.4, 2.0 Hz, 1H), 6.75 (t, *J* = 9.1 Hz, 1H).^{13}C NMR (126 MHz, DMSO) δ 167.00, 161.14, 159.10, 150.61, 143.13, 137.88, 137.10, 136.06, 135.03 (d, *J* = 8.6 Hz), 133.31, 132.96, 132.56, 130.85, 129.90, 128.83, 128.35, 127.55, 125.93, 123.18, 121.92, 120.61, 114.18 (d, *J* = 19.7 Hz), 113.17, 104.20, 97.75 (d, *J* = 15.6 Hz). LRMS (ESI) $[M+H]^+$ found m/z 392.0. HRMS (ESI) $[M+H]^+$ found m/z 392.1194, calcd for $C_{25}H_{15}FN_3O$ 392.1194.

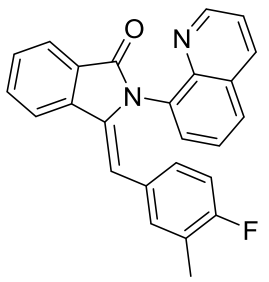

(*Z*)-3-(4-Fluoro-3-methylbenzylidene)-2-(quinolin-8-yl)isoindolin-1-one (3t)

Obtained as a pale yellow solid by column chromatography (DCM/MeOH = 200/1 to 50/1), yield 70%. ^{1}H NMR (400 MHz, DMSO)

δ 8.80 (dd, *J* = 4.1, 1.6 Hz, 1H), 8.30 (dd, *J* = 8.3, 1.6 Hz, 1H), 8.18 (d, *J* = 7.8 Hz, 1H), 7.87 (d, *J* = 7.6 Hz, 1H), 7.85 – 7.79 (m, 2H), 7.66 (t, *J* = 7.4 Hz, 1H), 7.59 (dd, *J* = 7.3, 1.2 Hz, 1H), 7.50 (dd, *J* = 8.3, 4.2 Hz, 1H), 7.43 (t, *J* = 7.8 Hz, 1H), 6.97 (s, 1H), 6.56 – 6.49 (m, 1H), 6.43 (t, *J* = 9.1 Hz, 1H), 6.23 (d, *J* = 7.4 Hz, 1H), 1.53 (s, 3H).^{13}C NMR (126 MHz, DMSO) δ 167.13, 159.74, 157.81, 150.42, 143.70, 138.35, 136.02, 135.64, 133.88, 132.71, 131.54 (d, *J* = 5.1 Hz), 130.23, 129.44, 129.24 (d, *J* = 3.5 Hz), 128.47, 128.42, 127.44, 127.28 (d, *J* = 8.1 Hz), 125.67, 123.02, 121.83 (d, *J* = 17.5 Hz), 112.92 (d, *J* = 22.4 Hz), 121.65, 120.39, 106.76, 13.44 (d, *J* = 3.0 Hz). LRMS (ESI) $[M+H]^+$ found m/z 381.1. HRMS (ESI) $[M+H]^+$ found m/z 381.1396, calcd for $C_{25}H_{18}FN_2O$ 381.1398.

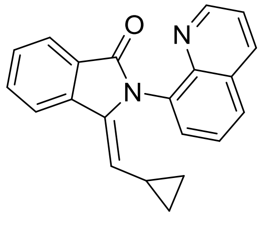

(*Z*)-3-(Cyclopropylmethylene)-2-(quinolin-8-yl)isoindolin-1-one (3u)

Obtained as a yellow solid by column chromatography (DCM/MeOH = 200/1 to 50/1), yield 61%. ^{1}H NMR (400 MHz, DMSO) δ 8.85 (dd, *J* = 4.2, 1.7 Hz, 1H), 8.49 (dd, *J* = 8.3, 1.6 Hz, 1H), 8.14 (dd, *J* = 8.3, 1.2 Hz, 1H), 7.94 (dd, *J* = 7.3, 1.3 Hz, 1H), 7.90 (d, *J* = 7.8 Hz, 1H), 7.80 (d, *J* = 7.5 Hz, 1H), 7.76 (d, *J* = 7.5 Hz, 1H), 7.70 (td, *J* = 7.8, 1.0 Hz, 1H), 7.61 (dd, *J* = 8.3, 4.2 Hz, 1H), 7.55 (t, *J* = 7.5 Hz, 1H), 5.35 (d, *J* = 9.7 Hz, 1H), 0.39 – 0.21 (m, 3H), 0.11 – 0.03 (m, 1H), -0.08 – -0.16 (m, 1H).^{13}C NMR

(126 MHz, DMSO) δ 166.89, 151.03, 144.68, 137.86, 136.40, 134.81, 134.56, 132.20, 130.71, 129.33, 128.65, 128.30, 126.97, 126.30, 122.74, 122.12, 119.50, 114.19, 8.38, 8.12, 8.00.LRMS (ESI) $[M+H]^+$ found m/z 313.1. HRMS (ESI) $[M+H]^+$ found m/z 313.1342, calcd for $C_{21}H_{17}N_2O$ 313.1335.

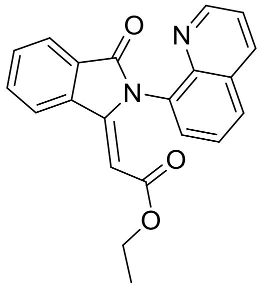

(*Z*)-ethyl 2-(3-oxo-2-(quinolin-8-yl)isoindolin-1-ylidene)acetate (3v)

Obtained as a yellow solid by column chromatography (DCM/MeOH = 200/1 to 50/1), yield 60%. ^{1}H NMR (400 MHz, DMSO) δ 8.80 (dd, *J* = 4.1, 1.6 Hz, 1H), 8.46 (dd, *J* = 8.3, 1.6 Hz, 1H), 8.23 (d, *J* = 7.7 Hz, 1H), 8.08 (dd, *J* = 8.2, 1.2 Hz, 1H), 7.91 (d, *J* = 7.4 Hz, 1H), 7.86 – 7.78 (m, 2H), 7.72 (m, 2H), 7.56 (dd, *J* = 8.3, 4.2 Hz, 1H), 6.30 (s, 1H), 3.31 (dq, *J* = 10.9, 7.1 Hz, 1H), 3.16 (dq, *J* = 10.9, 7.1 Hz, 1H), 0.59 (t, *J* = 7.1 Hz, 3H).^{13}C NMR (126 MHz, DMSO) δ 167.64, 163.74, 150.52, 144.18, 142.89, 137.59, 136.35, 133.77, 133.45, 131.21, 129.94, 128.83, 128.49, 127.40, 126.04, 123.42, 121.77, 121.37, 96.18, 59.51, 13.36. LRMS (ESI) $[M+H]^+$ found m/z 345.1. HRMS (ESI) $[M+H]^+$ found m/z 345.1237, calcd for $C_{21}H_{17}N_2O_3$ 345.1234.

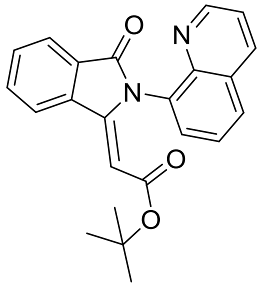

(*Z*)-*tert*-Butyl 2-(3-oxo-2-(quinolin-8-yl)isoindolin-1-ylidene)acetate (3w)

Obtained as a yellow solid by column chromatography (DCM/MeOH = 200/1 to 50/1), yield 67%. ^{1}H NMR (400 MHz, DMSO) ^{1}H NMR (400 MHz, D_2O) δ 8.79 (dd, *J* = 4.1, 1.5 Hz, 1H), 8.45 (d, *J* = 8.2 Hz, 1H), 8.21 (d, *J* = 7.7 Hz, 1H), 8.07 (d, *J* = 8.2 Hz,

1H), 7.89 (d, *J* = 7.5 Hz, 1H), 7.86 – 7.77 (m, 2H), 7.75 – 7.68 (m, 2H), 7.56 (dd, *J* = 8.3, 4.1 Hz, 1H), 6.23 (s, 1H), 0.81 (s, 9H).^{13}C NMR (126 MHz, DMSO)δ 167.63, 162.92, 150.39, 144.04, 142.11, 137.70, 136.35, 133.78, 133.39, 131.01, 130.10, 128.59, 127.40, 126.20, 123.34, 121.71, 121.26, 98.00, 79.59, 27.07.. LRMS (ESI) $[M+H]^+$ found m/z 373.1. HRMS (ESI) $[M+H]^+$ found m/z 373.1557, calcd for $C_{23}H_{21}N_2O_3$ 373.1547.

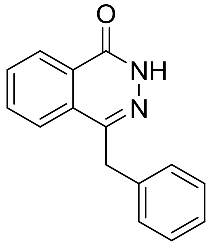

4-Benzylphthalazin-1(2*H*)-one (4a)

Obtained as a pale white solid, yield 95%. ^{1}H NMR (400 MHz, DMSO) δ 12.60 (s, 1H), 8.25 (dd, *J* = 7.8, 1.0 Hz, 1H), 7.93 (d, *J* = 7.6 Hz, 1H), 7.88 – 7.83 (m, 1H), 7.80 (t, *J* = 7.5 Hz, 1H), 7.33 – 7.25 (m, 4H), 7.18 (t, *J* = 6.9 Hz, 1H), 4.29 (s, 2H).^{13}C NMR (126 MHz, DMSO) δ 159.44, 145.26, 138.26, 133.45, 131.48, 129.19, 128.56, 127.93, 126.44, 126.04, 125.70, 37.65. LRMS (ESI) $[M+H]^+$ found m/z 237.1. HRMS (ESI) $[M+H]^+$ found m/z 237.1023, calcd for $C_{15}H_{14}N_2O$ 237.1022.

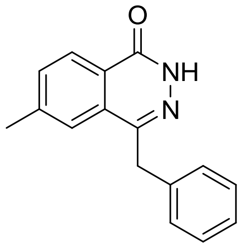

4-Benzyl-6-methylphthalazin-1(2*H*)-one (4b)

Obtained as a pale yellow solid, yield 77%. ^{1}H NMR (400 MHz, DMSO) δ 12.50 (s, 1H), 8.14 (d, *J* = 8.1 Hz, 1H), 7.76 (s, 1H), 7.62 (d, *J* = 8.1 Hz, 1H), 7.33 – 7.26 (m, 4H), 7.19 (t, *J* = 6.9 Hz, 1H), 4.27 (s, 2H), 2.45 (s, 3H).^{13}C NMR (101 MHz, DMSO) δ 159.39, 145.06, 143.78, 138.28, 132.68, 129.35, 128.60, 128.50, 126.38, 126.03, 125.66, 125.19, 37.45, 21.55. LRMS (ESI) $[M+H]^+$ found m/z 251.1. HRMS (ESI) $[M+H]^+$ found m/z 251.1180 calcd for $C_{16}H_{15}N_2O$ 251.1179.

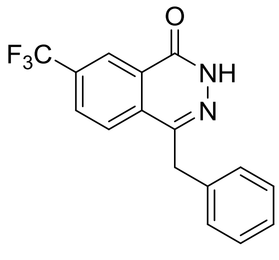

4-Benzyl-7-(trifluoromethyl)phthalazin-1(2*H*)-one (4k)

Obtained as a pale yellow solid, yield 91%. ^{1}H NMR (400 MHz, DMSO) δ 12.92 (s, 1H), 8.47 (s, 1H), 8.20 (d, *J* = 8.6 Hz, 1H), 8.14 (d, *J* = 8.5 Hz, 1H), 7.34 – 7.24 (m, 4H), 7.19 (t, *J* = 6.6 Hz, 1H), 4.35 (s, 2H). ^{13}C NMR (126 MHz, DMSO) δ 158.51, 144.59, 137.71, 131.69, 131.12, 130.86, 129.40 (d, *J* = 3.1 Hz), 128.49, 128.21, 127.35, 126.43, 124.39, 123.00 (d, *J* = 3.8 Hz), 122.22, 37.50. LRMS (ESI) $[M+H]^+$ found m/z 305.1. HRMS (ESI) $[M+H]^+$ found m/z 305.0900, calcd for $C_{16}H_{12}F_3N_2O$ 305.0896.

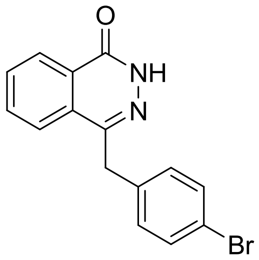

4-(4-Bromobenzyl)phthalazin-1(2*H*)-one (4p)

Obtained as a pale yellow solid, yield 97%. ^{1}H NMR (500 MHz, DMSO) δ 12.55 (s, 1H), 8.26 (d, *J* = 7.3 Hz, 1H), 7.92 (d, *J* = 7.3 Hz, 1H), 7.89 – 7.84 (m, 1H), 7.84 – 7.77 (m, 1H), 7.47 (d, *J* = 7.7 Hz, 2H), 7.28 (d, *J* = 7.5 Hz, 2H), 4.28 (s, 2H). ^{13}C NMR (126 MHz, DMSO) δ 159.28, 144.72, 137.56, 133.37, 131.40, 131.25, 130.81, 129.02, 127.84, 125.95, 125.40, 119.46, 36.82. LRMS (ESI) $[M+H]^+$ found m/z 315.0. HRMS (ESI) $[M+H]^+$ found m/z 315.0130, calcd for $C_{15}H_{12}BrN_2O$ 315.0128.

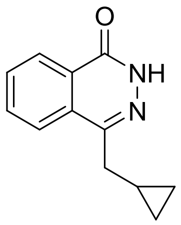

4-(Cyclopropylmethyl)phthalazin-1(2*H*)-one (4u)

Obtained as a pale yellow solid, yield 93%. ^{1}H NMR (400 MHz, DMSO) δ 12.48 (s, 1H), 8.26 (d, *J* = 7.7 Hz, 1H), 8.02 (d, *J* = 8.0 Hz, 1H), 7.93 (t, *J* = 7.4 Hz, 1H), 7.84 (t, *J* = 7.4 Hz, 1H), 2.84 (d, *J* = 6.8 Hz, 2H), 1.18 – 1.05 (m, 1H), 0.48 (d, *J* = 7.8 Hz, 2H), 0.24 (d, *J* = 4.6 Hz, 2H).^{13}C NMR (126 MHz, DMSO) δ 159.30, 146.05, 133.35, 131.24, 129.31, 127.59, 125.84, 125.28, 35.84, 9.54, 4.72. LRMS (ESI) $[M+H]^+$ found m/z 201.1. HRMS (ESI) $[M+H]^+$ found m/z 201.1022, calcd for $C_{12}H_{13}N_2O$ 201.1022.

(D) Copies of ^{1}H NMR and ^{13}C NMR spectra for the products

N-(Quinolin-8-yl)benzamide (1a)

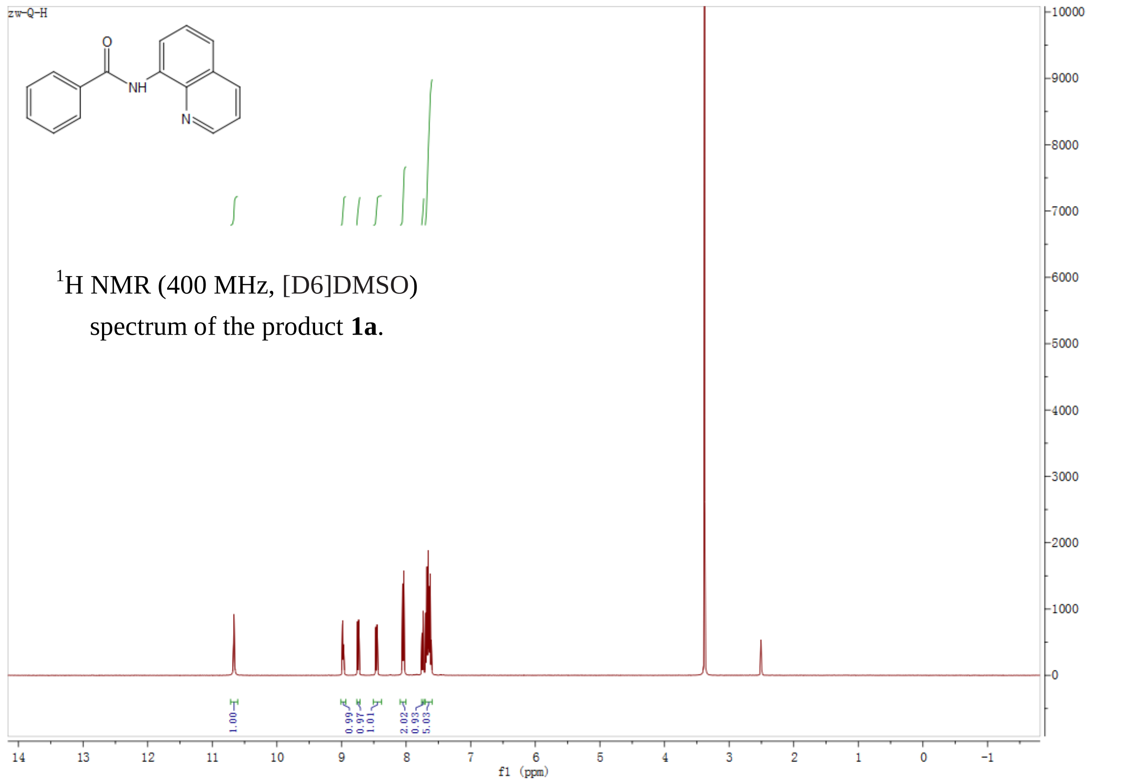

4-Methyl-*N*-(quinolin-8-yl)benzamide (1b)

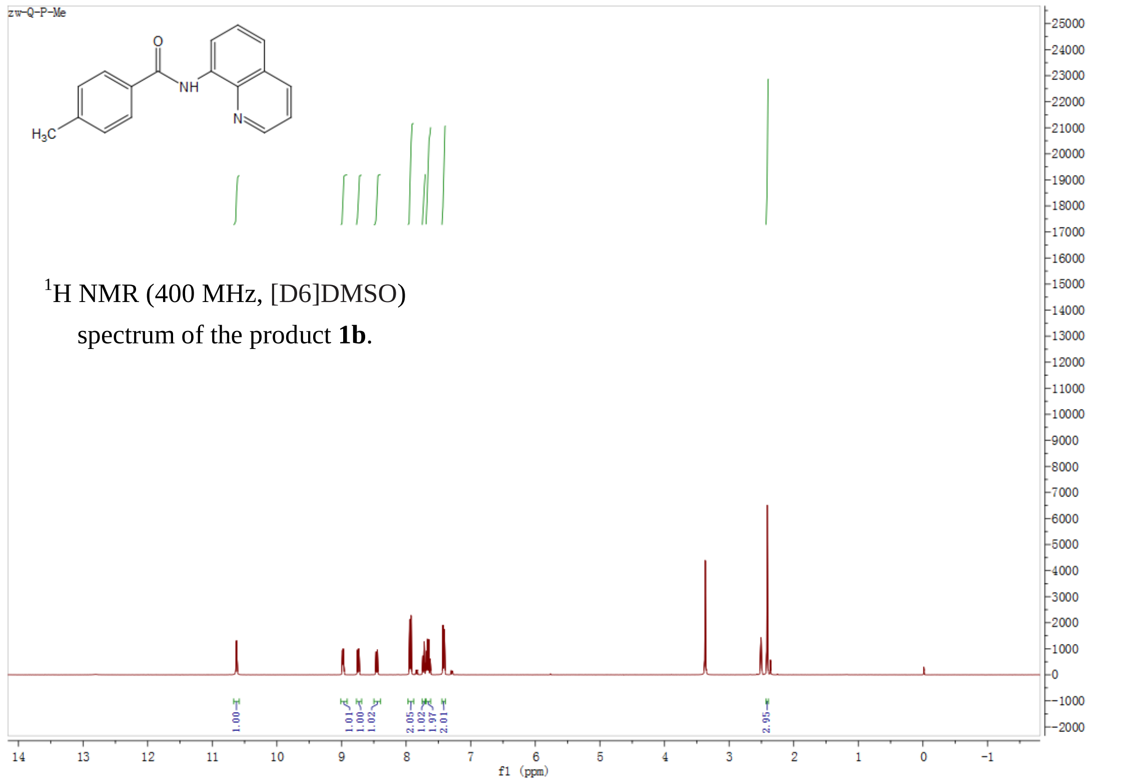

N-(quinolin-8-yl)-4-(trifluoromethoxy)benzamide (1c)

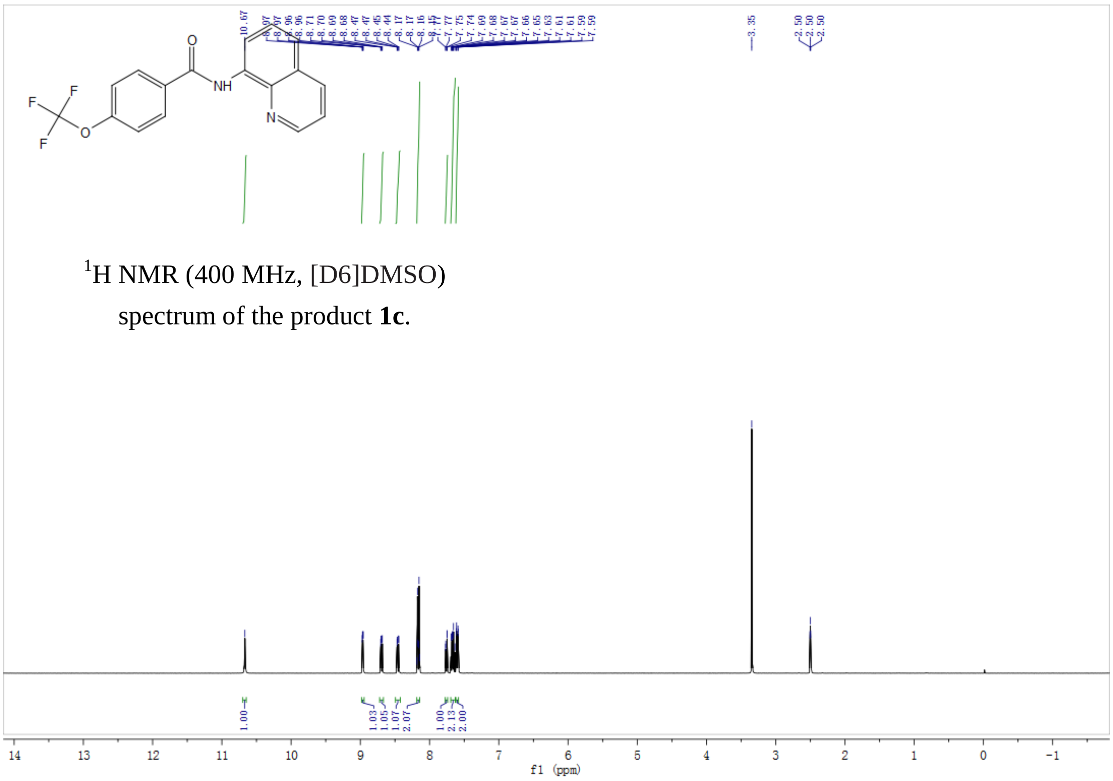

^{1}H NMR (400 MHz, [D6]DMSO) spectrum of the product **1c**.

4-Methoxy-*N*-(quinolin-8-yl)benzamide (1d)

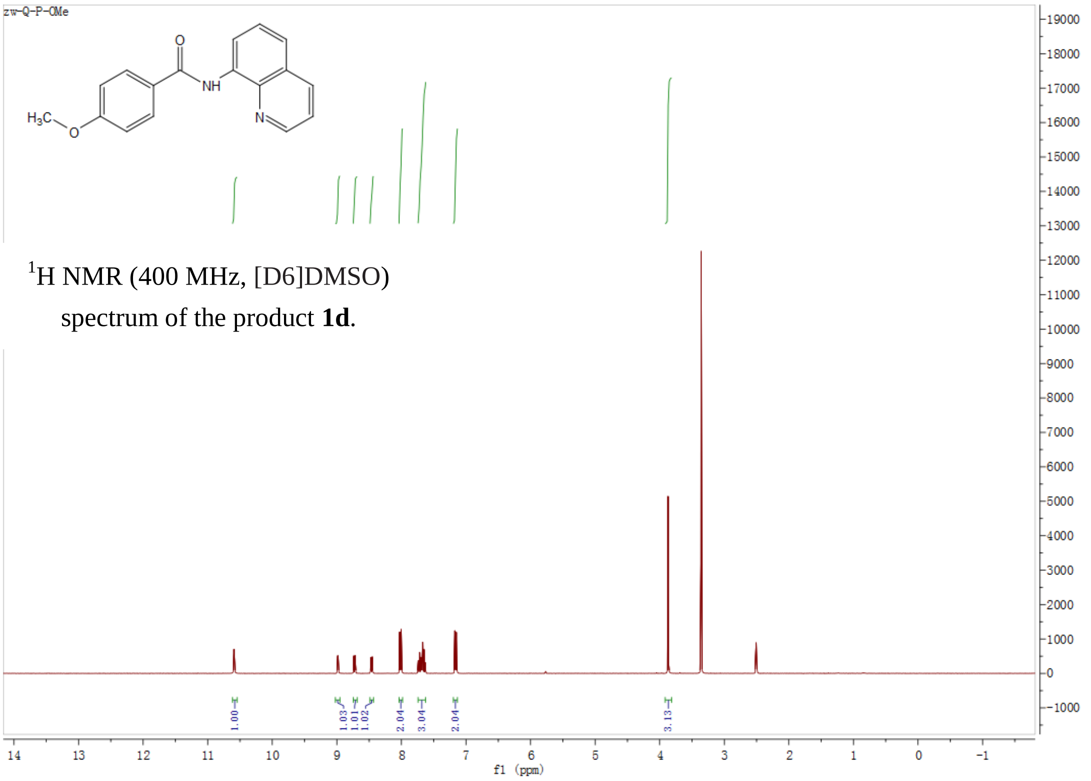

^{1}H NMR (400 MHz, [D6]DMSO) spectrum of the product **1d**.

N-(Quinolin-8-yl)-4-(trifluoromethyl)benzamide (1e)

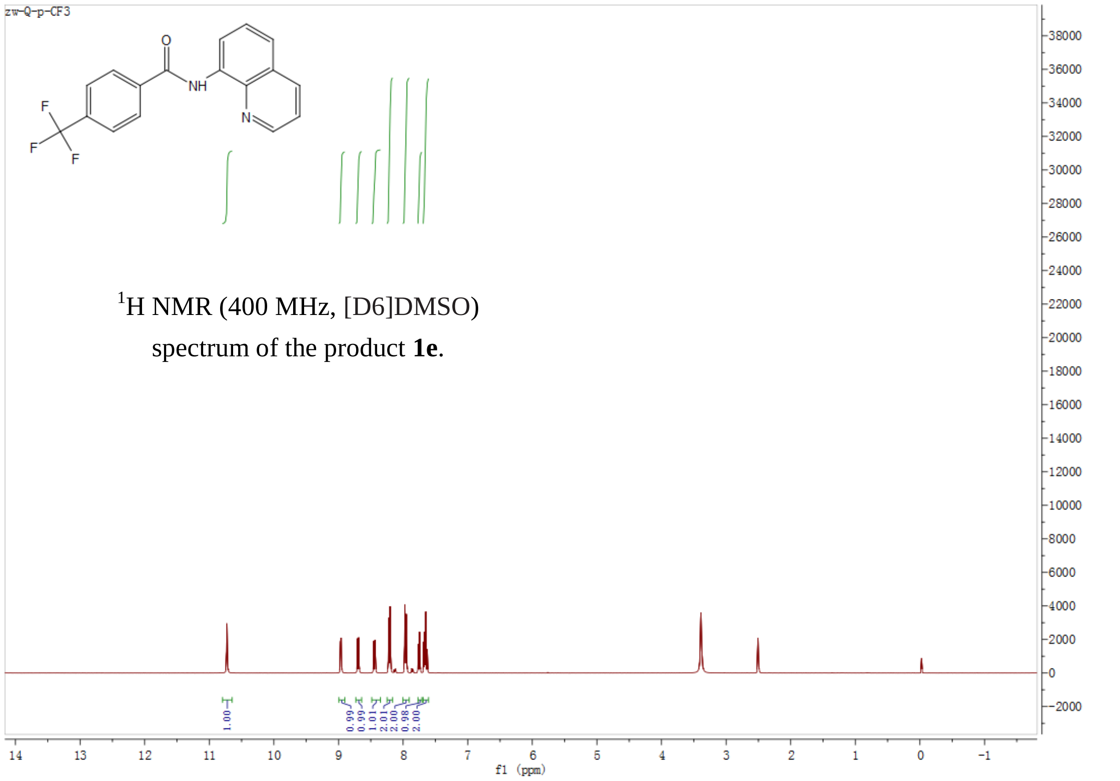

Methyl 4-(quinolin-8-ylcarbamoyl)benzoate (1f)

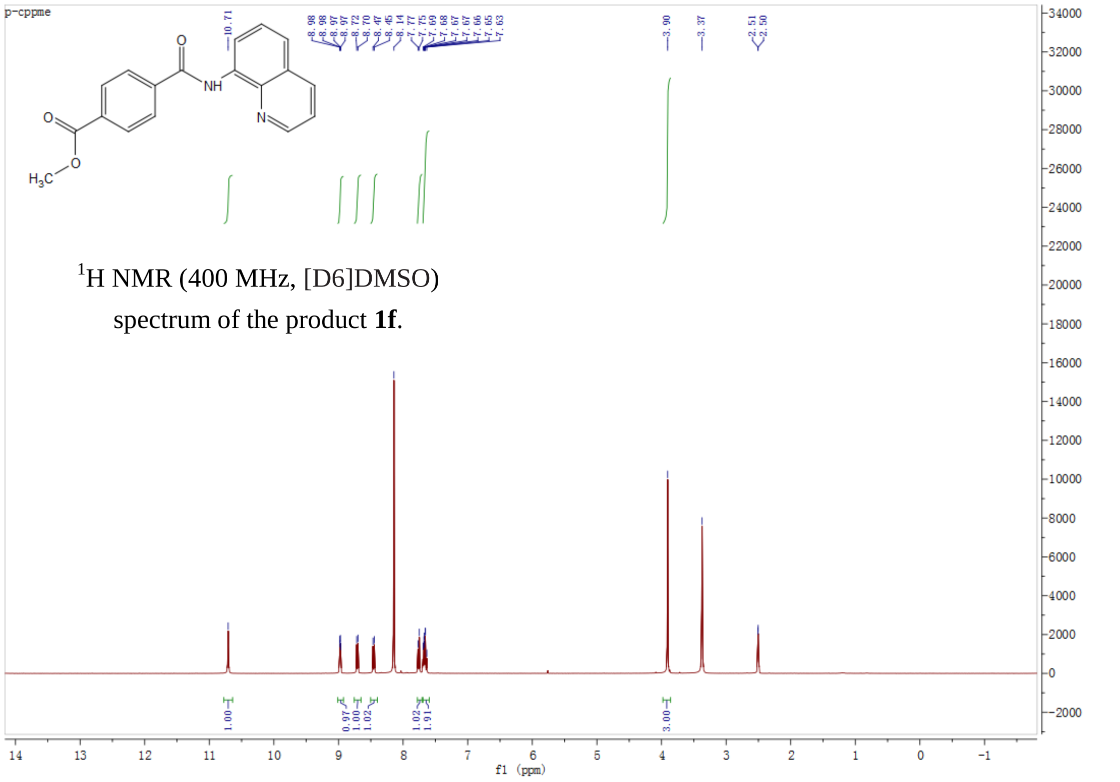

4-Fluoro-*N*-(quinolin-8-yl)benzamide (1g)

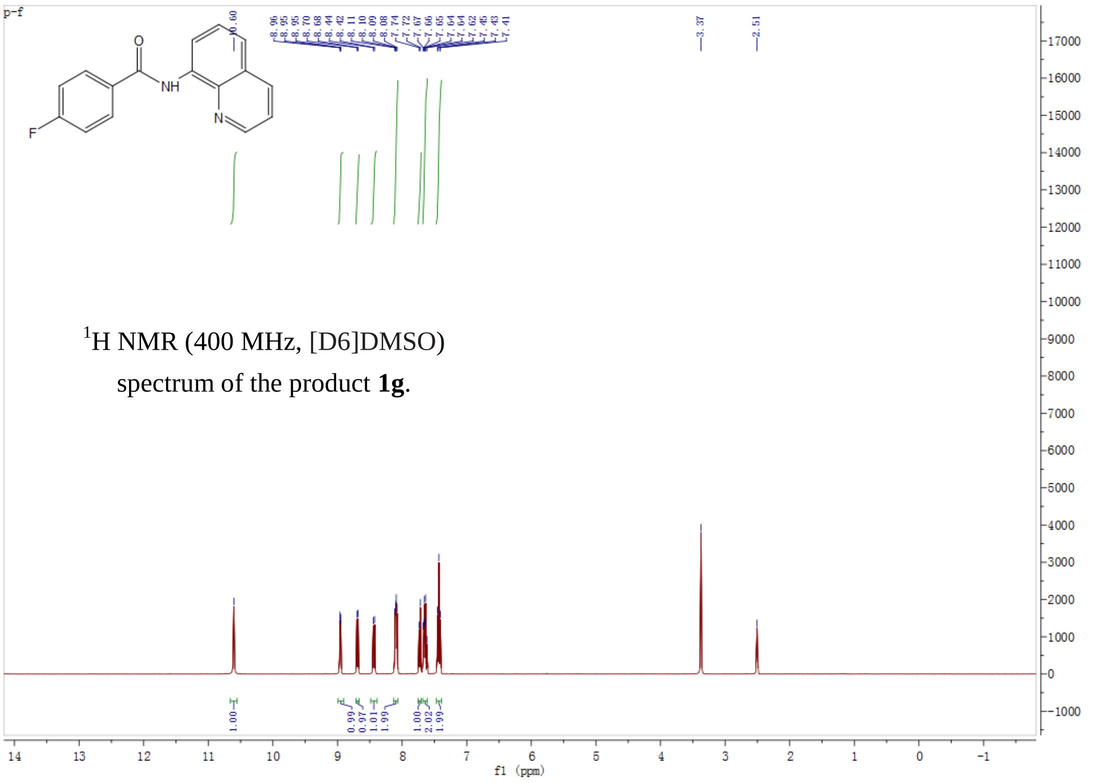

4-Chloro-*N*-(quinolin-8-yl)benzamide (1h)

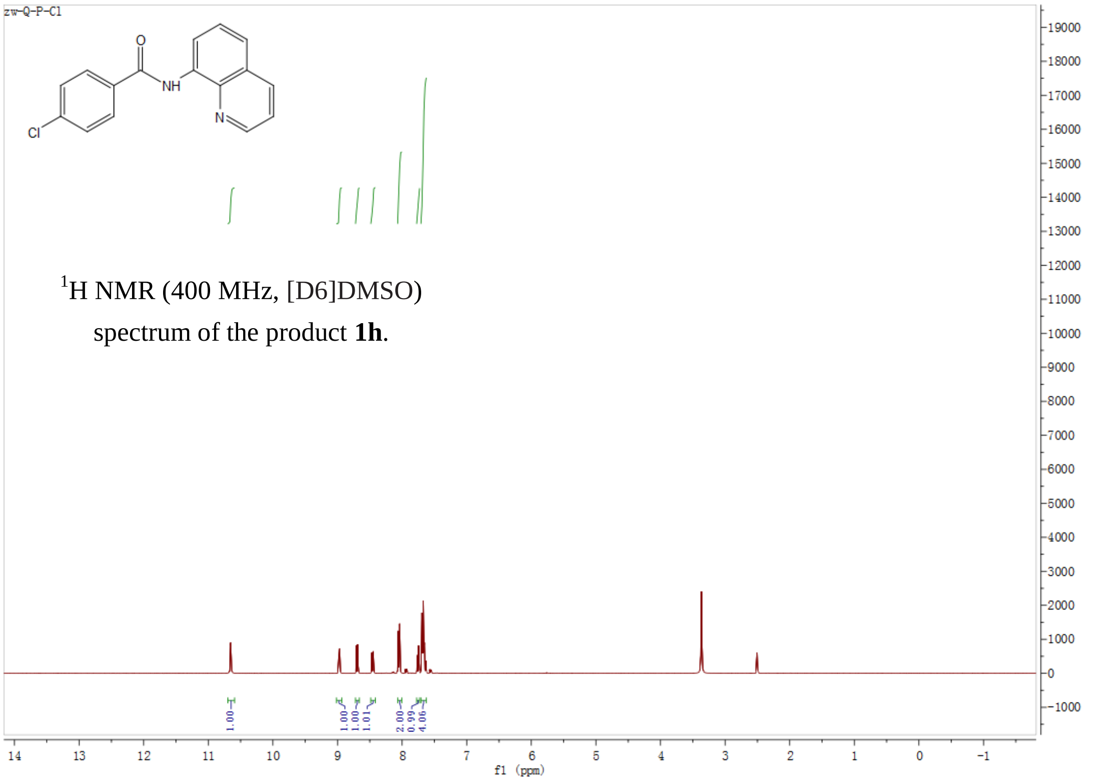

N-(quinolin-8-yl)-4-vinylbenzamide (1i)

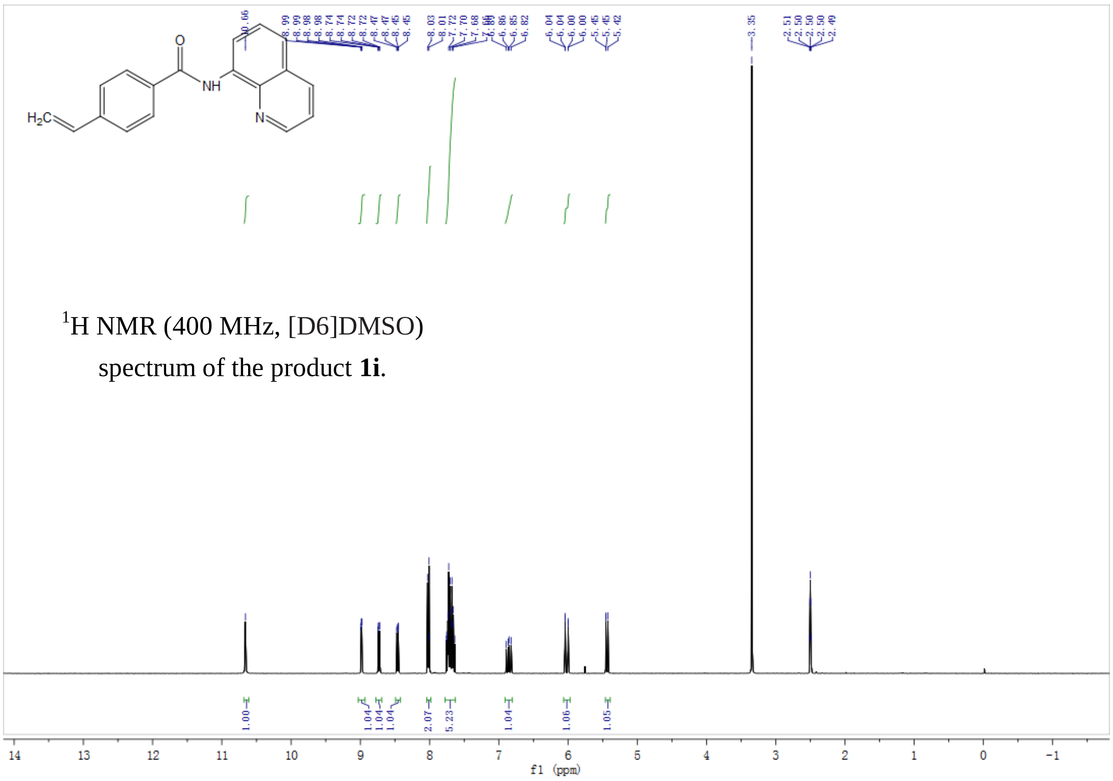

^{1}H NMR (400 MHz, [D6]DMSO) spectrum of the product **1i**.

3-Methyl-*N*-(quinolin-8-yl)benzamide (1j)

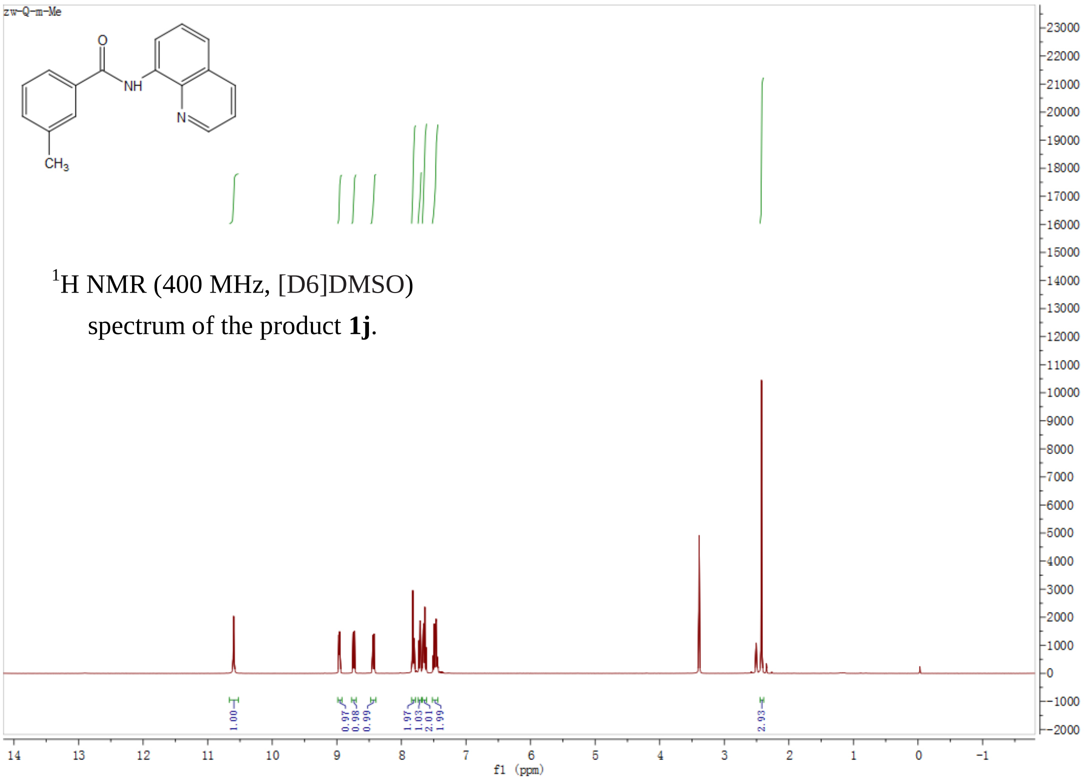

^{1}H NMR (400 MHz, [D6]DMSO) spectrum of the product **1j**.

N-(Quinolin-8-yl)-3-(trifluoromethyl)benzamide (1k)

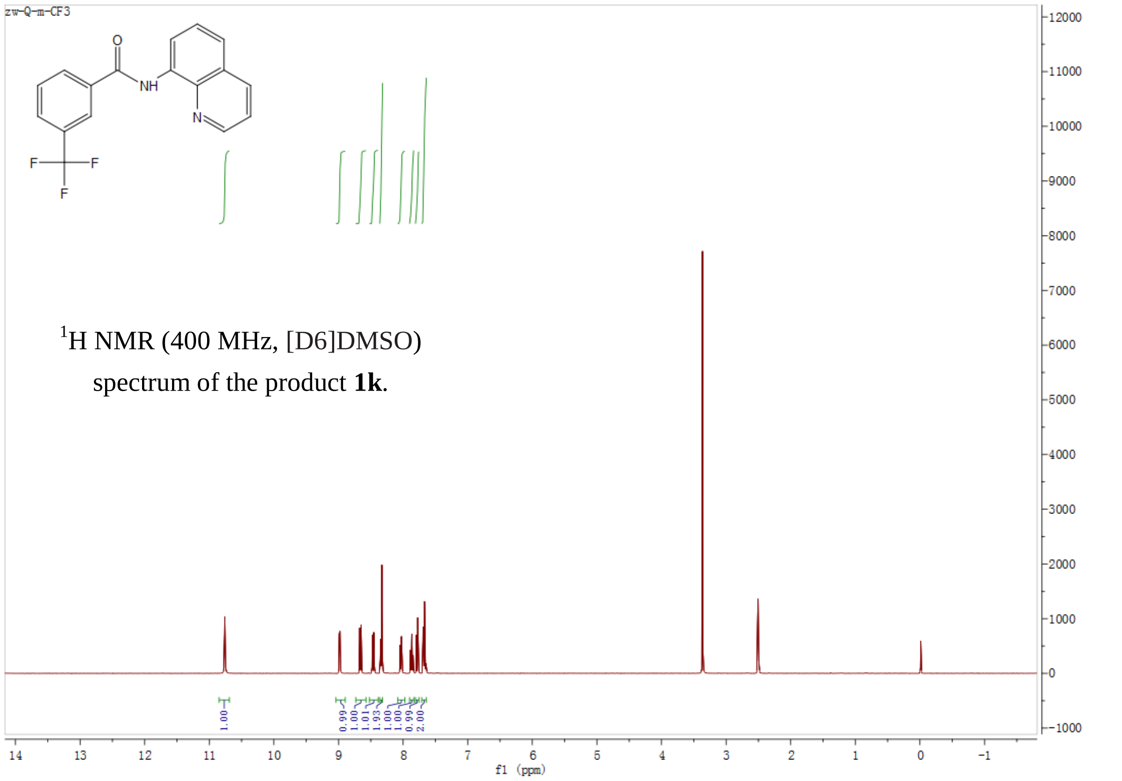

2-Methyl-*N*-(quinolin-8-yl)benzamide (1l)

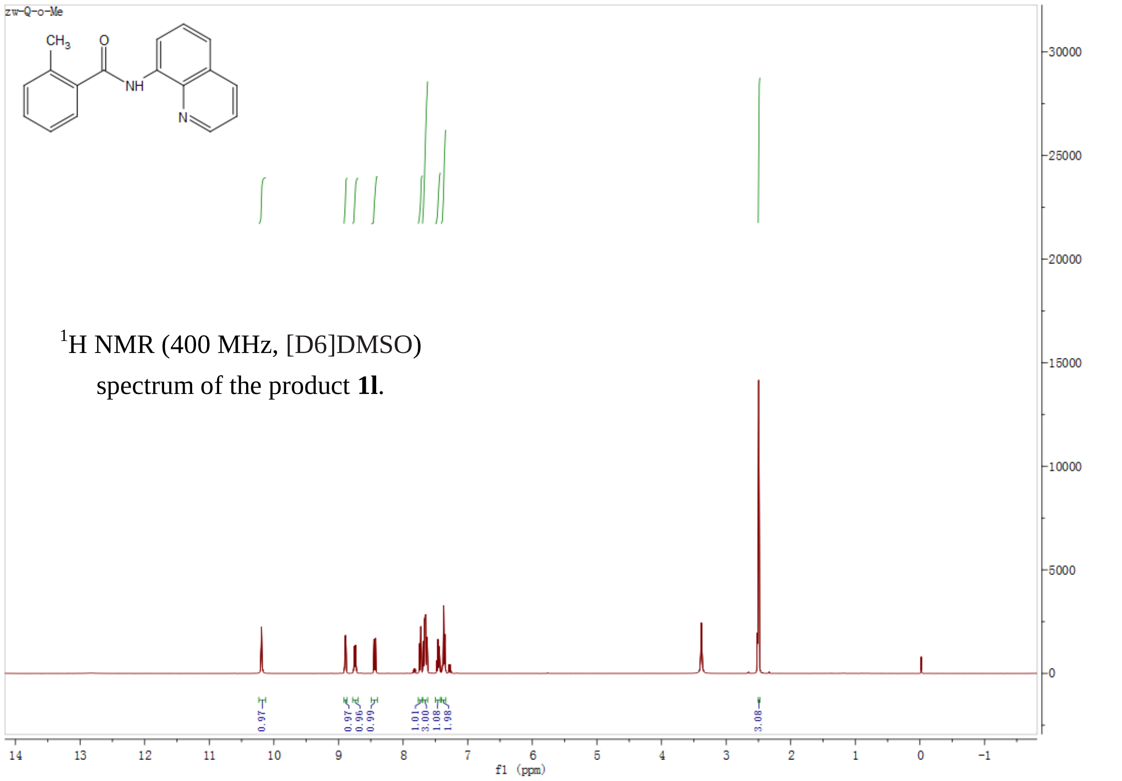

***N*-(Quinolin-8-yl)-5,6,7,8-tetrahydronaphthalene-1-carboxamide (1m)**

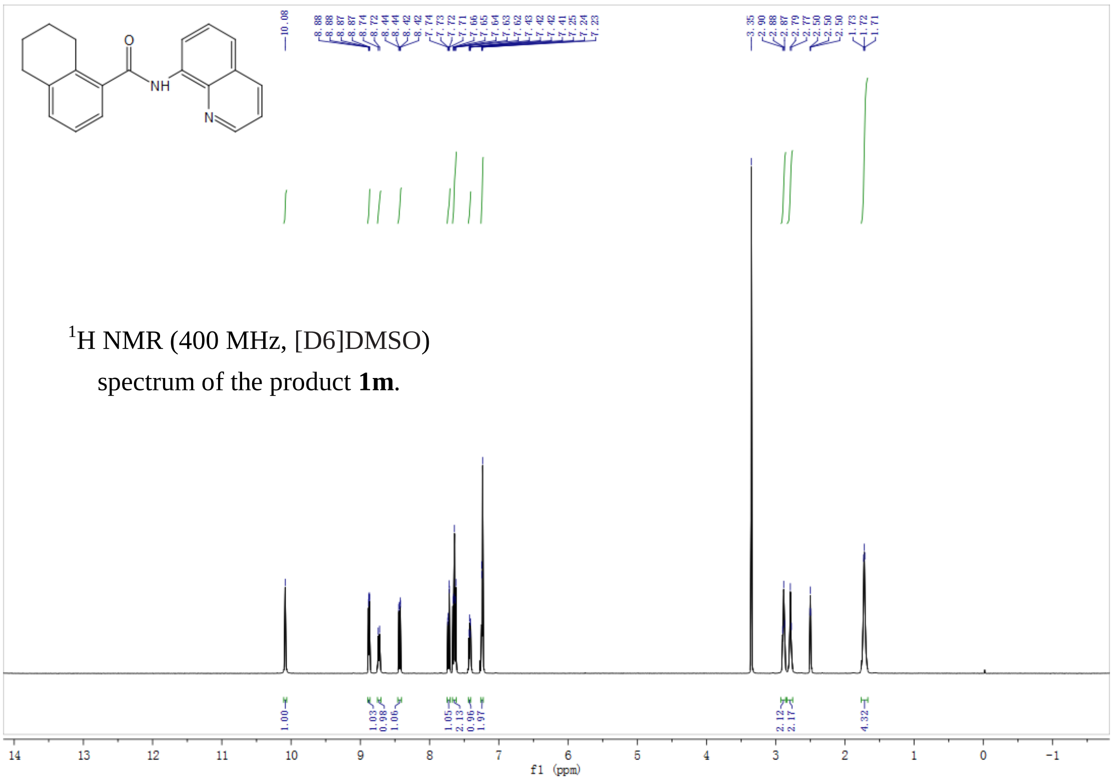

^{1}H NMR (400 MHz, [D6]DMSO) spectrum of the product **1m**.

(*Z*)-3-Benzylidene-2-(quinolin-8-yl)isoindolin-1-one (3a)

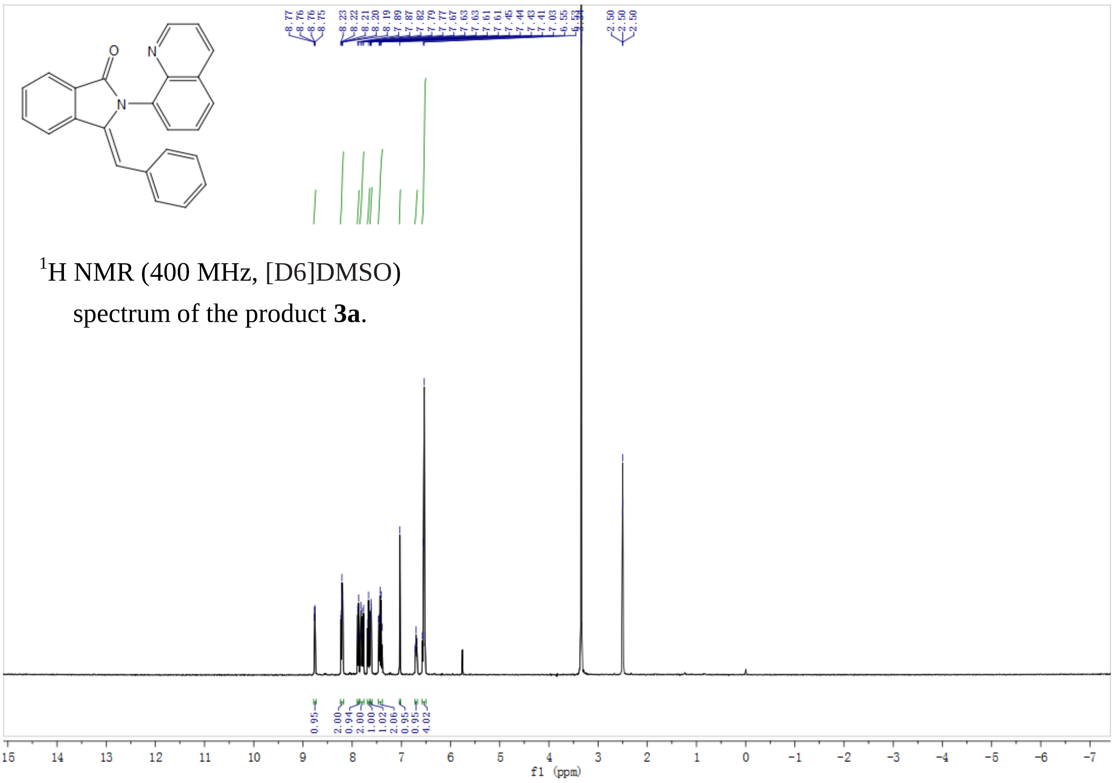

^{1}H NMR (400 MHz, [D6]DMSO) spectrum of the product **3a**.

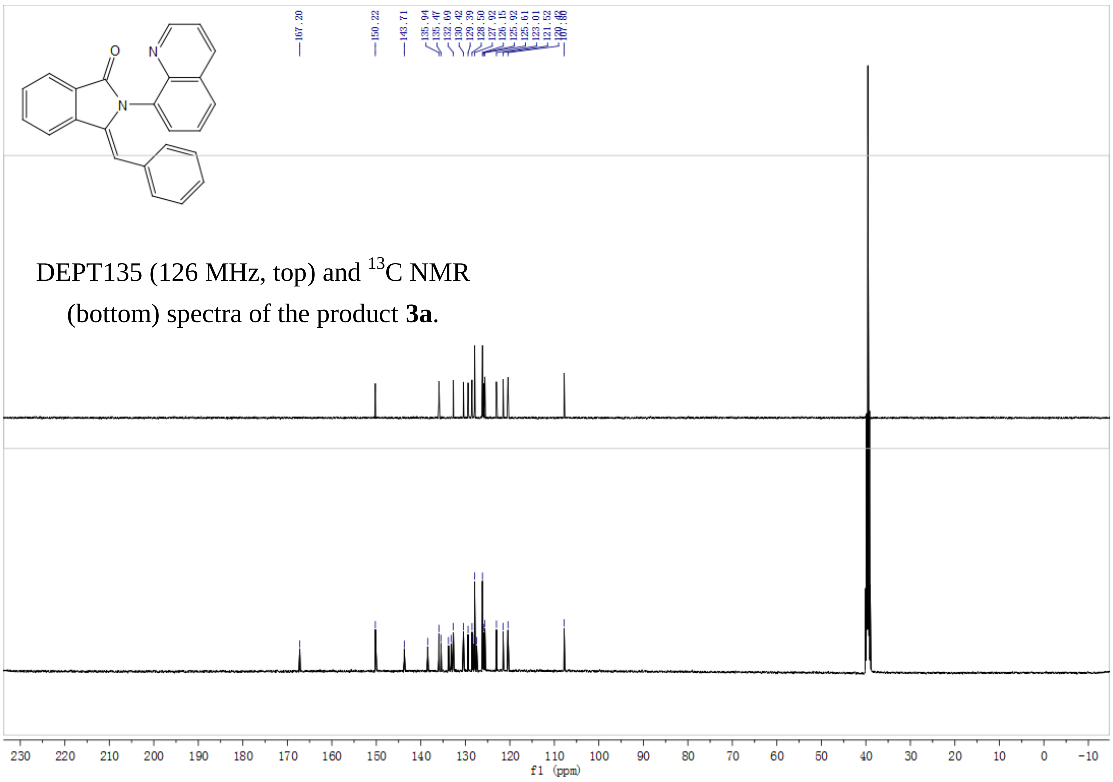

DEPT135 (126 MHz, top) and ^{13}C NMR (bottom) spectra of the product **3a**.

(*Z*)-3-Benzylidene-5-methyl-2-(quinolin-8-yl)isoindolin-1-one (3b)

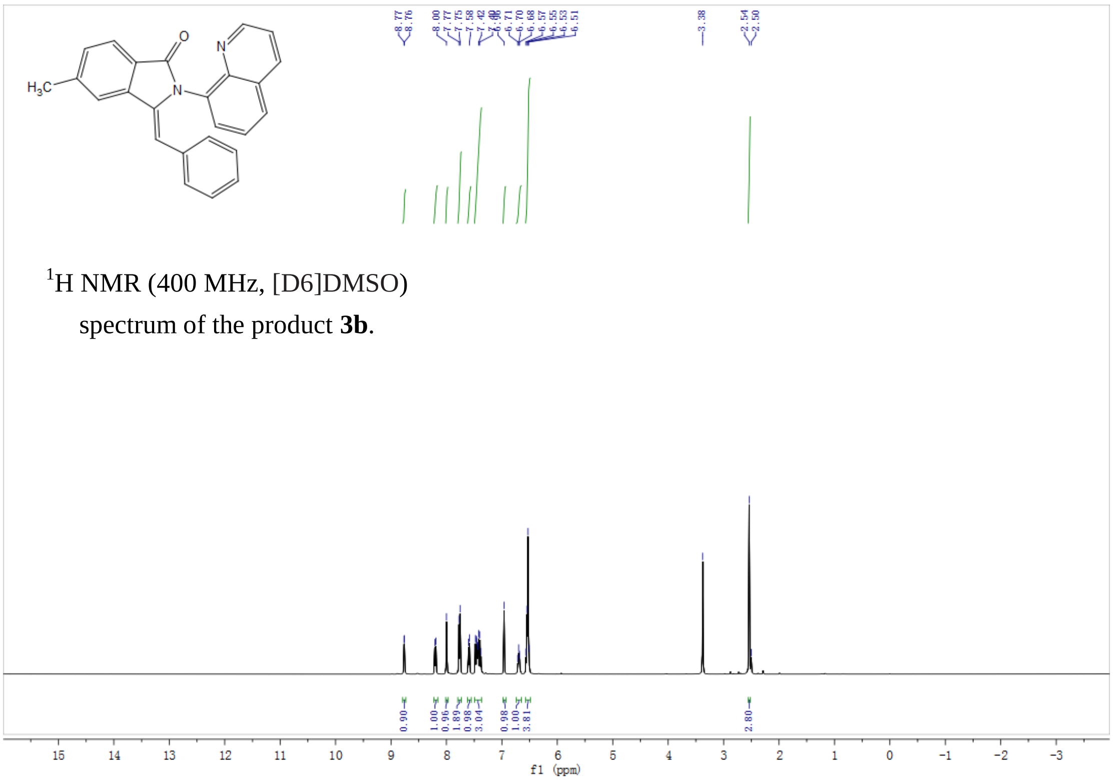

^{1}H NMR (400 MHz, [D6]DMSO) spectrum of the product **3b**.

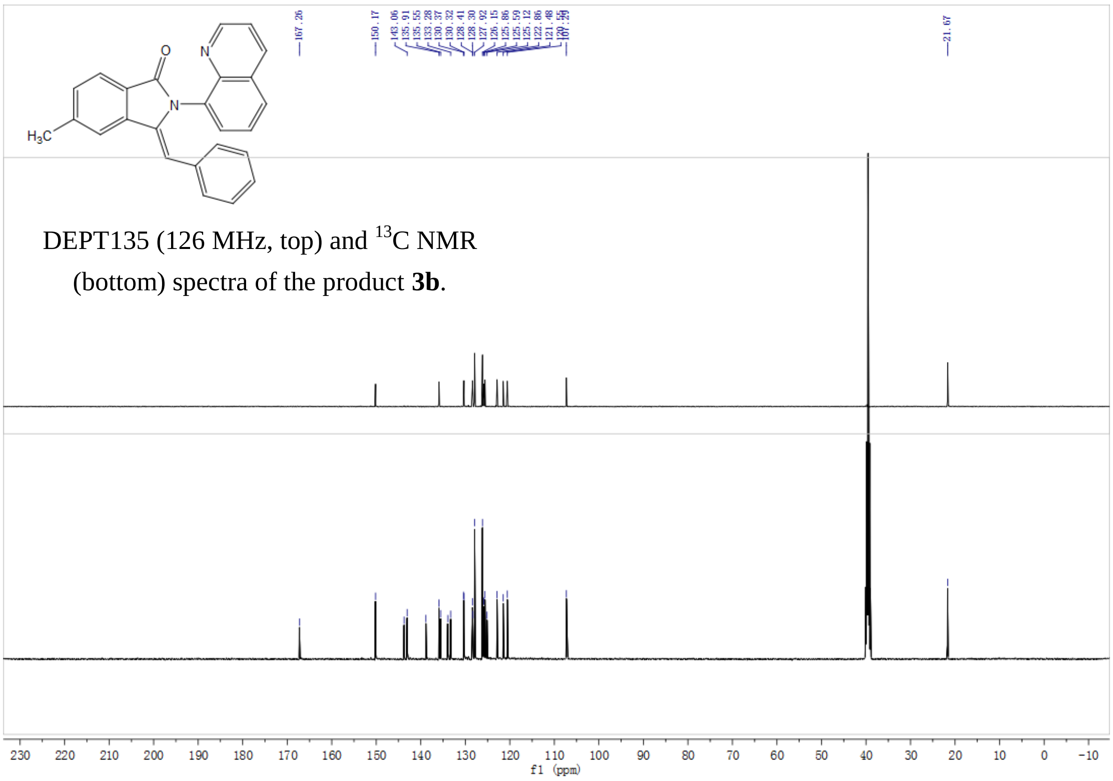

DEPT135 (126 MHz, top) and ^{13}C NMR (bottom) spectra of the product **3b**.

(*Z*)-3-Benzylidene-2-(quinolin-8-yl)-5-(trifluoromethoxy)isoindolin-1-one (3c)

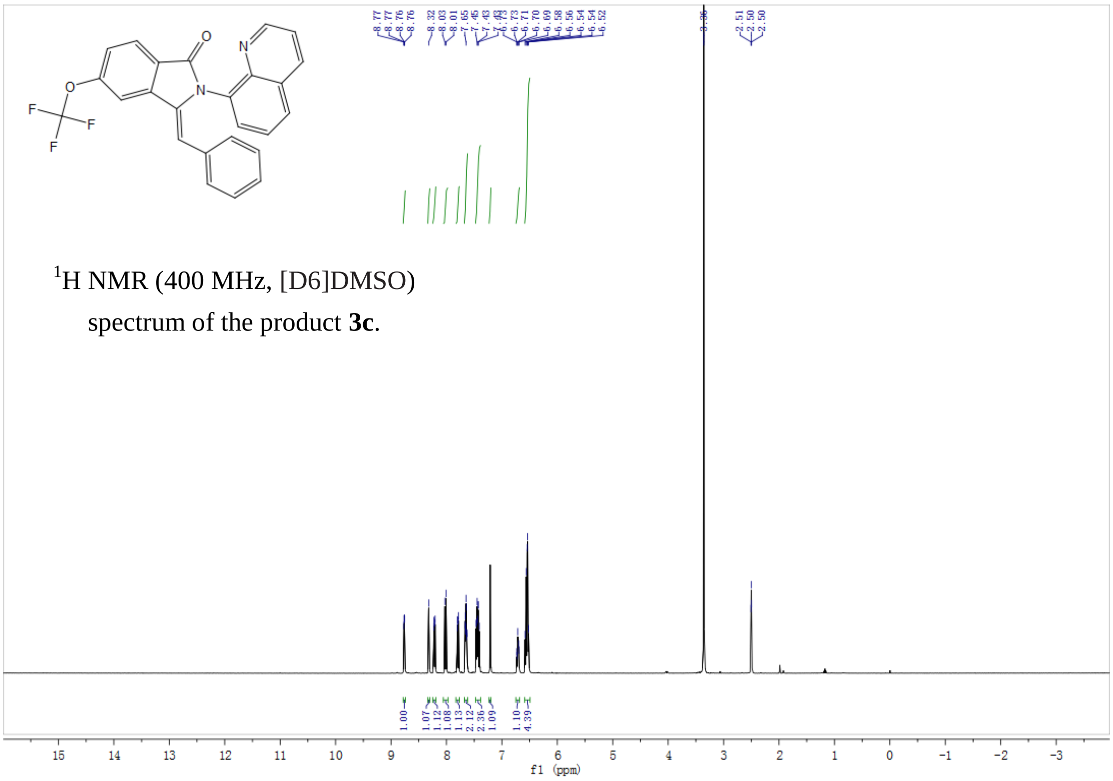

^{1}H NMR (400 MHz, [D6]DMSO) spectrum of the product **3c**.

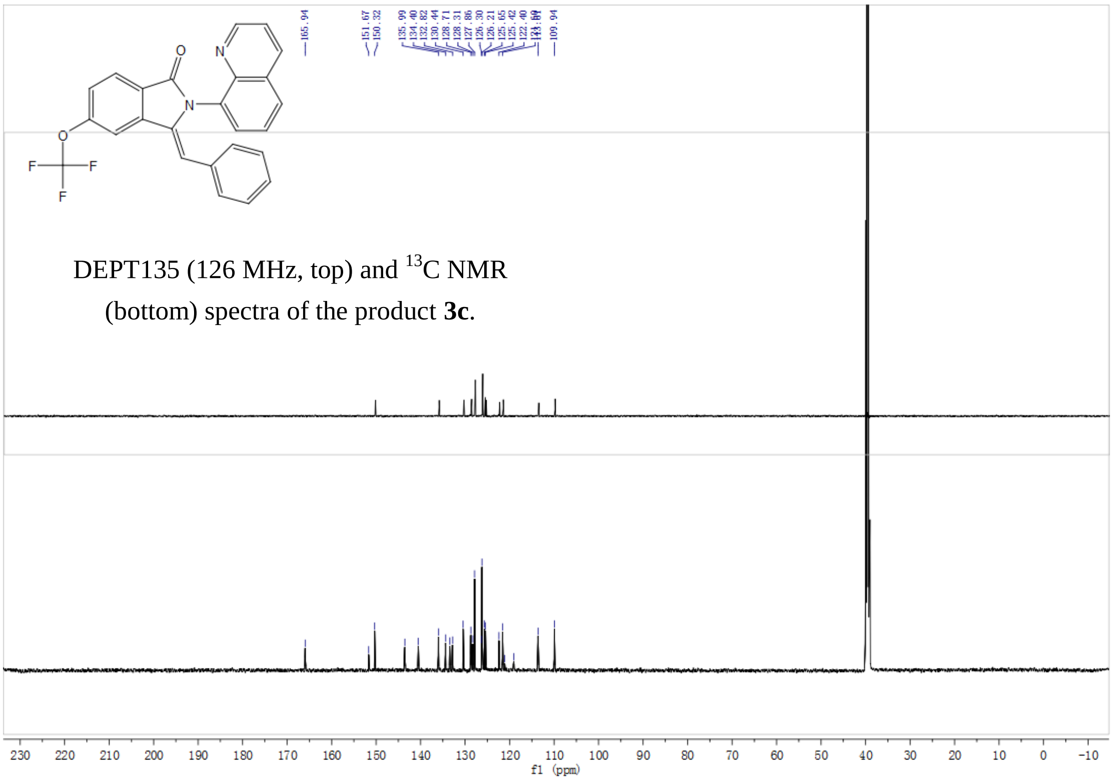

DEPT135 (126 MHz, top) and ^{13}C NMR (bottom) spectra of the product **3c**.

(*Z*)-3-Benzylidene-5-methoxy-2-(quinolin-8-yl)isoindolin-1-one (3d)

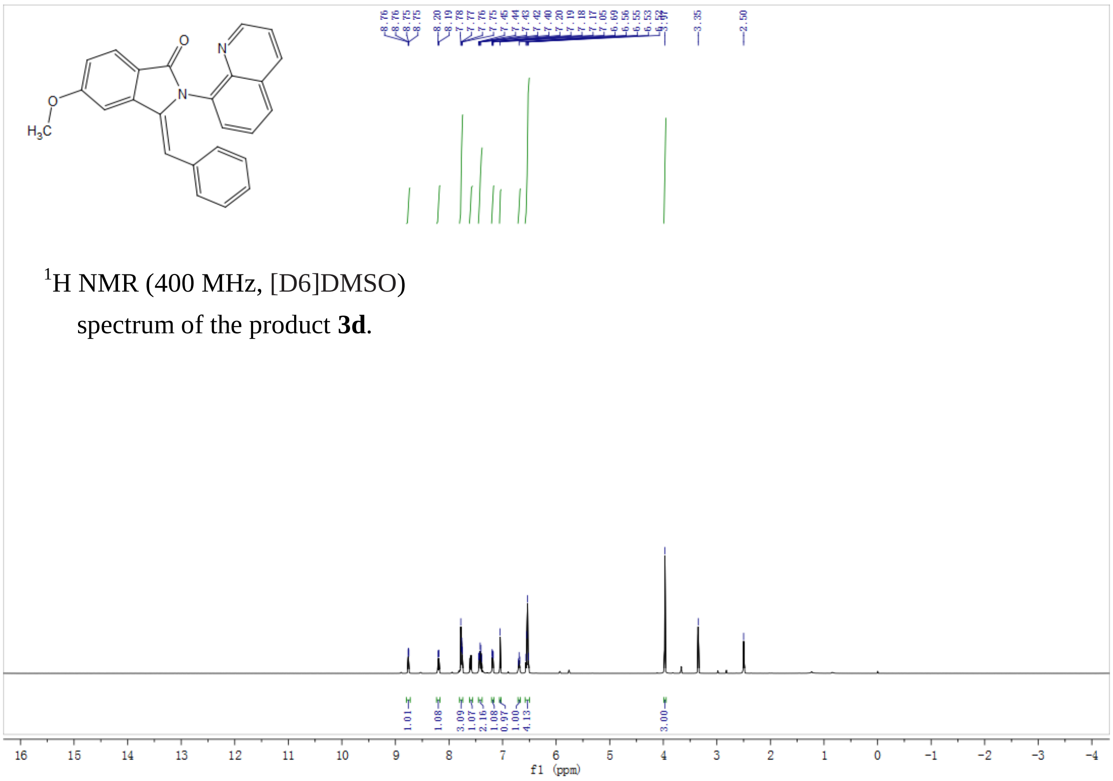

^{1}H NMR (400 MHz, [D6]DMSO) spectrum of the product **3d**.

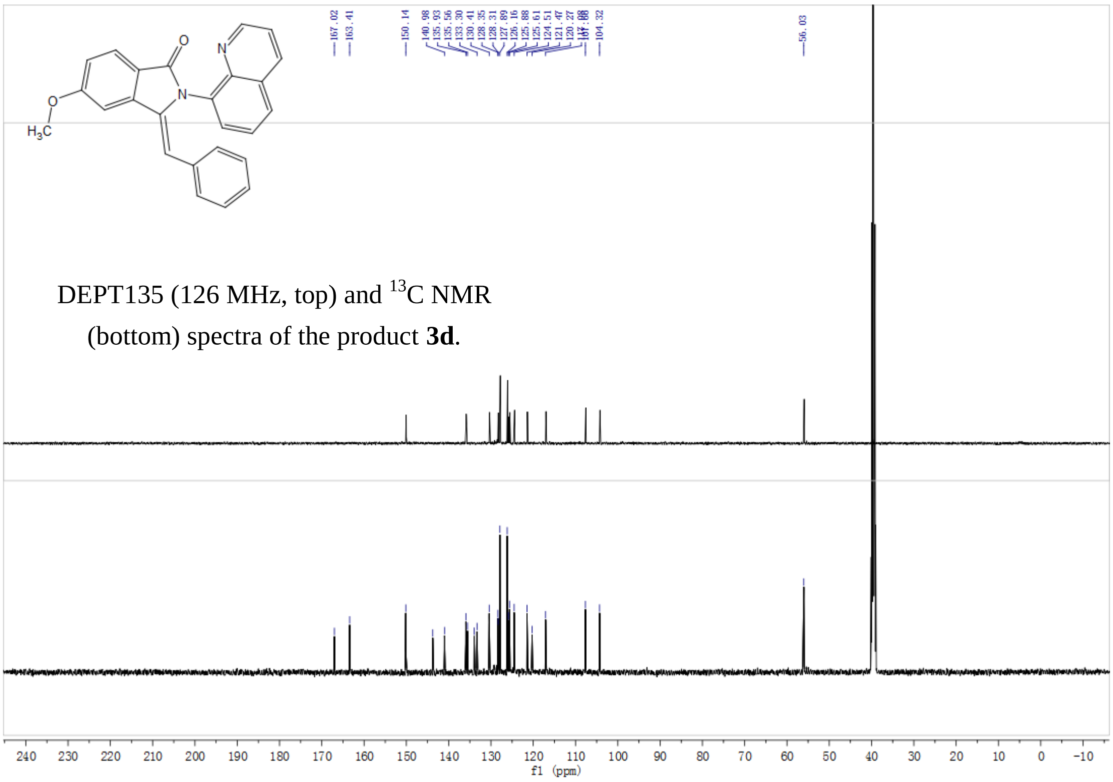

DEPT135 (126 MHz, top) and ^{13}C NMR (bottom) spectra of the product **3d**.

(*Z*)-3-Benzylidene-2-(quinolin-8-yl)-5-(trifluoromethyl)isoindolin-1-one (3e)

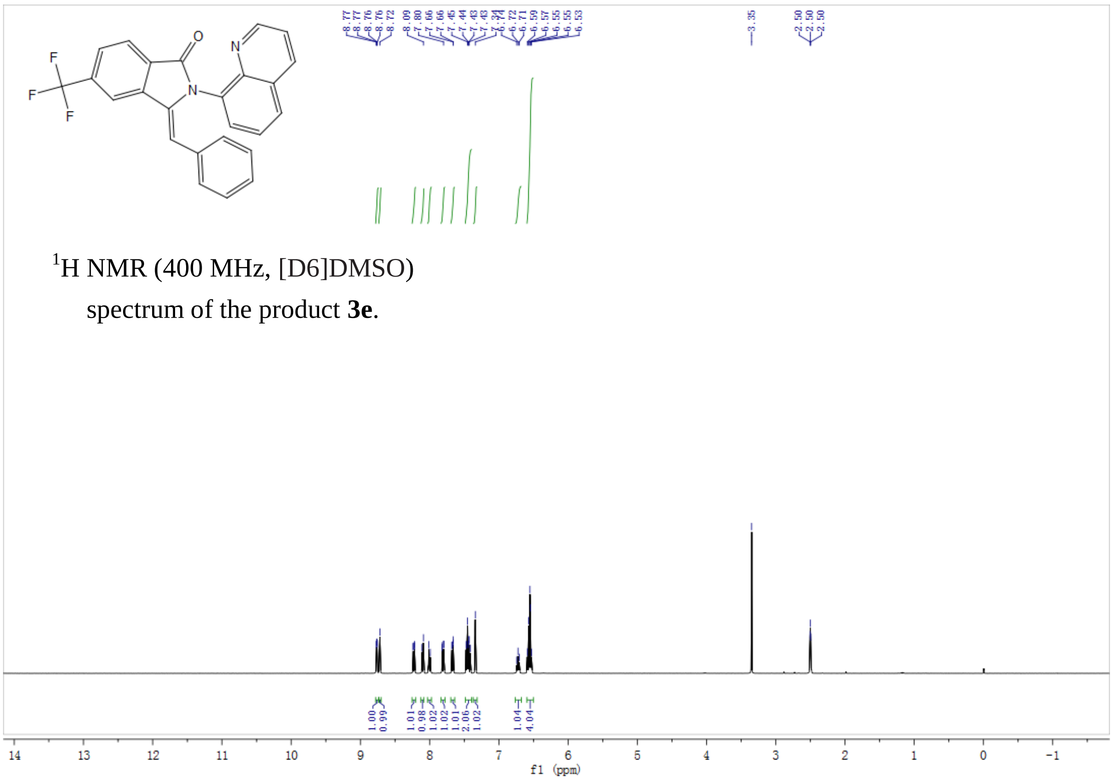

^{1}H NMR (400 MHz, [D6]DMSO) spectrum of the product **3e**.

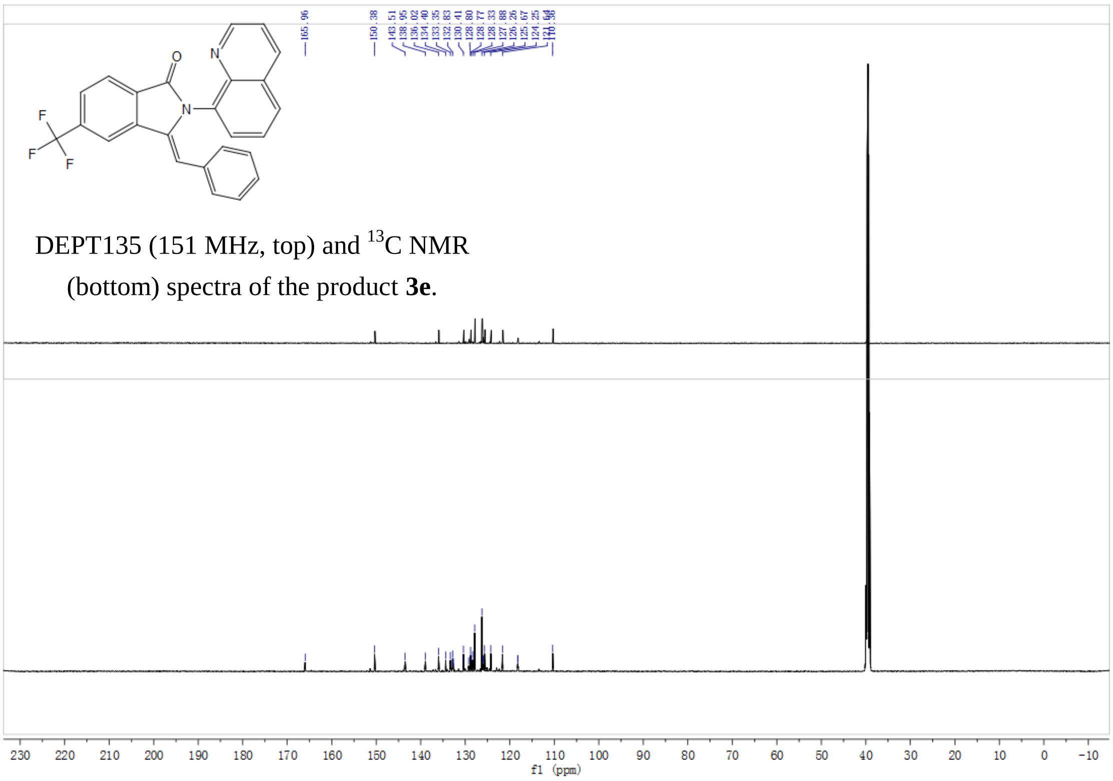

DEPT135 (151 MHz, top) and ^{13}C NMR (bottom) spectra of the product **3e**.

(*Z*)-Methyl 3-benzylidene-1-oxo-2-(quinolin-8-yl)isoindoline-5-carboxylate (3f)

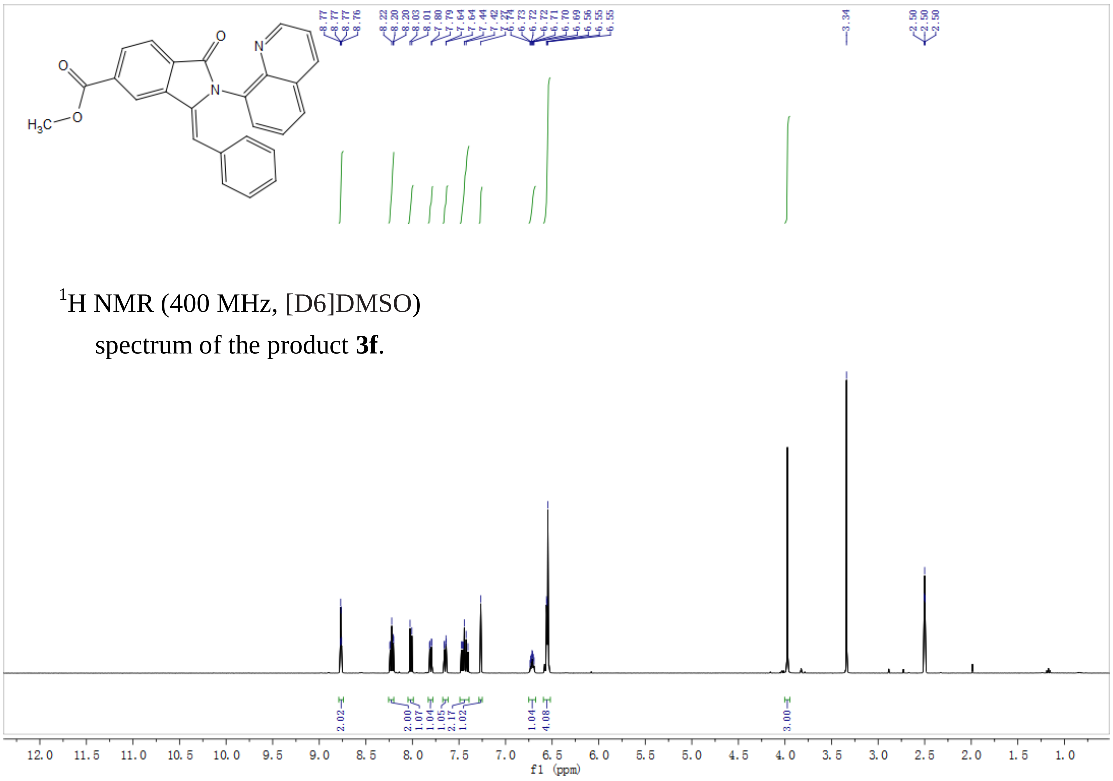

^{1}H NMR (400 MHz, [D6]DMSO) spectrum of the product **3f**.

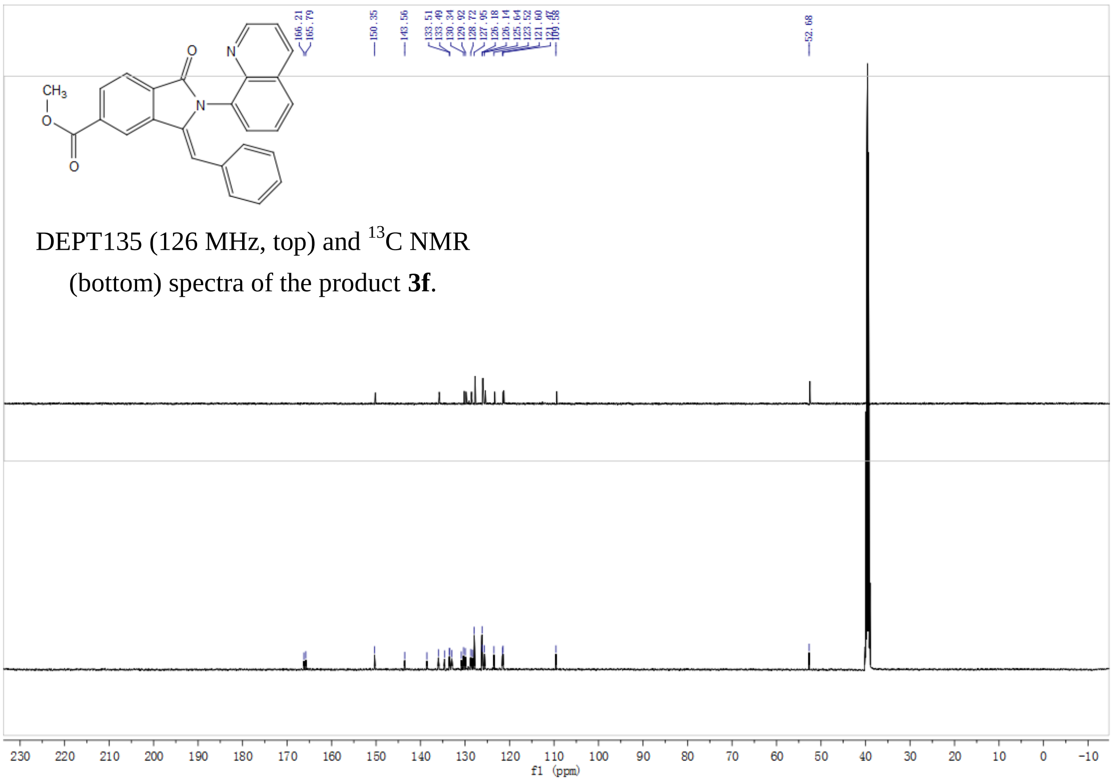

DEPT135 (126 MHz, top) and ^{13}C NMR (bottom) spectra of the product **3f**.

(*Z*)-3-Benzylidene-5-fluoro-2-(quinolin-8-yl)isoindolin-1-one (3g)

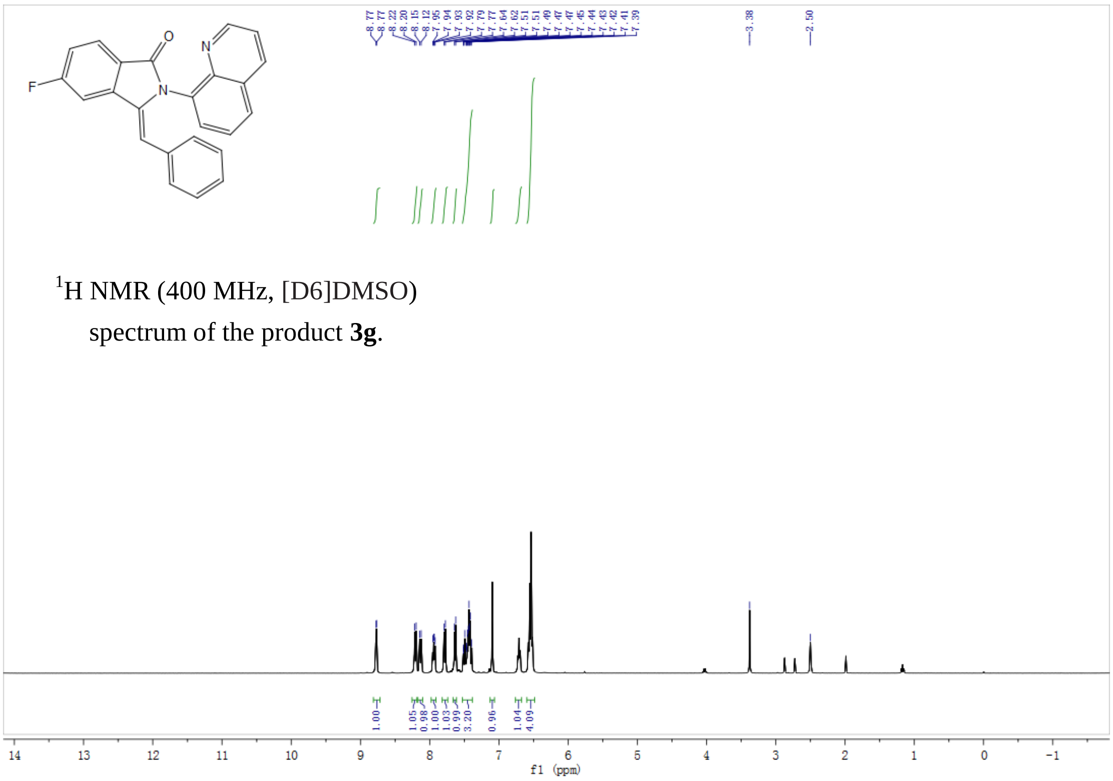

^{1}H NMR (400 MHz, [D6]DMSO) spectrum of the product **3g**.

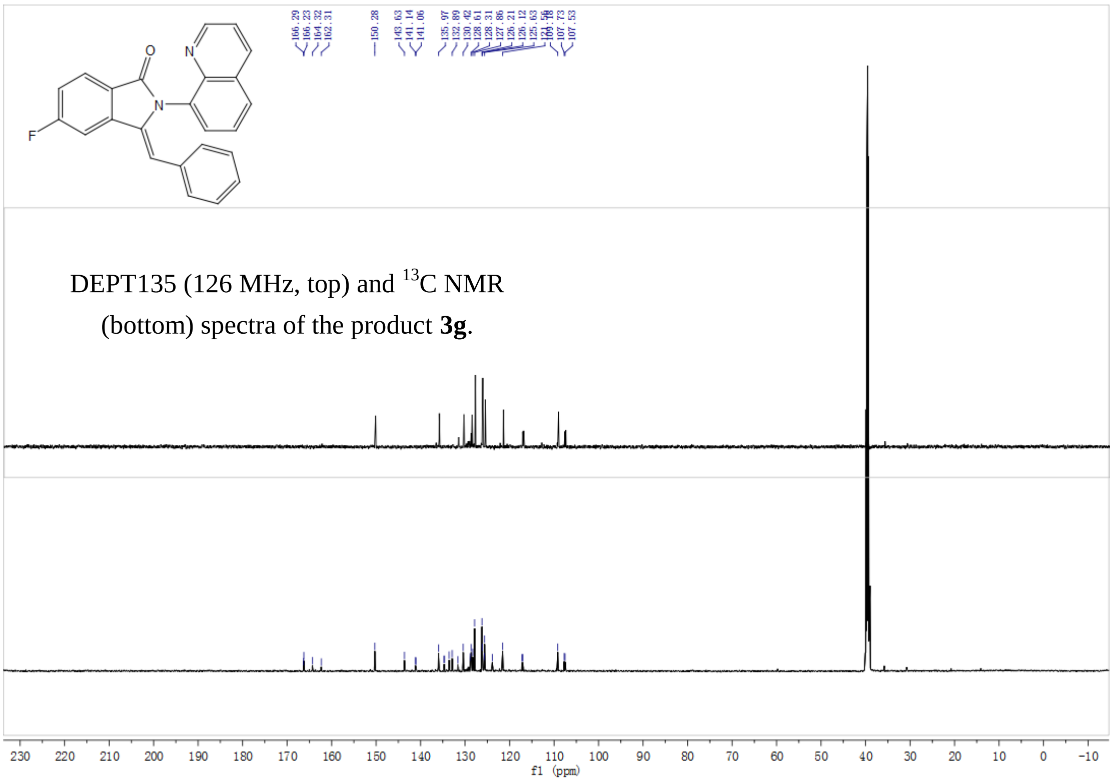

DEPT135 (126 MHz, top) and ^{13}C NMR (bottom) spectra of the product **3g**.

(*Z*)-3-Benzylidene-5-chloro-2-(quinolin-8-yl)isoindolin-1-one (3h)

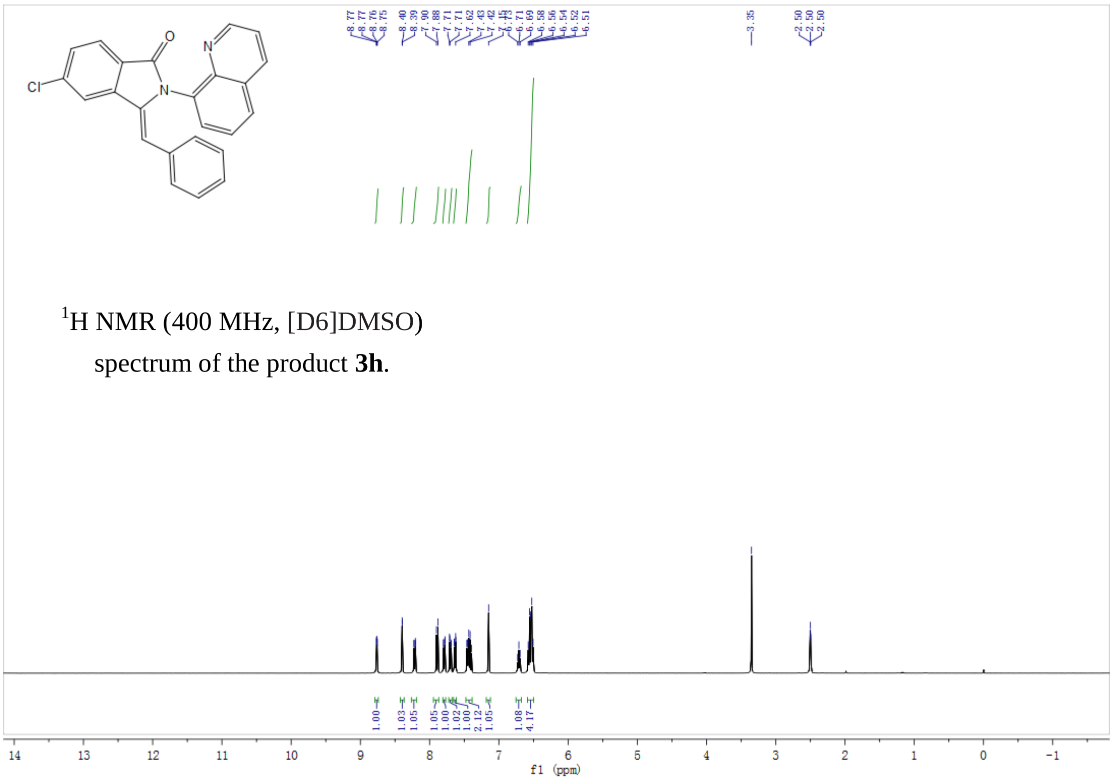

^{1}H NMR (400 MHz, [D6]DMSO) spectrum of the product **3h**.

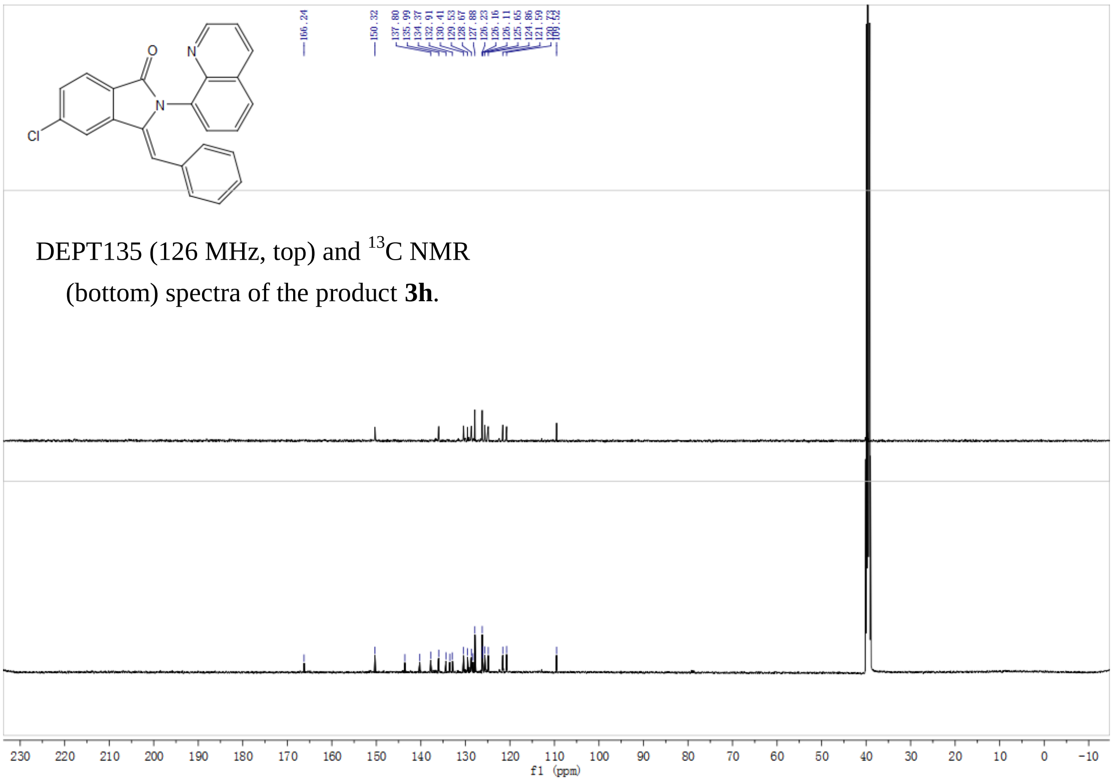

DEPT135 (126 MHz, top) and ^{13}C NMR (bottom) spectra of the product **3h**.

(*Z*)-3-Benzylidene-2-(quinolin-8-yl)-5-vinylisoindolin-1-one (3i)

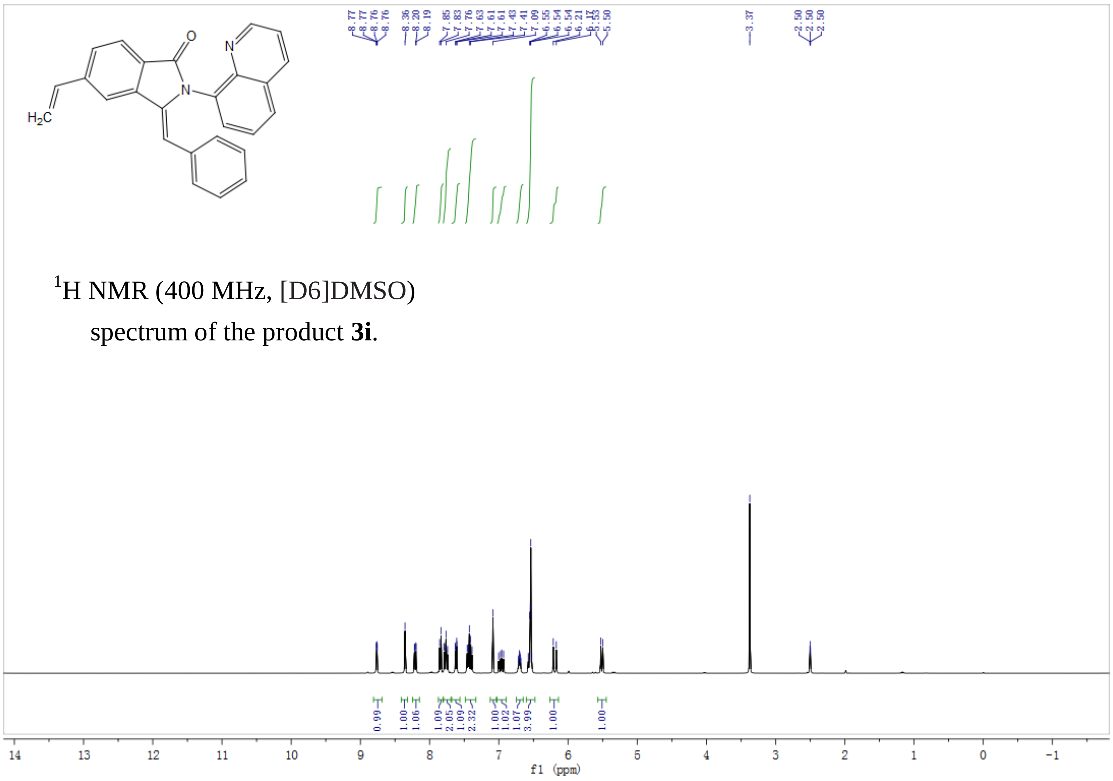

^{1}H NMR (400 MHz, [D6]DMSO) spectrum of the product **3i**.

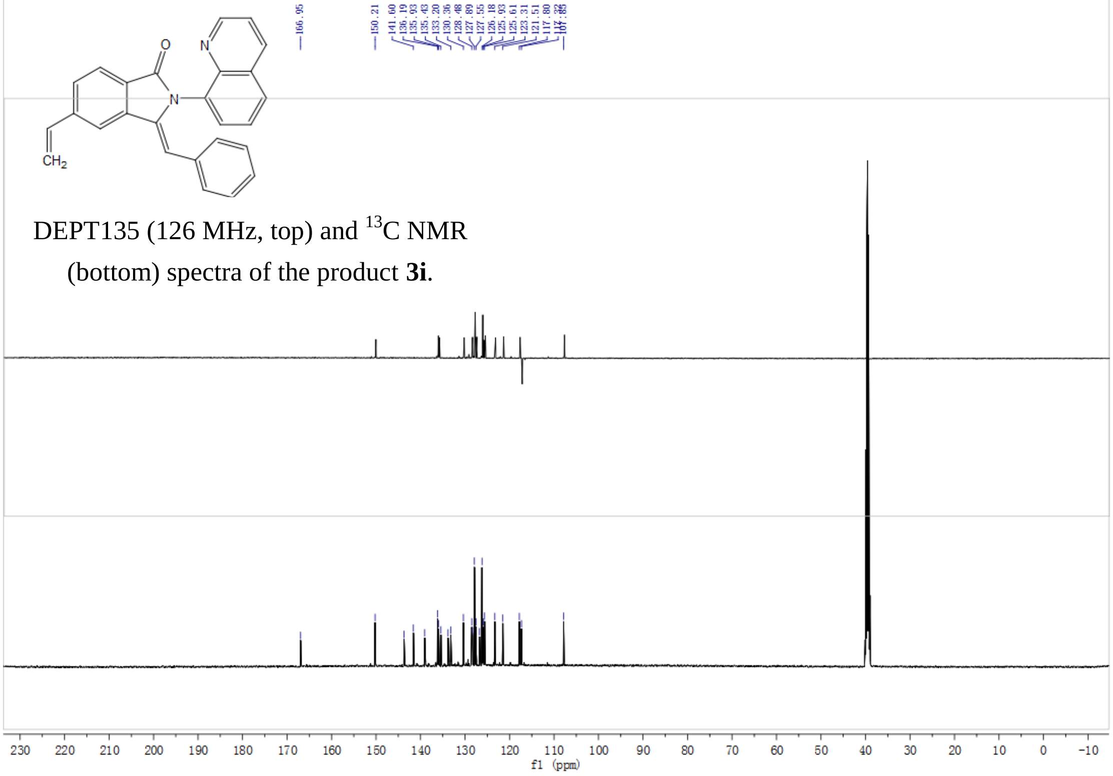

DEPT135 (126 MHz, top) and ^{13}C NMR (bottom) spectra of the product **3i**.

(*Z*)-3-Benzylidene-6-methyl-2-(quinolin-8-yl)isoindolin-1-one (3j)

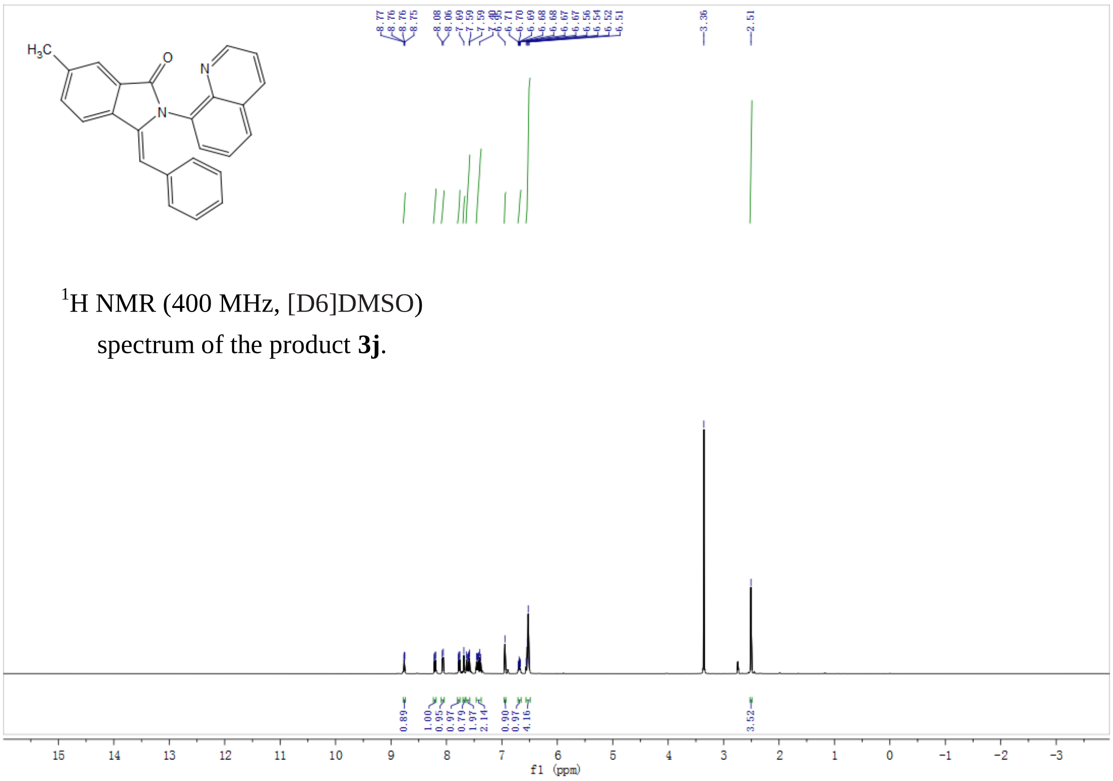

^{1}H NMR (400 MHz, [D6]DMSO) spectrum of the product **3j**.

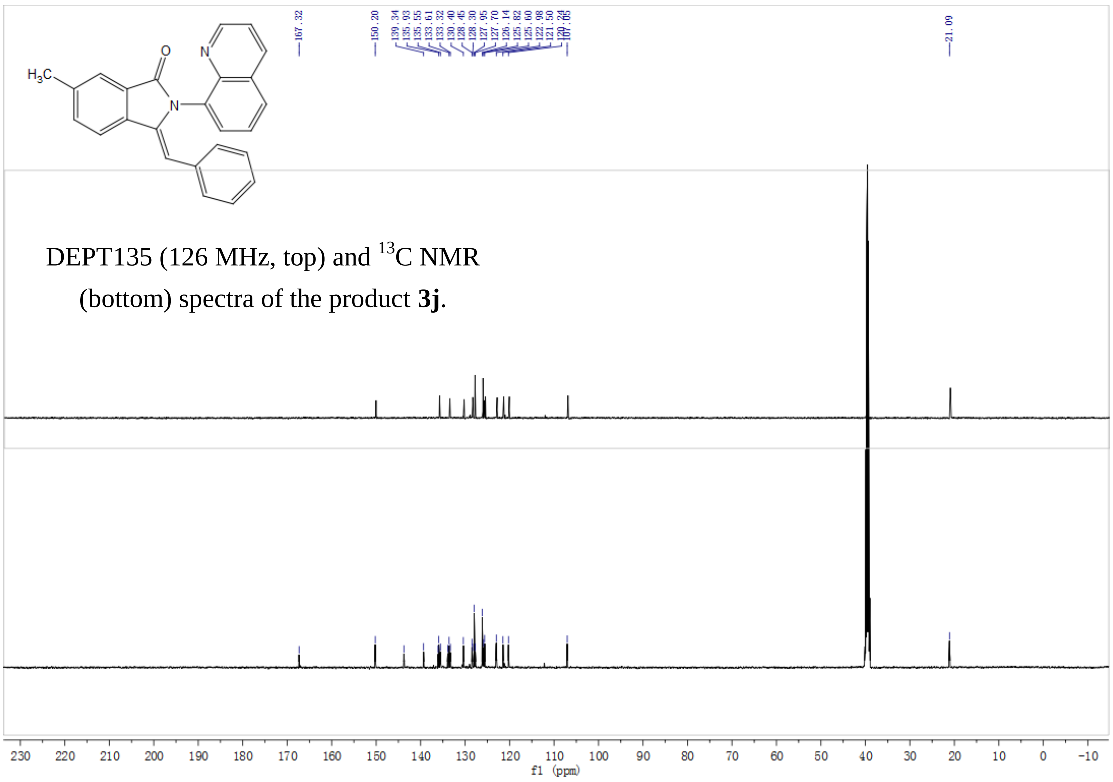

DEPT135 (126 MHz, top) and ^{13}C NMR (bottom) spectra of the product **3j**.

(*Z*)-3-Benzylidene-2-(quinolin-8-yl)-6-(trifluoromethyl)isoindolin-1-one (3k)

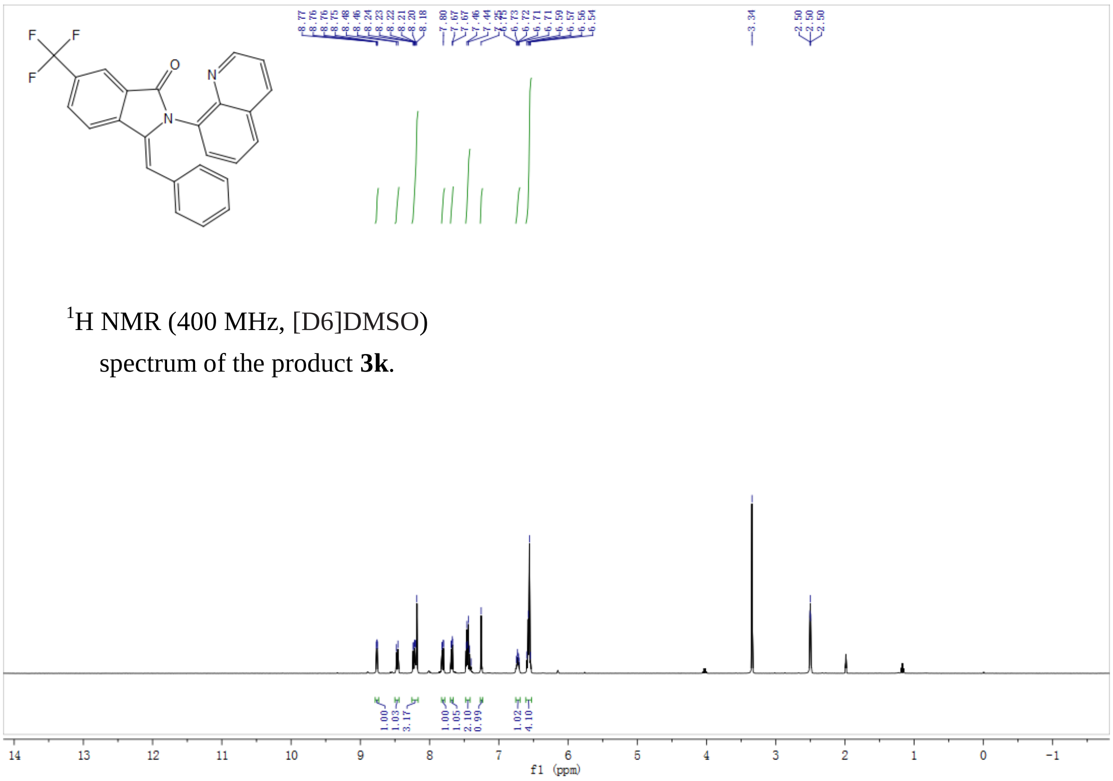

^{1}H NMR (400 MHz, [D6]DMSO) spectrum of the product **3k**.

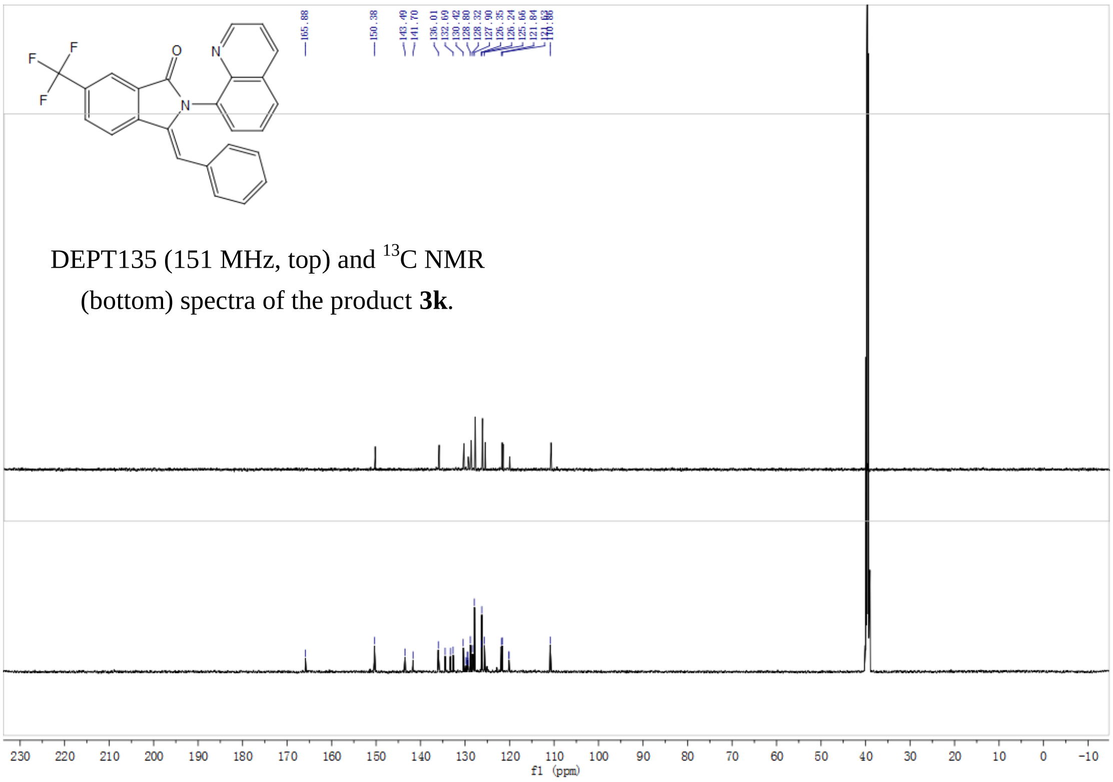

DEPT135 (151 MHz, top) and ^{13}C NMR (bottom) spectra of the product **3k**.

(*Z*)-3-Benzylidene-7-methyl-2-(quinolin-8-yl)isoindolin-1-one (3l).

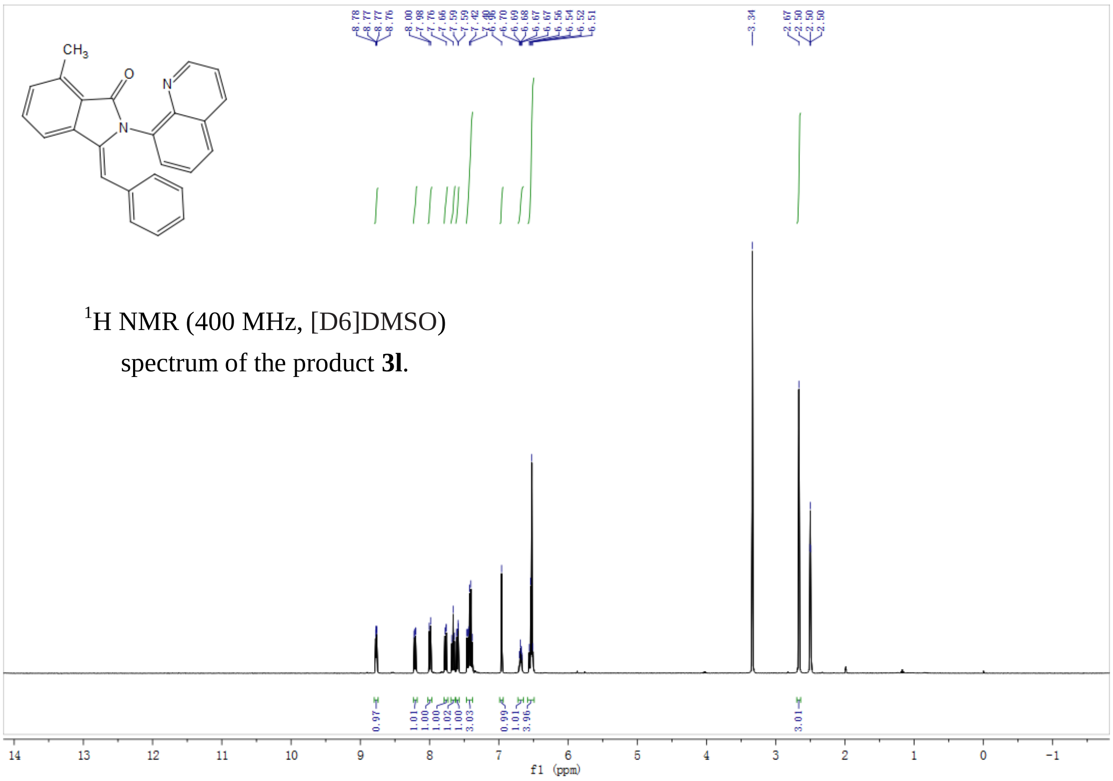

^{1}H NMR (400 MHz, [D6]DMSO) spectrum of the product **3l**.

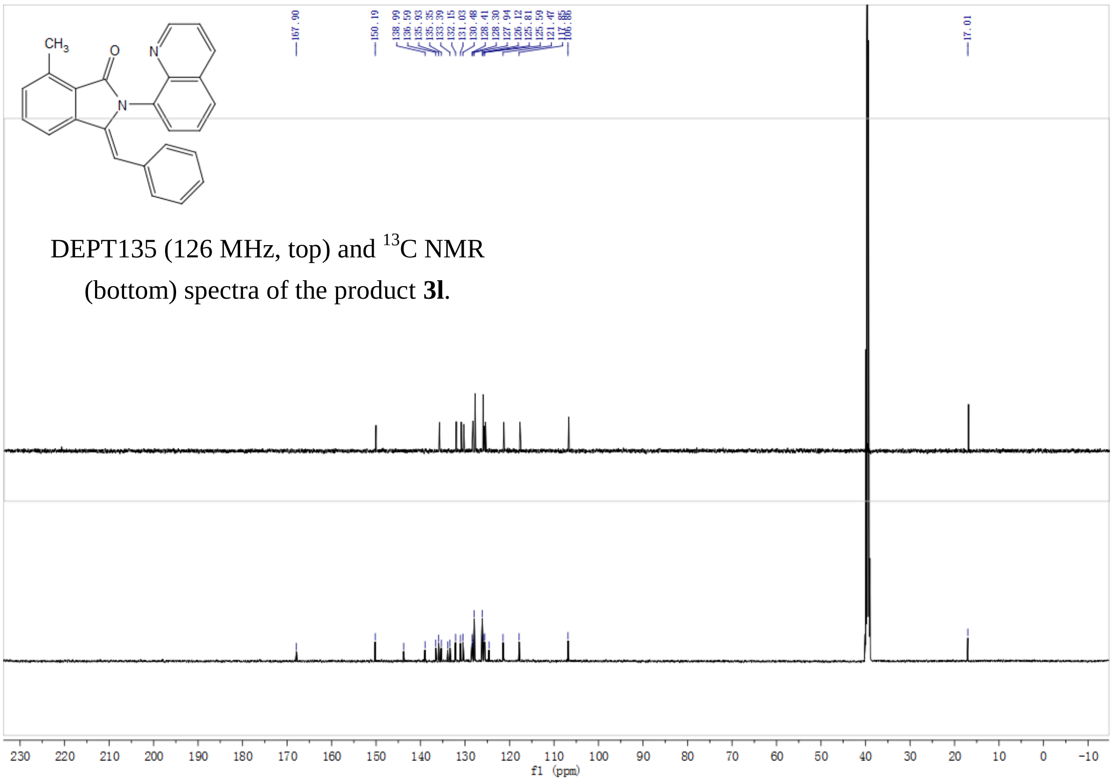

DEPT135 (126 MHz, top) and ^{13}C NMR (bottom) spectra of the product **3l**.

(*Z*)-3-Benzylidene-2-(quinolin-8-yl)-2,3,6,7,8,9-hexahydro-1*H*-benzo[*e*]isoindol-1-one (3m)

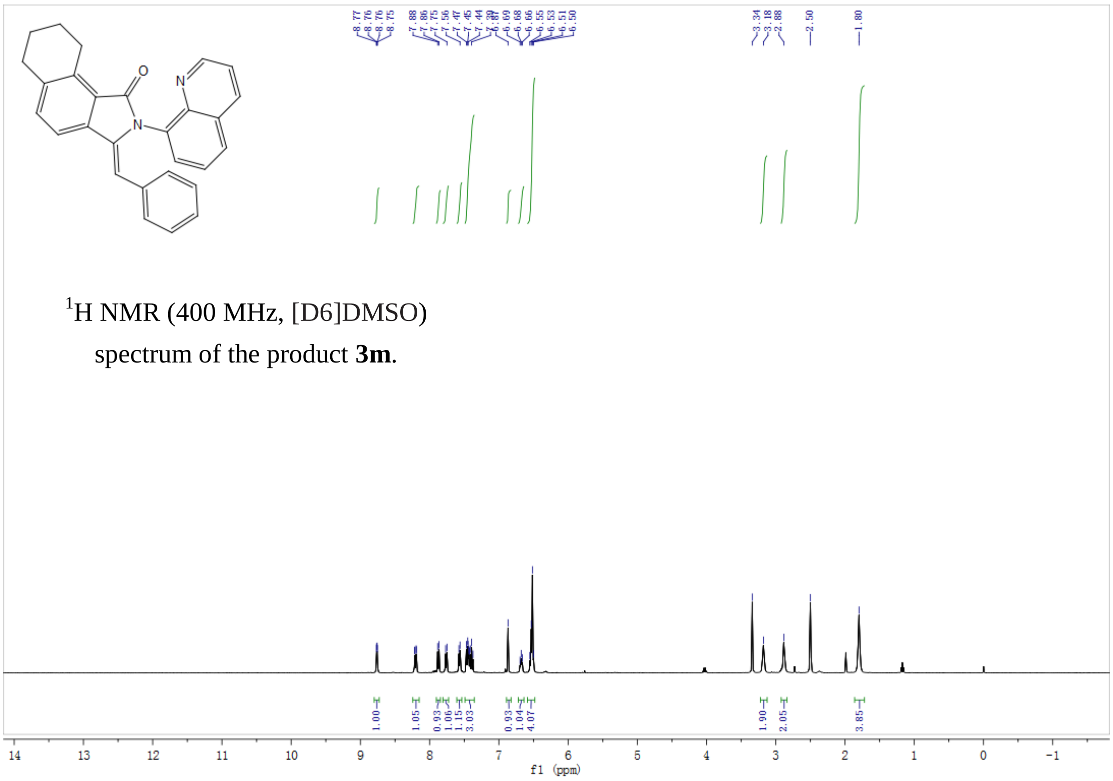

^{1}H NMR (400 MHz, [D6]DMSO) spectrum of the product **3m**.

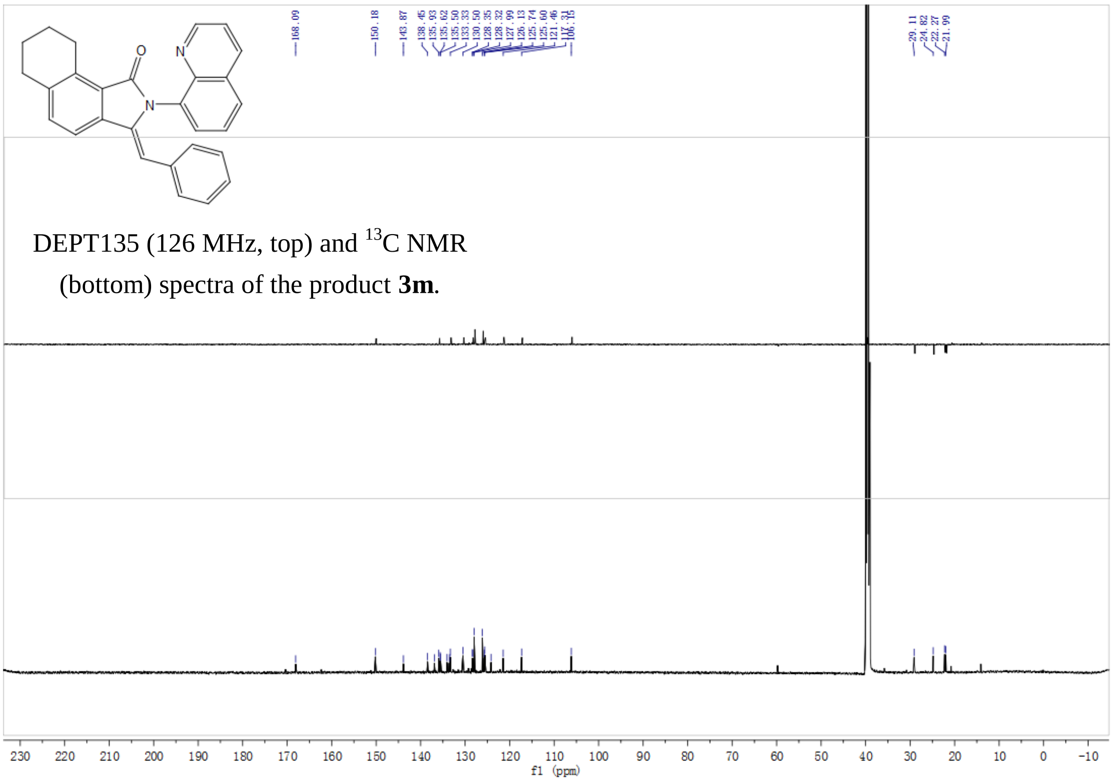

DEPT135 (126 MHz, top) and ^{13}C NMR (bottom) spectra of the product **3m**.

(*Z*)-3-(4-Methylbenzylidene)-2-(quinolin-8-yl)isoindolin-1-one (3n)

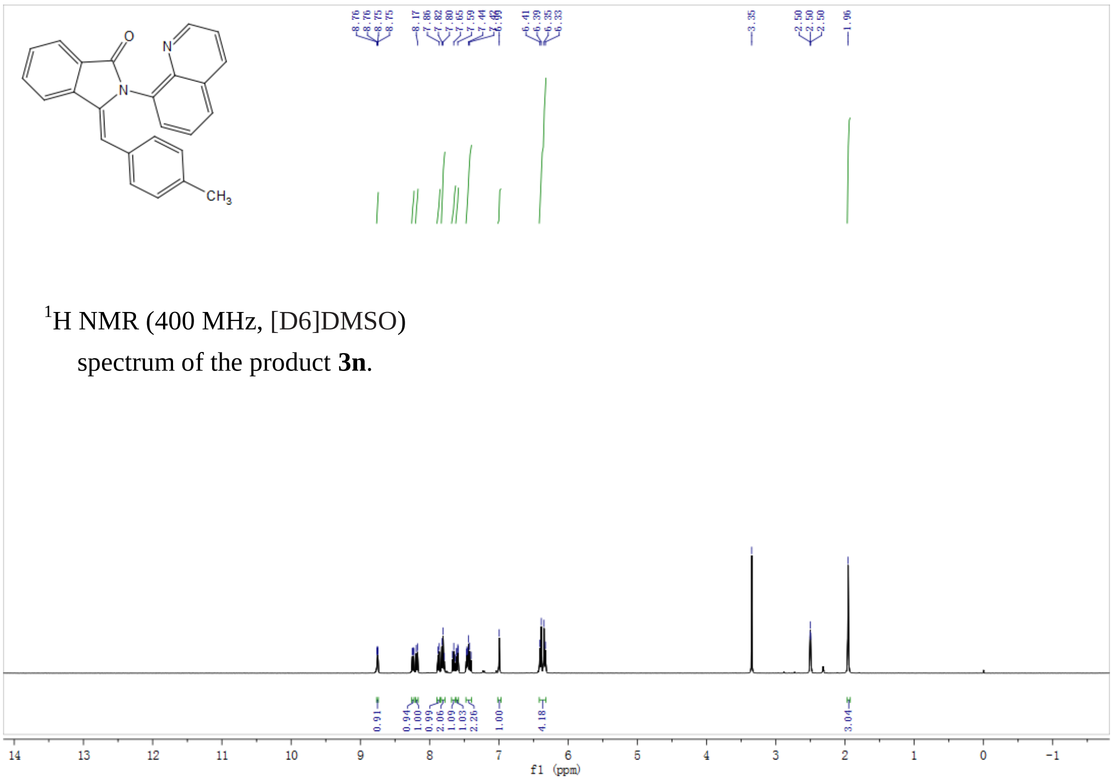

^{1}H NMR (400 MHz, [D6]DMSO) spectrum of the product **3n**.

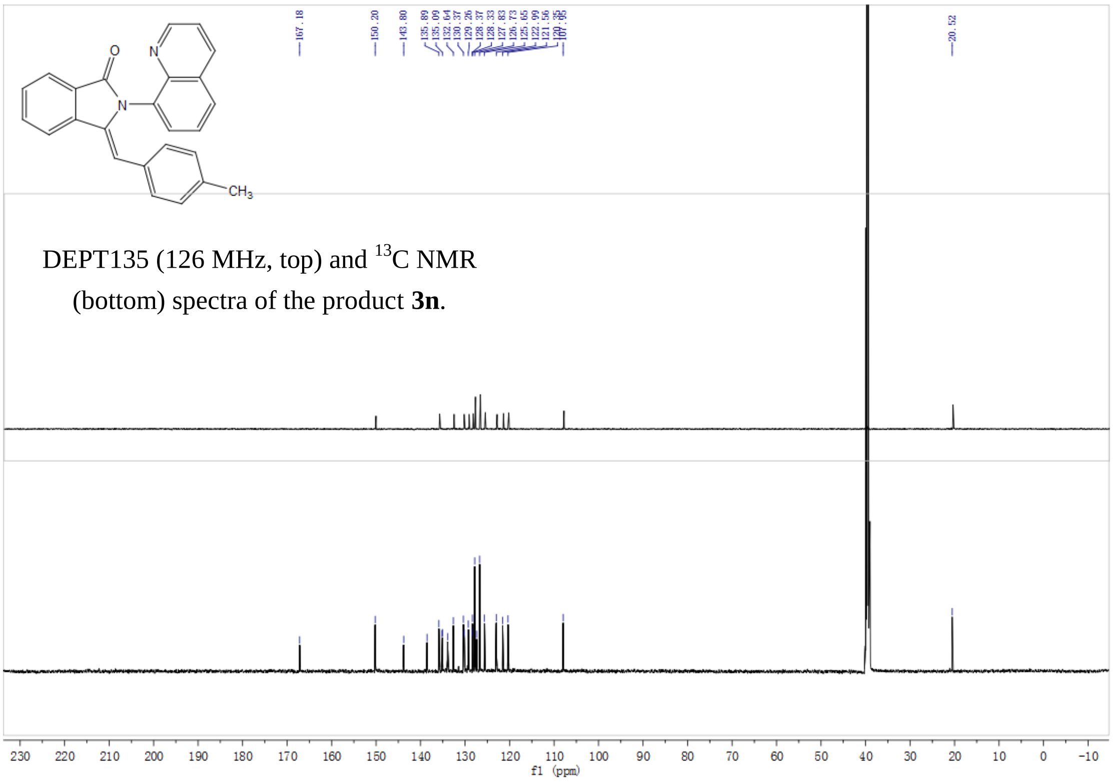

DEPT135 (126 MHz, top) and ^{13}C NMR (bottom) spectra of the product **3n**.

(*Z*)-3-(4-Chlorobenzylidene)-2-(quinolin-8-yl)isoindolin-1-one (3o)

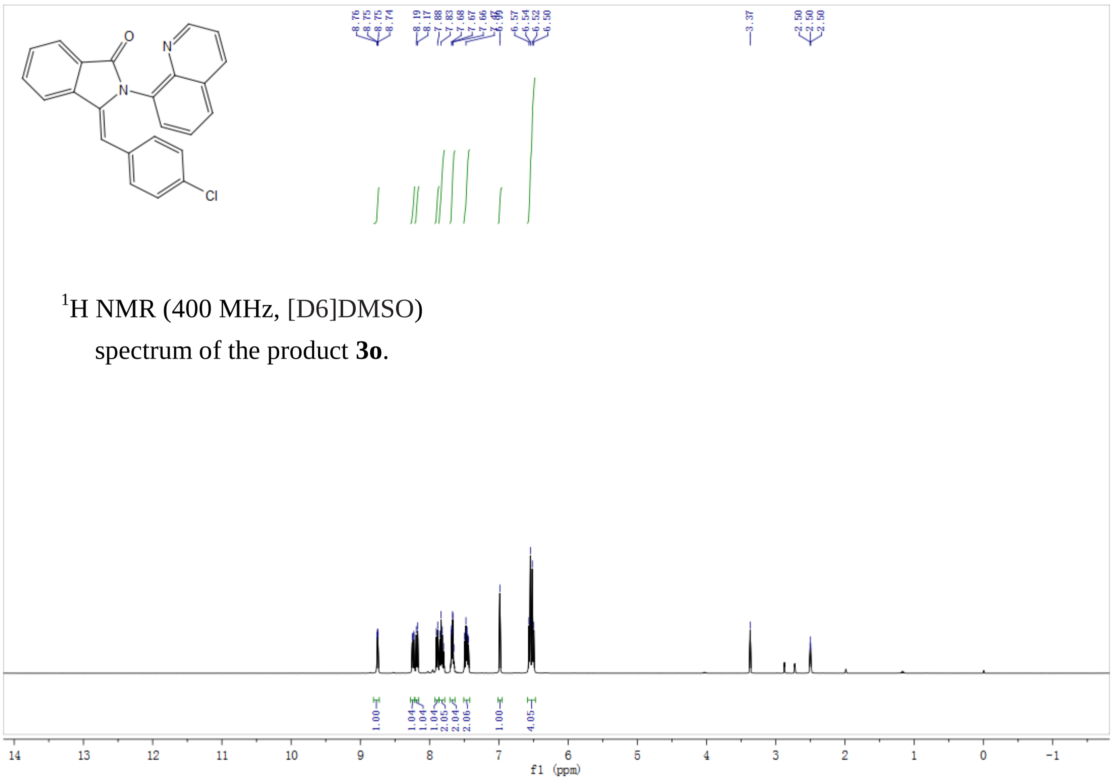

^{1}H NMR (400 MHz, [D6]DMSO) spectrum of the product **3o**.

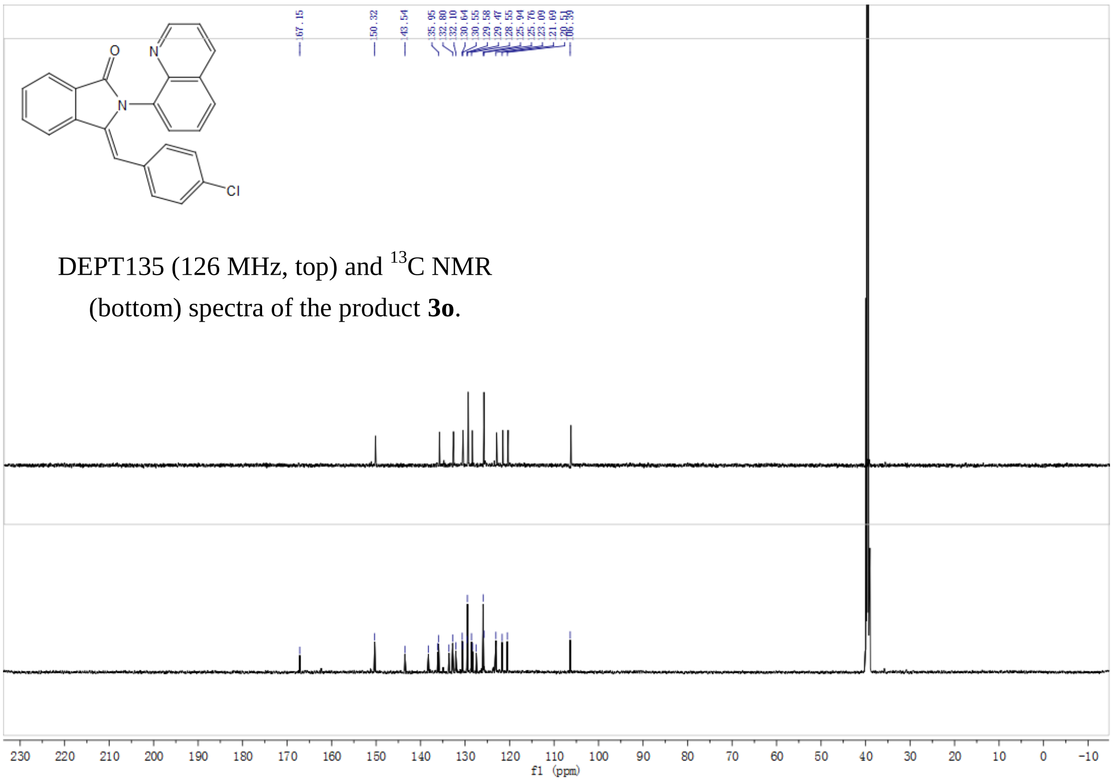

DEPT135 (126 MHz, top) and ^{13}C NMR (bottom) spectra of the product **3o**.

(*Z*)-3-(4-Bromobenzylidene)-2-(quinolin-8-yl)isoindolin-1-one (3p)

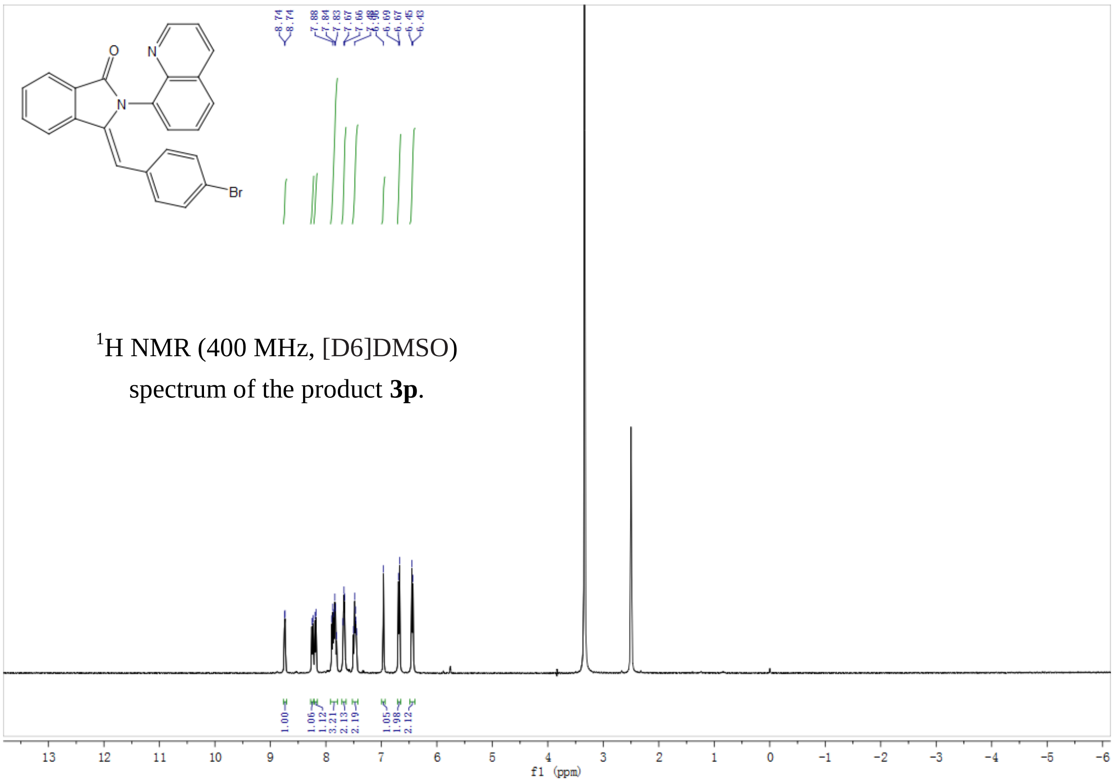

^{1}H NMR (400 MHz, [D6]DMSO) spectrum of the product **3p**.

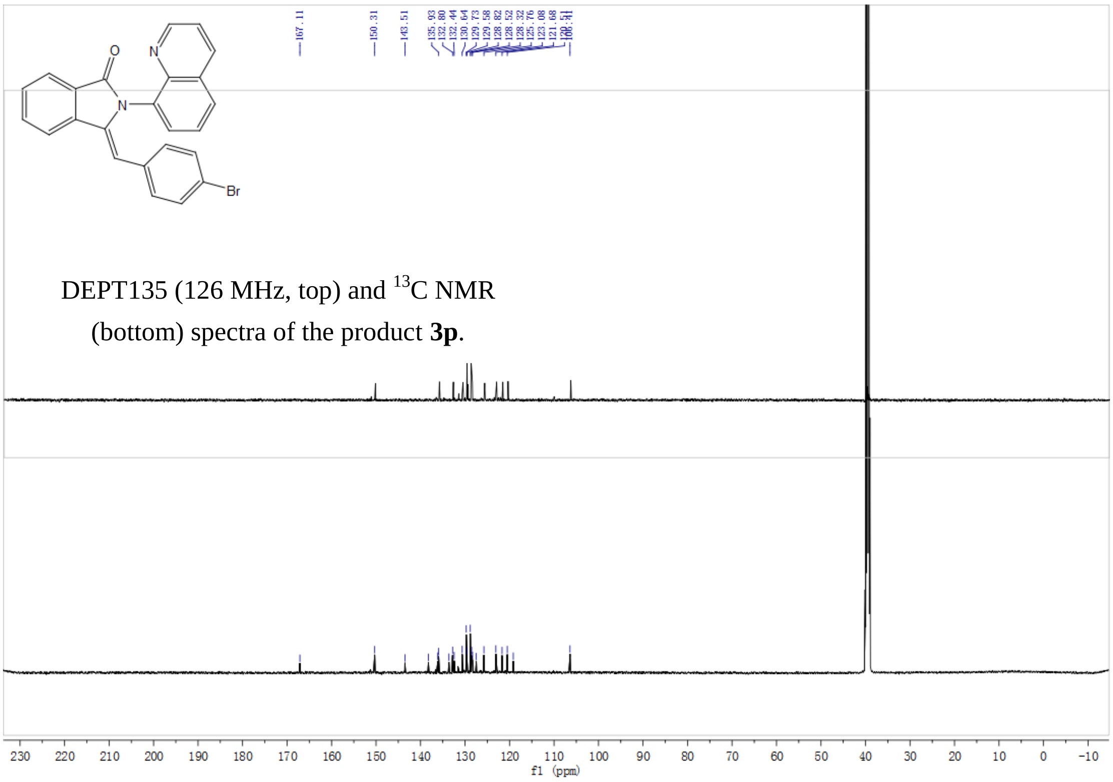

DEPT135 (126 MHz, top) and ^{13}C NMR (bottom) spectra of the product **3p**.

(*Z*)-3-(4-Bromobenzylidene)-2-(quinolin-8-yl)isoindolin-1-one (3q)

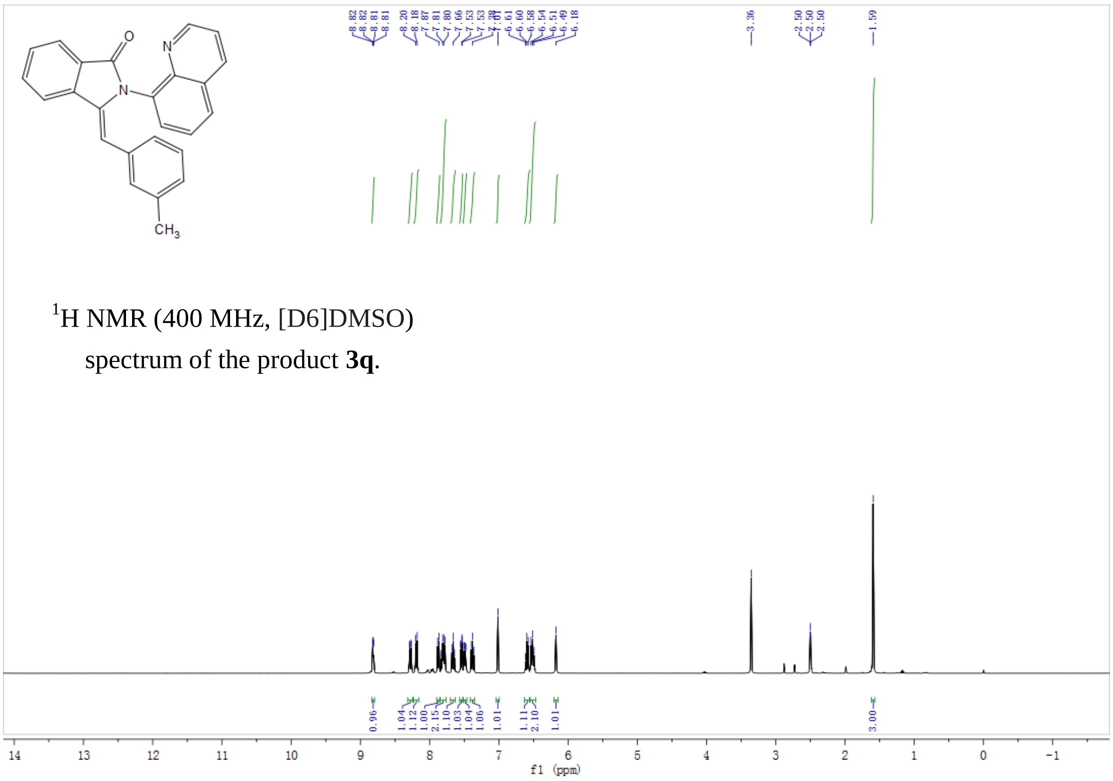

^{1}H NMR (400 MHz, [D6]DMSO) spectrum of the product **3q**.

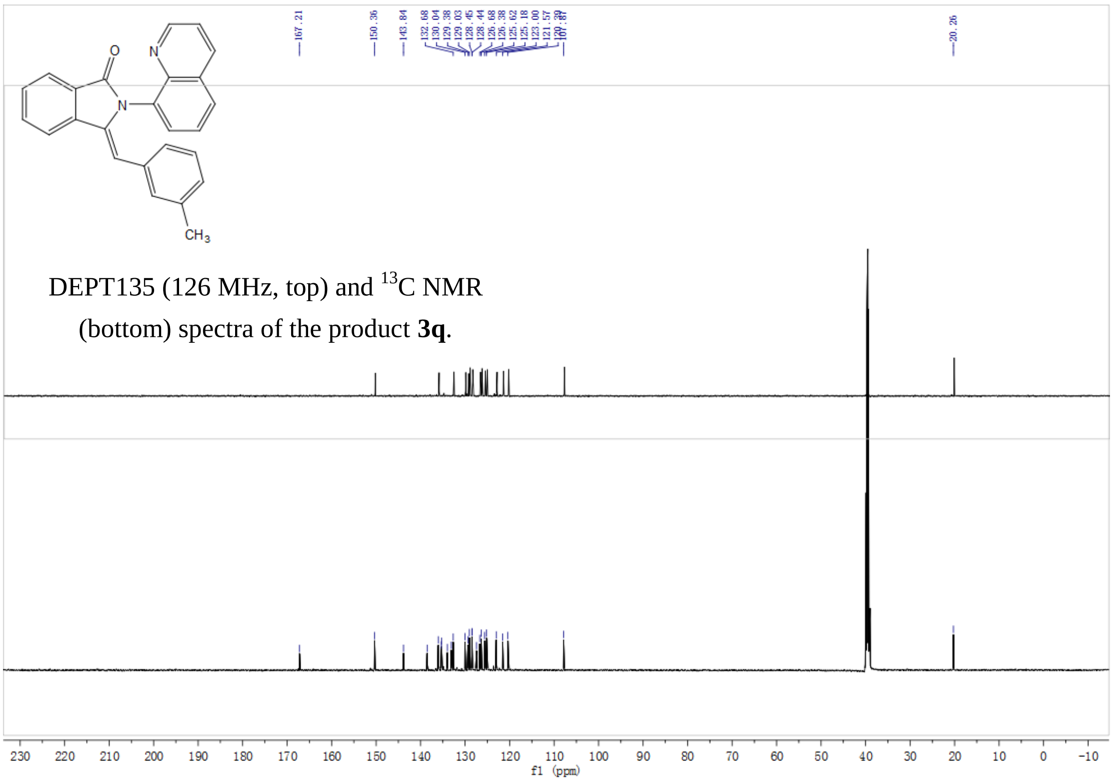

DEPT135 (126 MHz, top) and ^{13}C NMR (bottom) spectra of the product **3q**.

(*Z*)-3-(3-Chlorobenzylidene)-2-(quinolin-8-yl)isoindolin-1-one (3r)

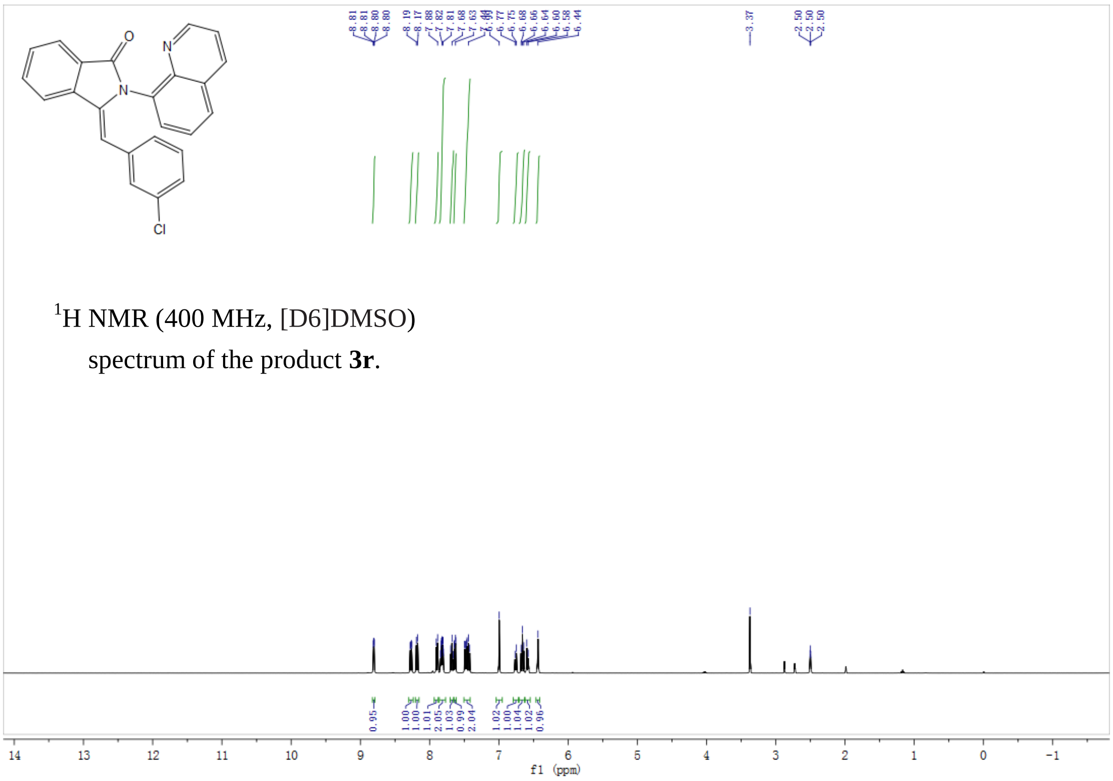

^{1}H NMR (400 MHz, [D6]DMSO) spectrum of the product **3r**.

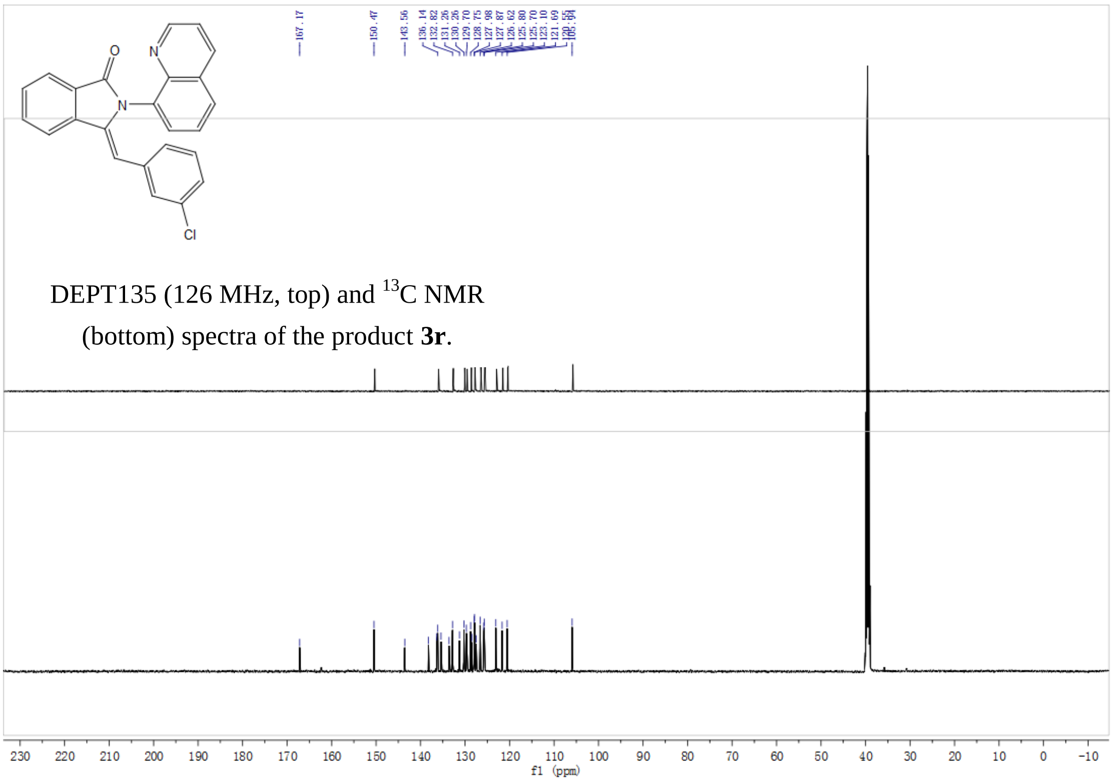

DEPT135 (126 MHz, top) and ^{13}C NMR (bottom) spectra of the product **3r**.

(*Z*)-2-Fluoro-5-((3-oxo-2-(quinolin-8-yl)isoindolin-1-ylidene)methyl)benzonitrile (3s)

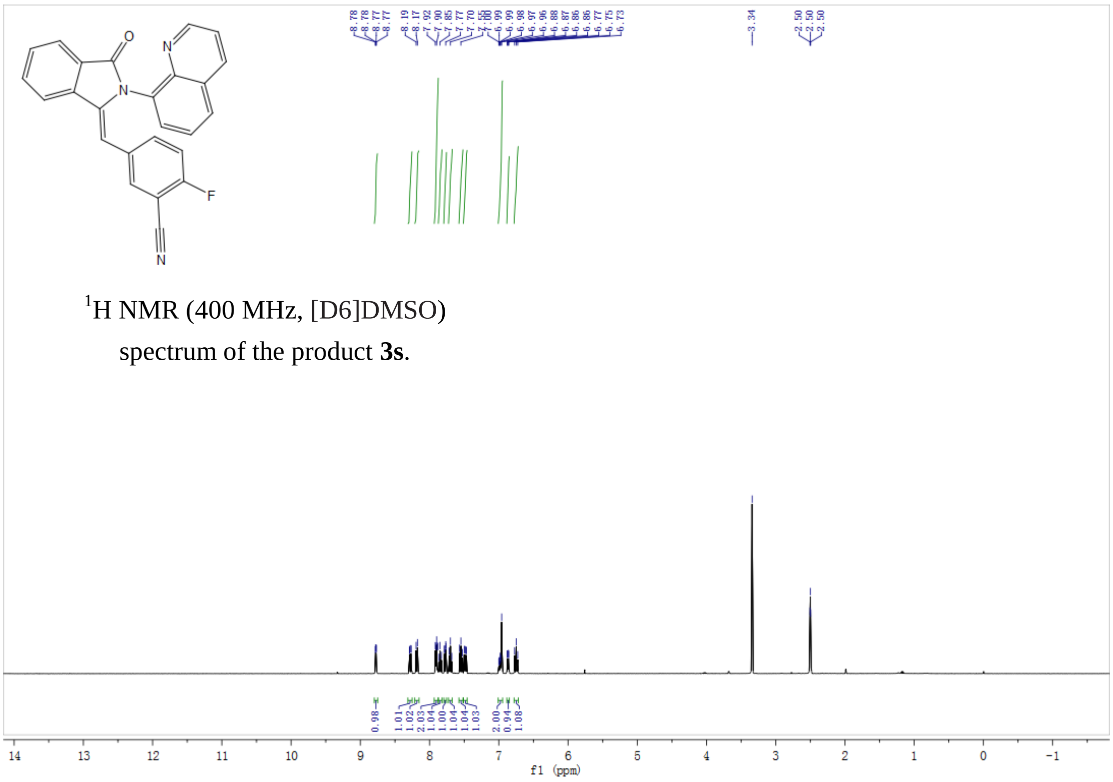

^{1}H NMR (400 MHz, [D6]DMSO) spectrum of the product **3s**.

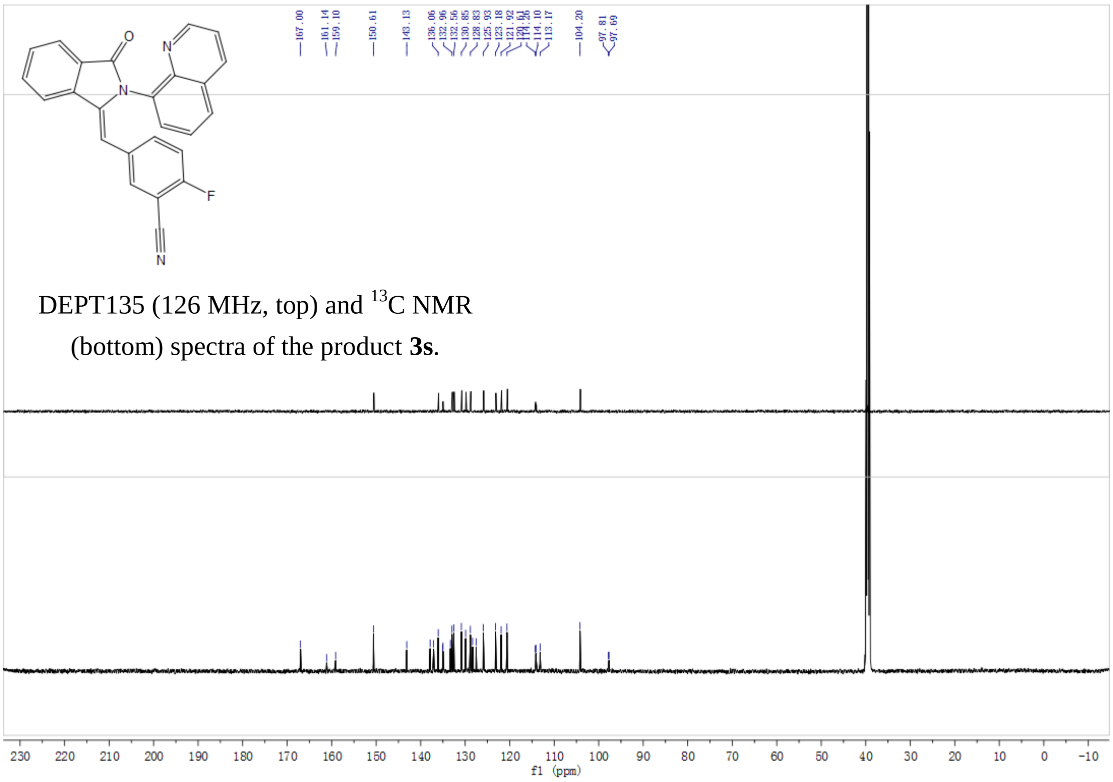

DEPT135 (126 MHz, top) and ^{13}C NMR (bottom) spectra of the product **3s**.

(*Z*)-3-(4-Fluoro-3-methylbenzylidene)-2-(quinolin-8-yl)isoindolin-1-one (3t)

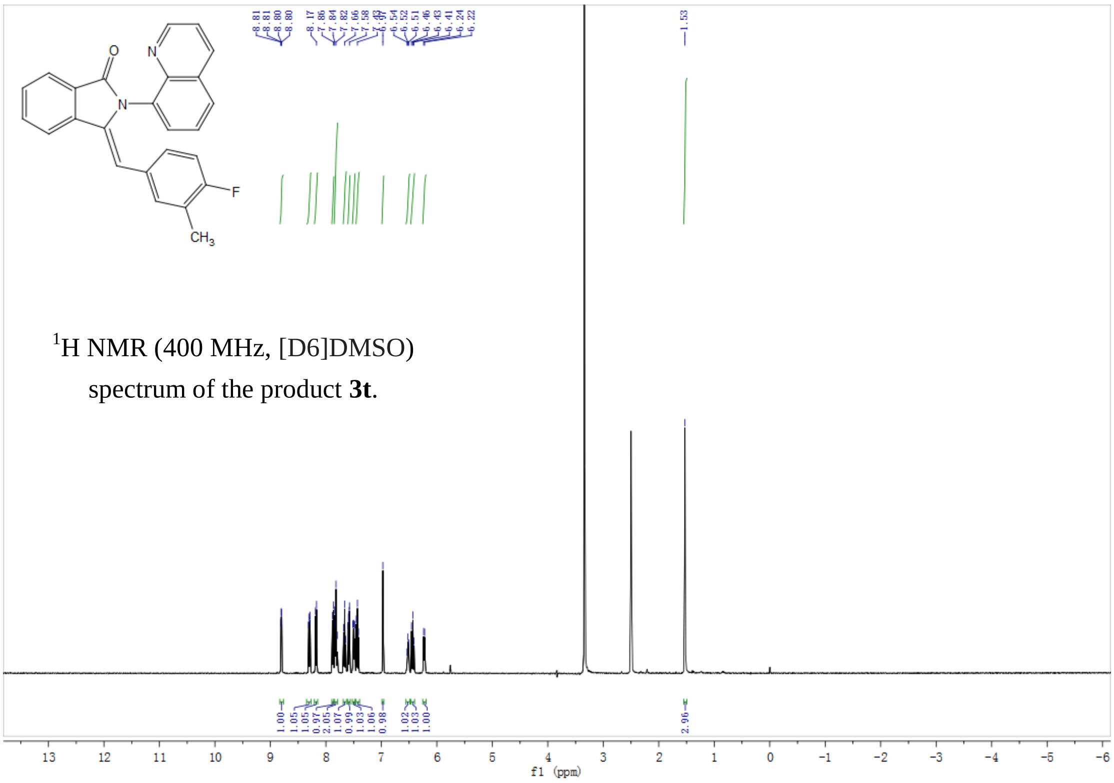

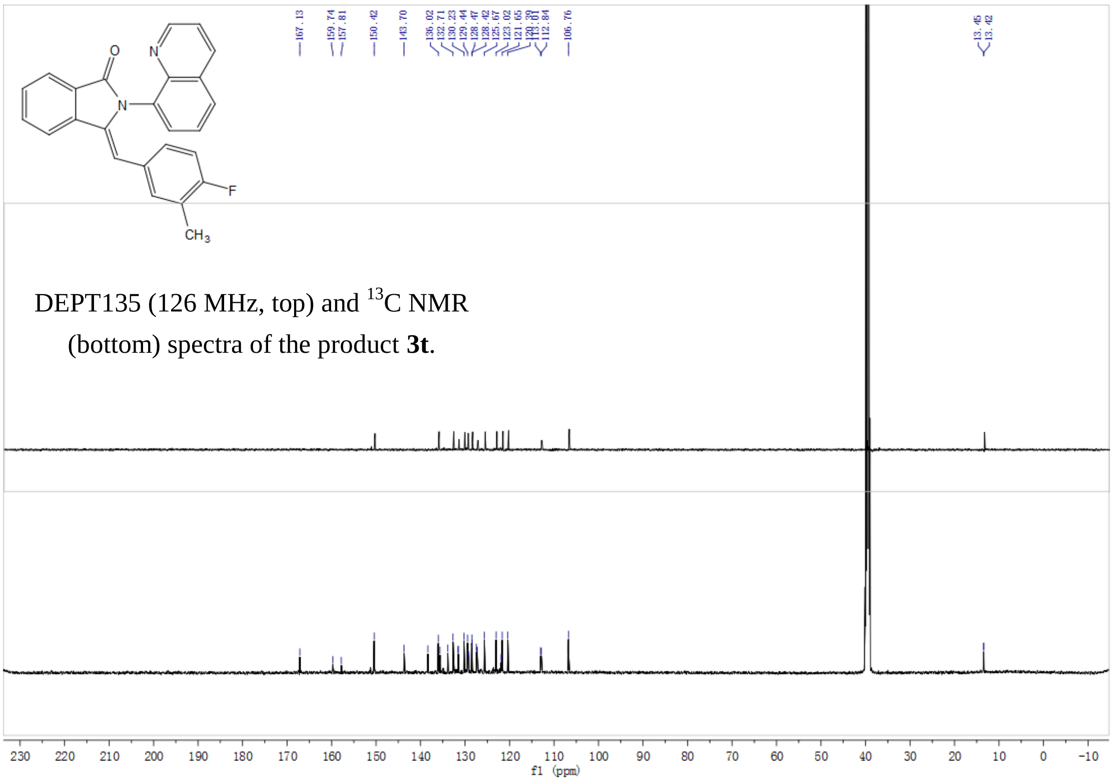

(*Z*)-3-(Cyclopropylmethylene)-2-(quinolin-8-yl)isoindolin-1-one (3u)

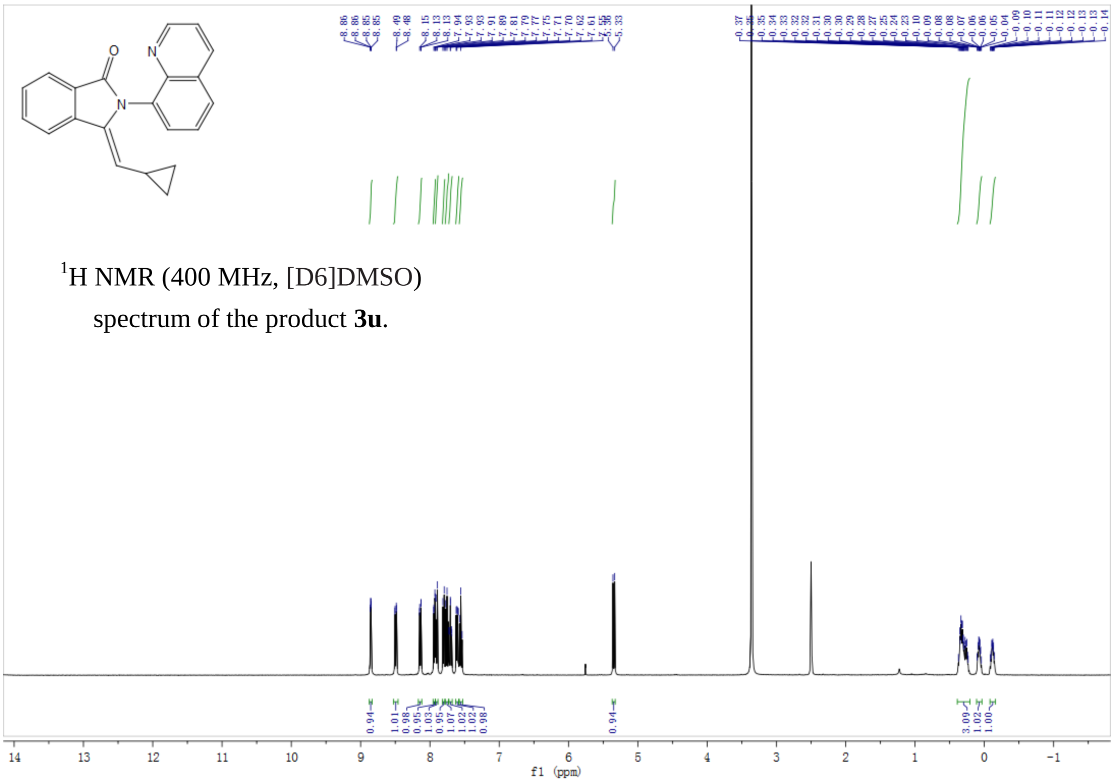

^{1}H NMR (400 MHz, [D6]DMSO) spectrum of the product **3u**.

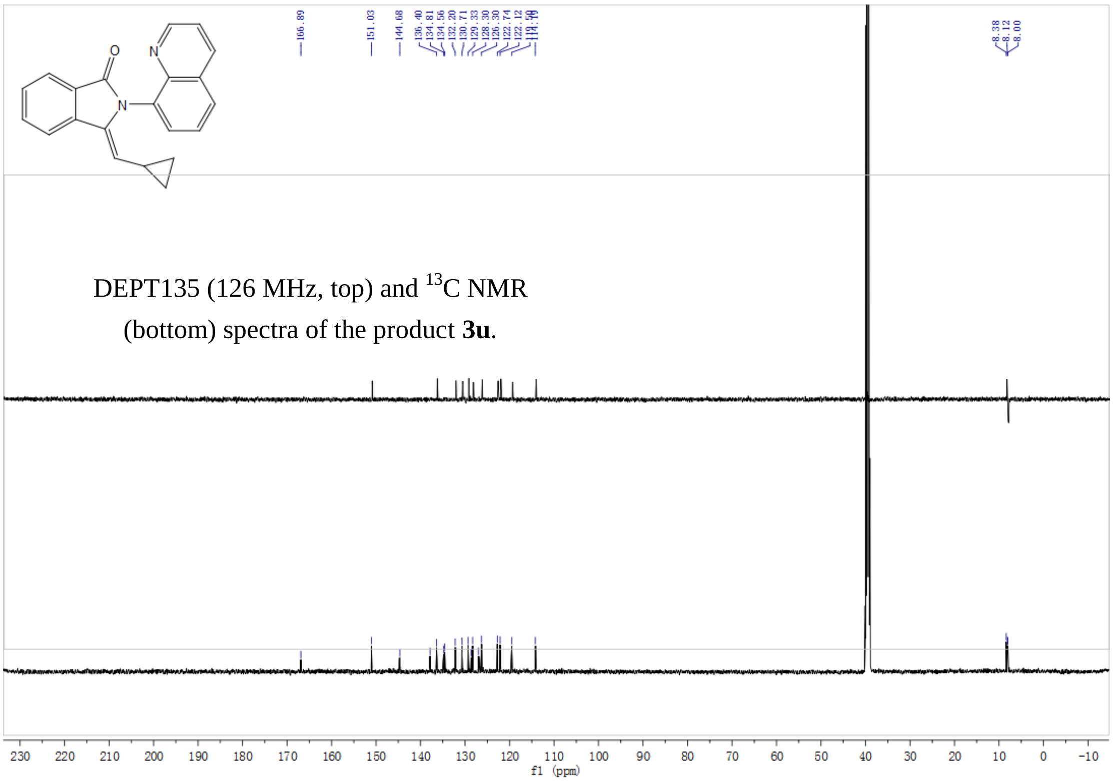

DEPT135 (126 MHz, top) and ^{13}C NMR (bottom) spectra of the product **3u**.

(*Z*)-Ethyl 2-(3-oxo-2-(quinolin-8-yl)isoindolin-1-ylidene)acetate (3v)

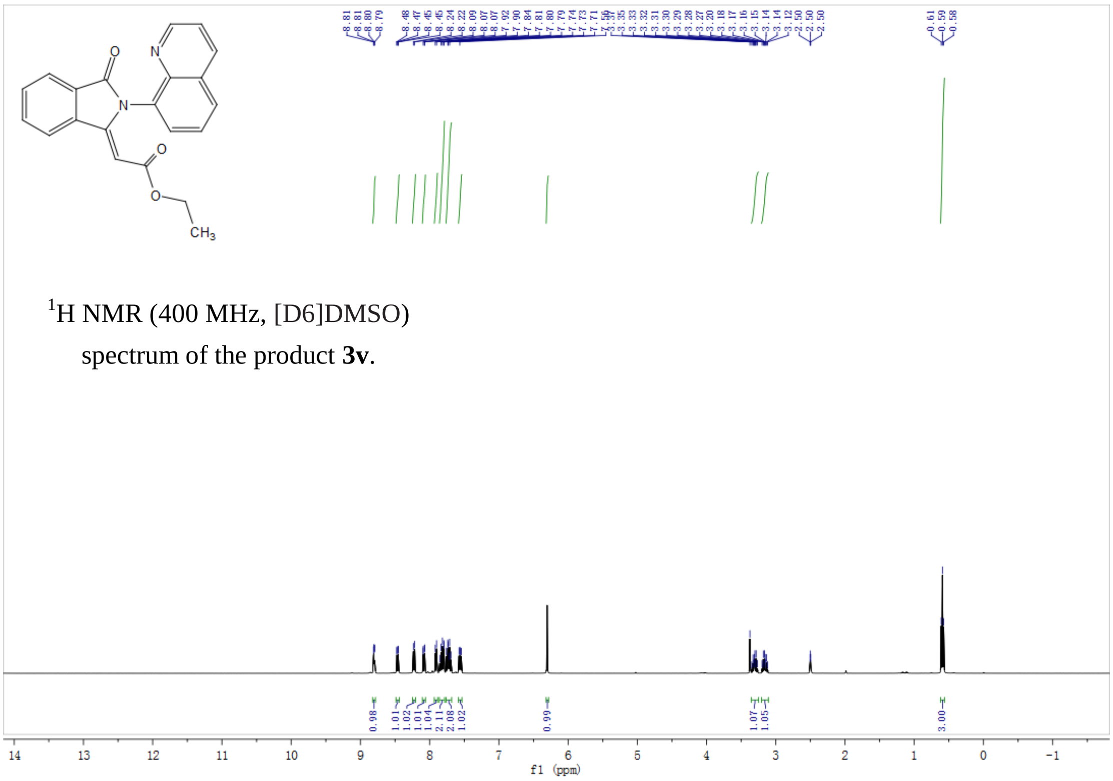

^{1}H NMR (400 MHz, [D6]DMSO) spectrum of the product **3v**.

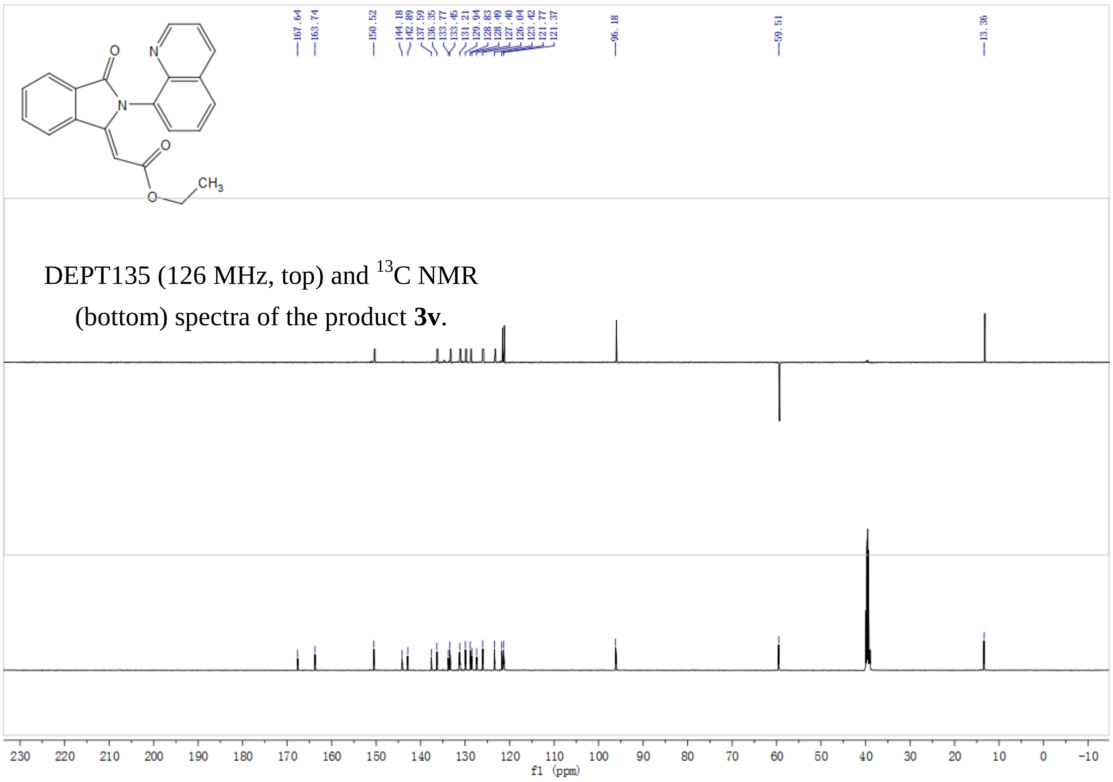

DEPT135 (126 MHz, top) and ^{13}C NMR (bottom) spectra of the product **3v**.

(*Z*)-*tert*-Butyl 2-(3-oxo-2-(quinolin-8-yl)isoindolin-1-ylidene)acetate (3w)

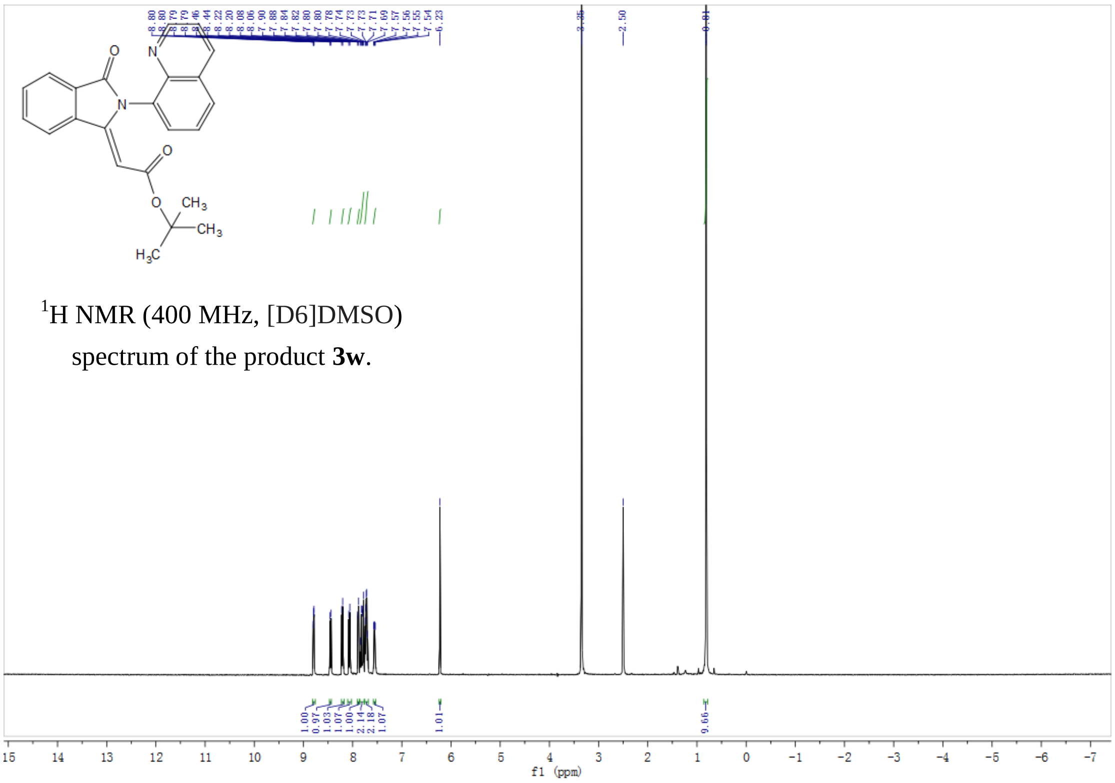

^{1}H NMR (400 MHz, [D6]DMSO) spectrum of the product **3w**.

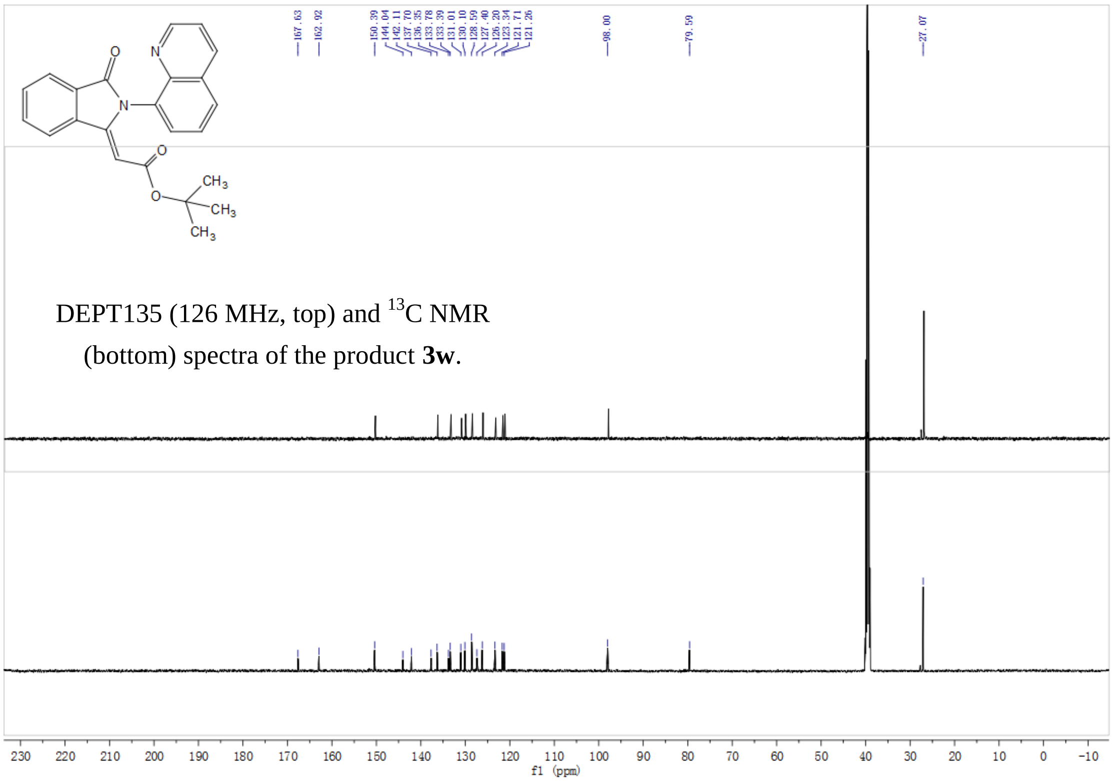

DEPT135 (126 MHz, top) and ^{13}C NMR (bottom) spectra of the product **3w**.

4-Benzylphthalazin-1(2*H*)-one (4a)

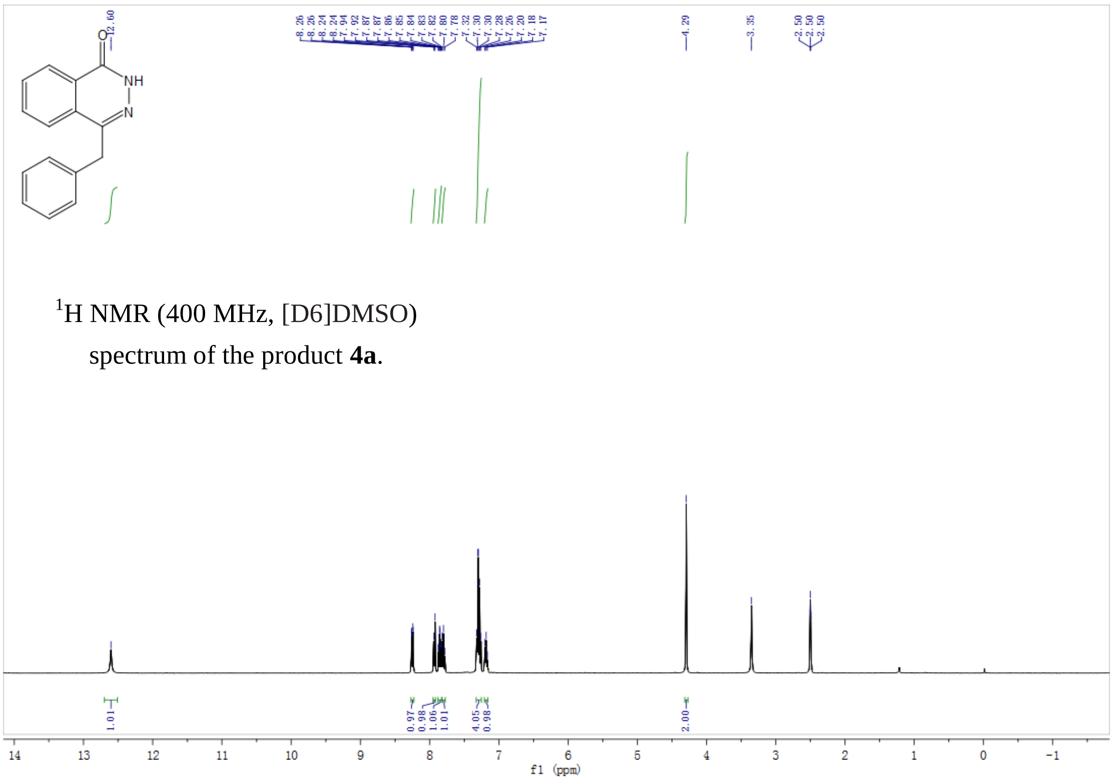

^{1}H NMR (400 MHz, [D6]DMSO) spectrum of the product **4a**.

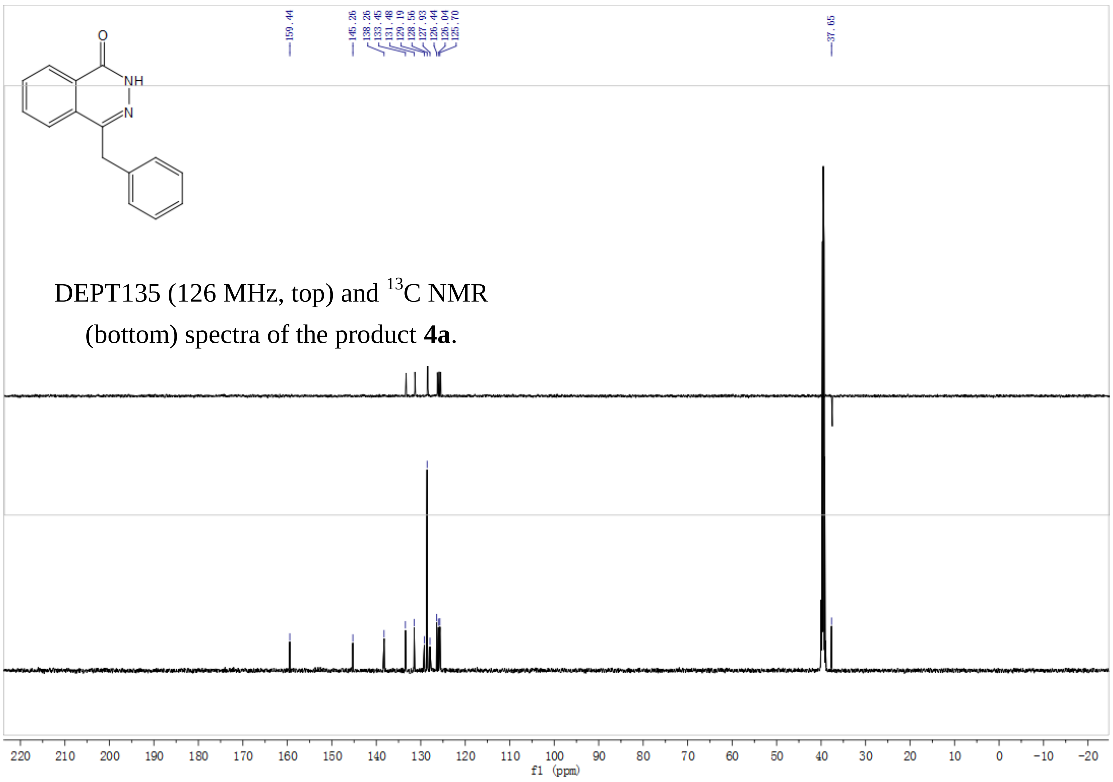

DEPT135 (126 MHz, top) and ^{13}C NMR (bottom) spectra of the product **4a**.

4-Benzyl-6-methylphthalazin-1(2*H*)-one (4b)

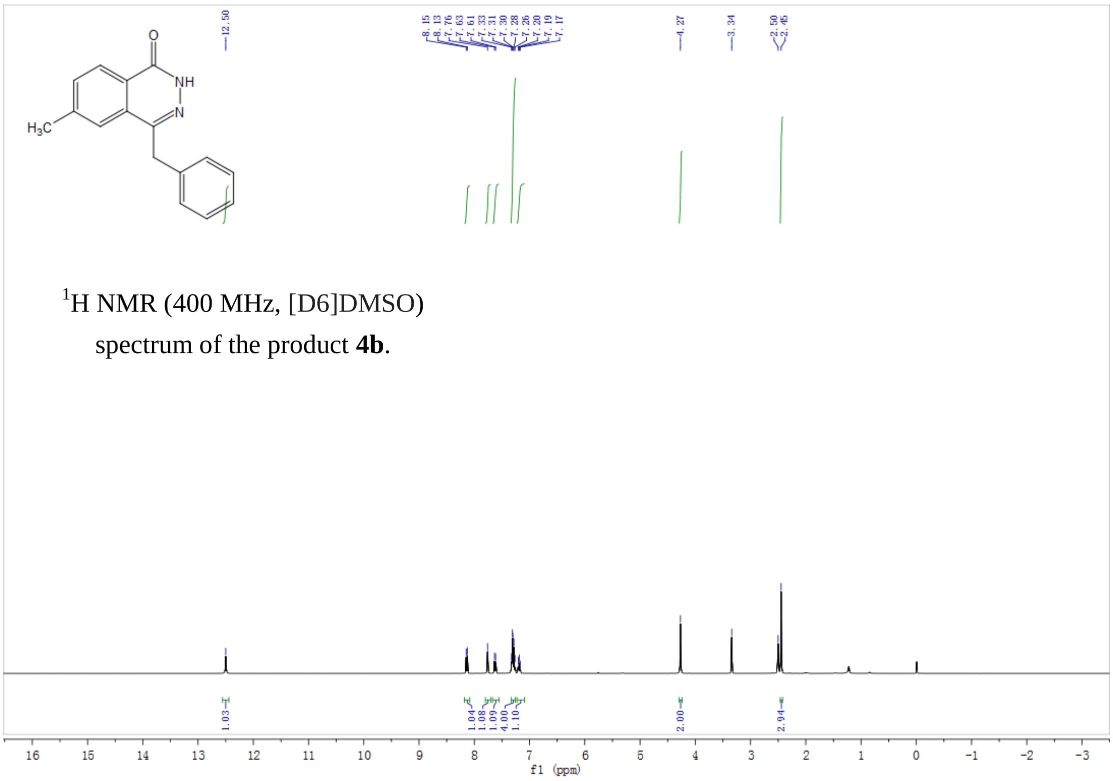

^{1}H NMR (400 MHz, [D6]DMSO) spectrum of the product **4b**.

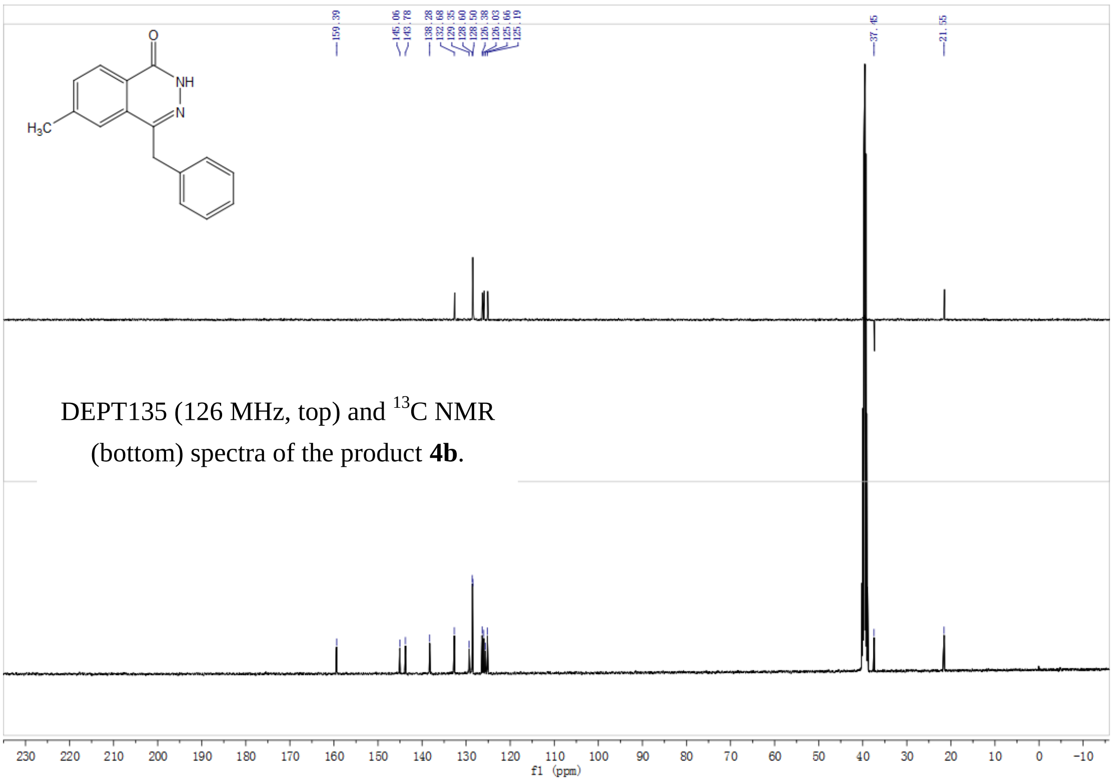

DEPT135 (126 MHz, top) and ^{13}C NMR (bottom) spectra of the product **4b**.

4-Benzyl-7-(trifluoromethyl)phthalazin-1(2*H*)-one (4k)

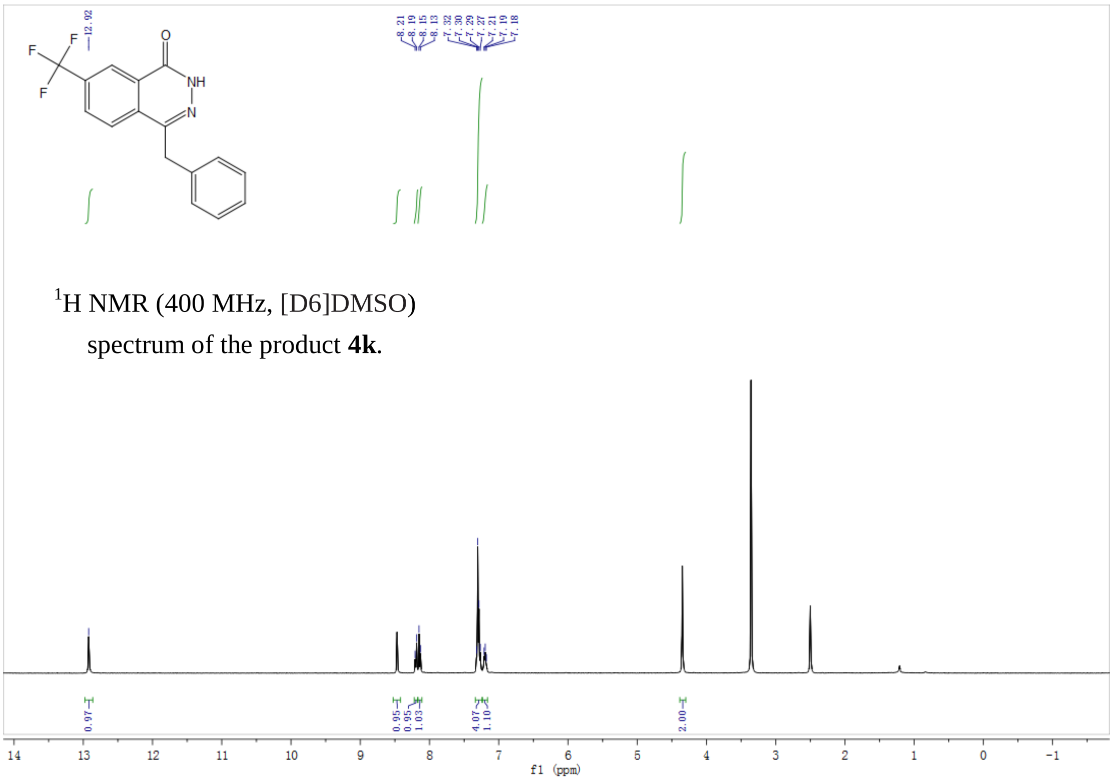

^{1}H NMR (400 MHz, [D6]DMSO) spectrum of the product **4k**.

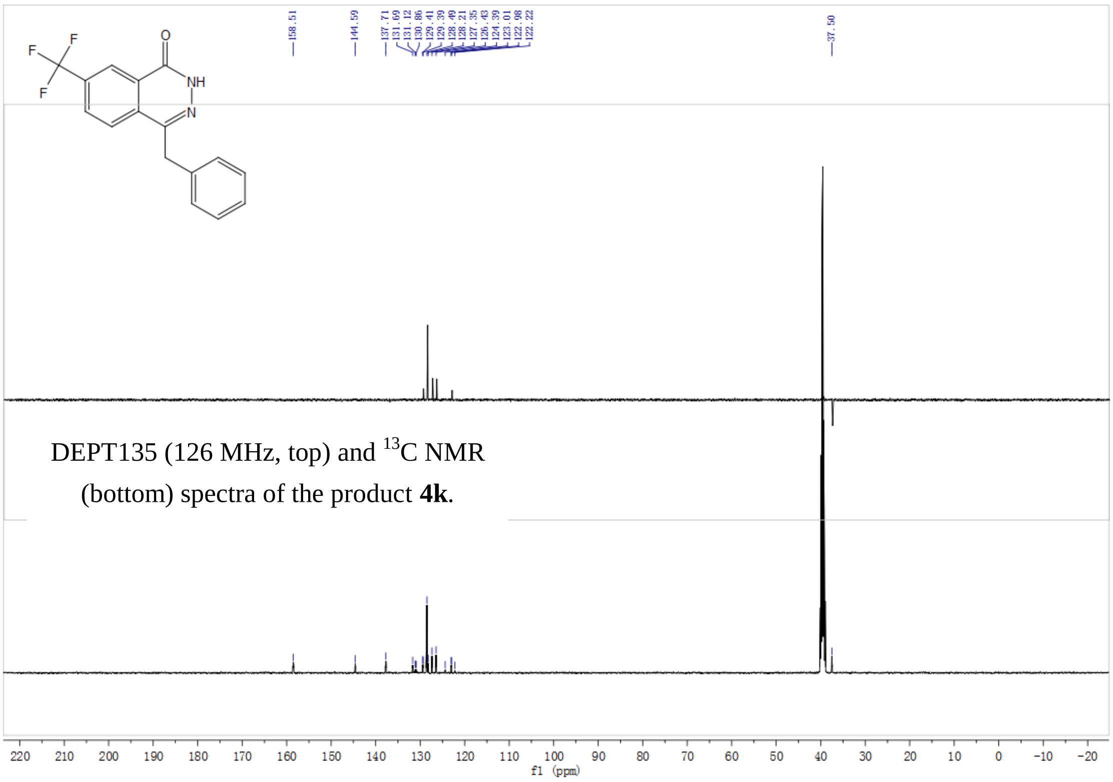

DEPT135 (126 MHz, top) and ^{13}C NMR (bottom) spectra of the product **4k**.

4-(4-Bromobenzyl)phthalazin-1(2*H*)-one (4p)

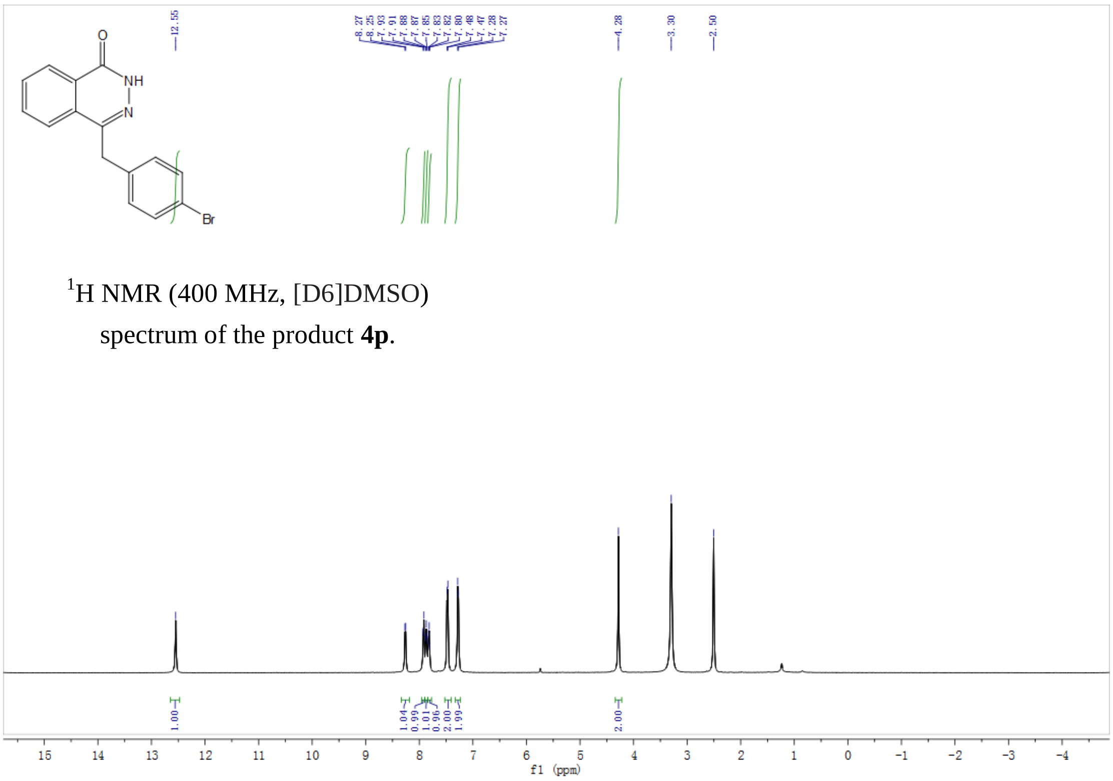

^{1}H NMR (400 MHz, [D6]DMSO) spectrum of the product **4p**.

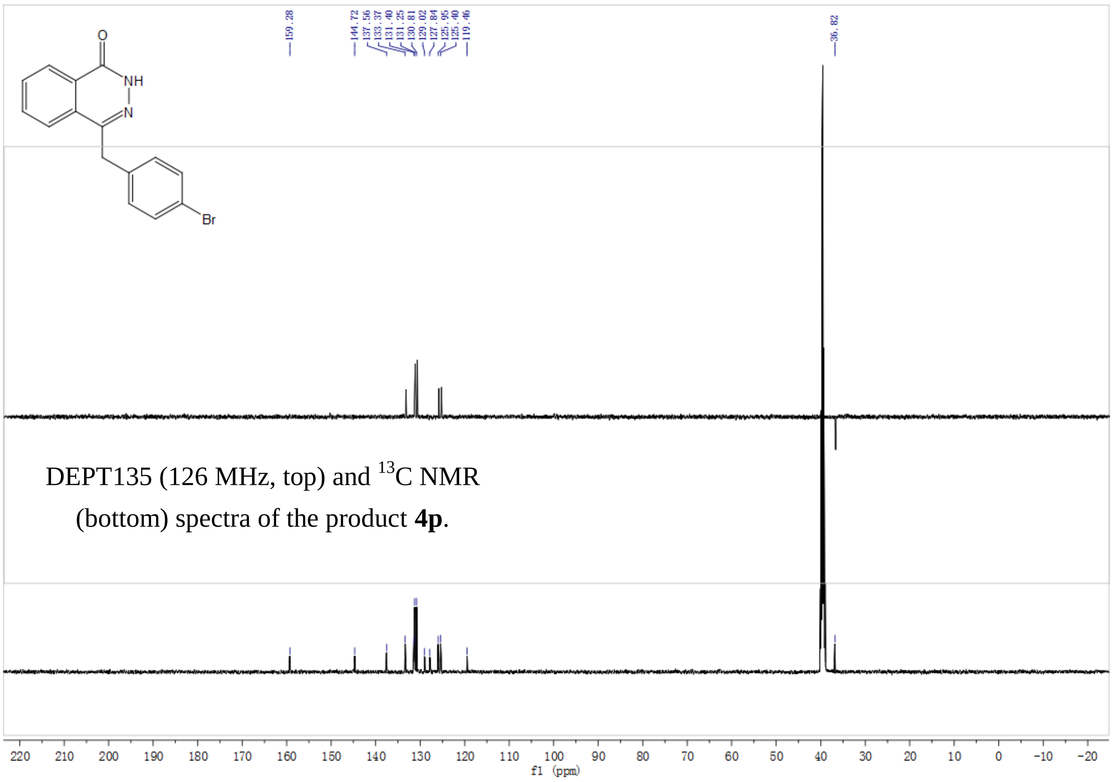

DEPT135 (126 MHz, top) and ^{13}C NMR (bottom) spectra of the product **4p**.

4-(Cyclopropylmethyl)phthalazin-1(2*H*)-one (4u)

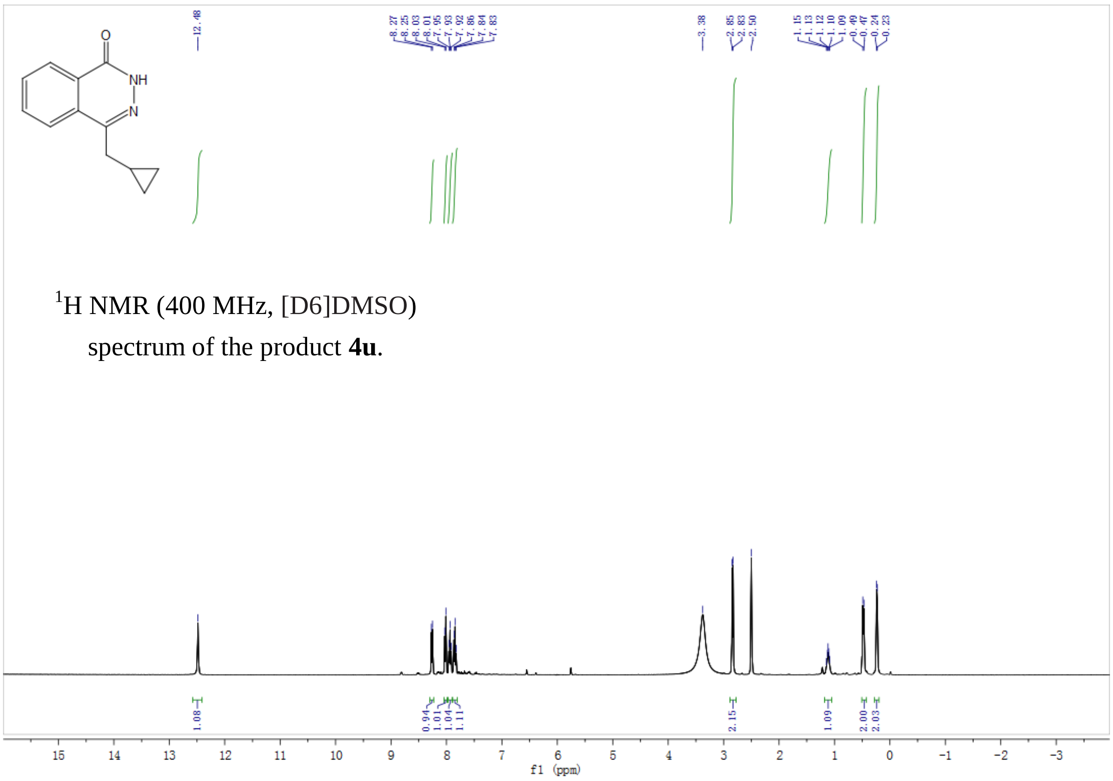

^{1}H NMR (400 MHz, [D6]DMSO) spectrum of the product **4u**.

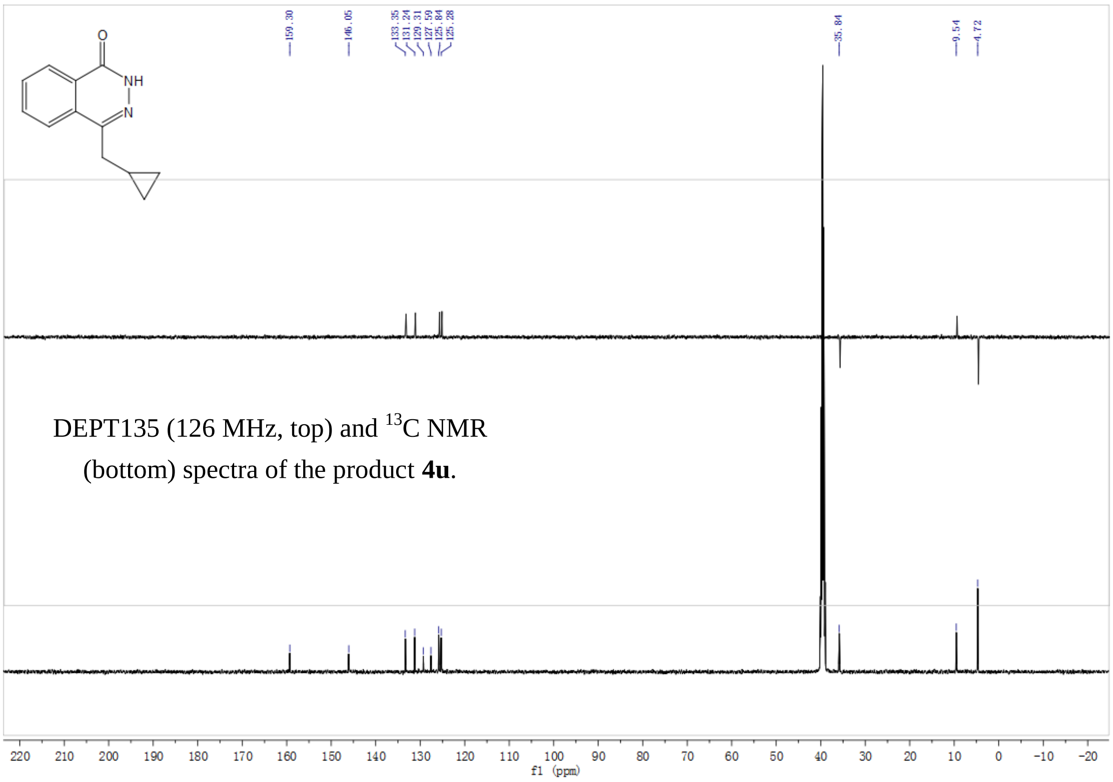

DEPT135 (126 MHz, top) and ^{13}C NMR (bottom) spectra of the product **4u**.
